# Synthesis and evaluation of antimicrobial activity, cytotoxic and pro-apoptotic effects of novel spiro-4*H*-pyran derivatives†

**DOI:** 10.1039/c9ra03196k

**Published:** 2019-08-09

**Authors:** Fatemeh Safari, Hajar Hosseini, Mohammad Bayat, Ashkan Ranjbar

**Affiliations:** Department of Biology, Faculty of Science, University of Guilan Rasht Iran fsafari@guilan.ac.ir; Department of Chemistry, Faculty of Science, Imam Khomeini International University Qazvin Iran bayat_mo@yahoo.com m.bayat@sci.ikiu.ac.ir +98 28 33780040

## Abstract

A new library of spiropyrans were synthesized *via* a one-pot four component reaction of cyanoacetohydrazide, ninhydrin, malononitrile and various cyclic CH-acids in EtOH at reflux conditions. The products were isolated and tested *in vitro* for antibacterial effects on *Escherichia coli* (*E. coli*) and *Staphylococcus aureus* (*S. aureus*). Furthermore cytotoxic activity of the spiropyrans on non-small-cell lung cancer cells (A549 cells), a breast epithelial cancer cell line (MCF-7), human malignant melanoma cells (A375), prostate cancer cells (PC3 cells, LNCaP cells) and normal cells HDF (human dermal fibroblast) was investigated. Interestingly, it was found that compounds 5a, 5b, 5f, 5g and 5i have the best MIC against *S. auerus* and compound 5a displayed the most potent activity against A549, A375, and LNCaP tumor cells. Moreover, DAPI staining of the A549 cancer cell lines that were treated with 5a were associated with the death of A549 cells. By using RT-PCR method, it was finally confirmed that apoptosis occurs in A549 cells by up-regulated Bax expression and down-regulated Bcl-2 expression from the mitochondrial pathway of apoptosis.

## Abstract 2

Evalution of anti-microbial activity, cytotoxic and pro-apoptotic effects of novel synthesized fused spiro-4*H*-pyran derivatives.

## Graphical abstract

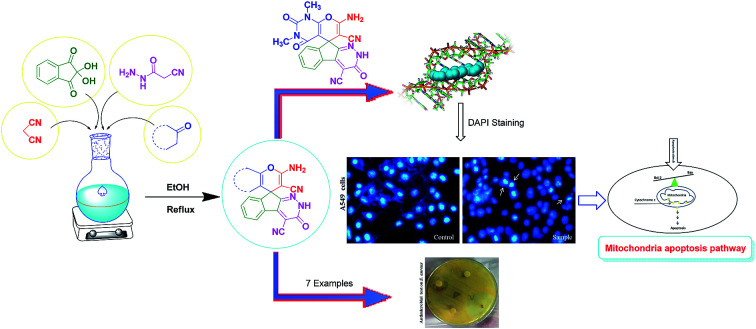

## Introduction

4*H*-pyrans and 4*H*-pyran-annulated heterocyclic frameworks represent an excellent structural motif that is often found in naturally occurring compounds^1–5^ with a broad spectrum of remarkable biological activities such as anticancer,^6^ cytotoxic,^7^ anti-HIV,^8,9^ anti-inflammatory,^10^ antimalarial,^11,12^ antimicrobial,^13^ antihyperglycemic and antidyslipidemic,^14^ and antineurodegenerative disorders like Alzheimer's, Parkinson's and Huntington's disease.^15^ Spiroheterocycles have also attracted much attention due to their effective pharmacological properties such as anticonvulsant,^16^ antimicrobial and antibacterial,^17,18^ anti-tubercular,^19^ anticancer^20^ and antioxidant.^21^

In recent years, a variety of synthetic approaches for constructing spiro-fused pyran derivatives *via* multicomponent reactions have been reported, particularly involving a three-component reaction of 1,3-diketones, aldehydes and malononitrile/ethyl cyanoacetate in the presence of various homo- and heterogeneous catalysts.^22–29^

Drug resistance is always a great challenge for researchers to encourage them to explore and design new drugs with the potential effectiveness in treatment of diseases including infectious diseases and cancer. To do so, we focused on derivatives of spiro-fused-pyran compounds. As mentioned, 4*H*-pyran derivatives and spiro compounds are the most important fragments in some efficient drugs with antimicrobial activity. In this regard, 10 compounds with spiro-4*H*-pyran nucleus were synthesized and evaluated for their antimicrobial activity against two different bacterial species using disk diffusion methods.

Activation of apoptotic pathways in cancer cells is considered a protective mechanism against the development and progression of cancer. Apoptosis, or programmed cell death, is a natural process which occurs in all living organisms including cancer cells for removing of unwanted cells. It was demonstrated that there are two main apoptotic pathways: the extrinsic or death receptor pathway and the intrinsic or mitochondrial pathway. Mitochondrial-mediated apoptosis is associated with the Bcl-2 family of proteins including anti-apoptotic such as Bcl-2, Bcl-XL, MCL-1 and pro-apoptotic such as Bax, Bid, Bak that governs membrane permeability of mitochondria to transmit pro-apoptotic signals to cell death.^30–32^

The synthetic spiroindenopyridazine-4*H*-pyran derivatives were investigated to determine their effects on the death of five types of cancer cells. The results showed that 5a was the most effective compound on A549, A375, and LNCaP cancer cells. Treatment of A549 cells with 5a induces mitochondrial-mediated apoptosis in A549 by induction of Bcl-2 expression and reduction of Bax expression.

## Results and discussion

### Chemistry

#### Total reaction

**Scheme 1:**
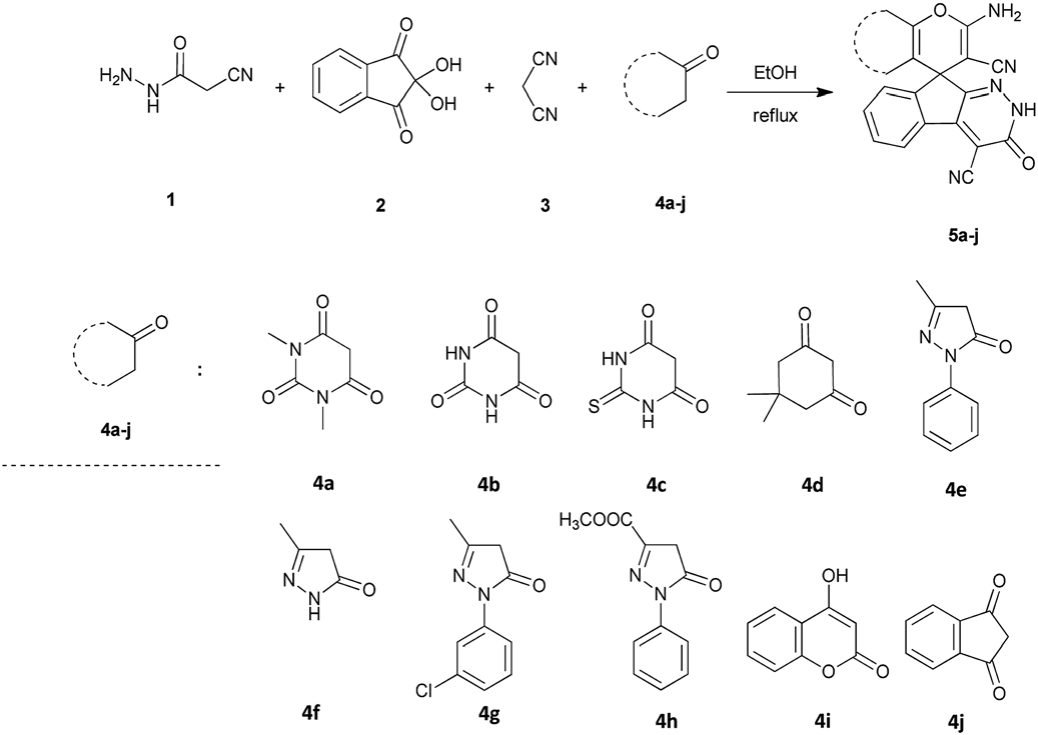
Synthesis of spiroindenopyridazine-4*H*-pyran derivatives 5a–j.

As shown in Scheme 1, a series of spiro[indeno[2,1-*c*]pyridazine-9,4′-pyran]-3′,4-dicarbonitrile derivatives 5a–j were synthesized by refluxing a mixture of cyanoacetohydrazide 1, ninhydrin 2, malononitrile 3 and various cyclic CH-acids 4a–j in ethanol under catalyst-free conditions in a one-pot procedure. The structures of products are presented in Table 1.

**Table 1:** Structure of products 5a–j^a^

Entry	CH-acid	Product	Time (h)	Yield (%)	Mp (°C)
1	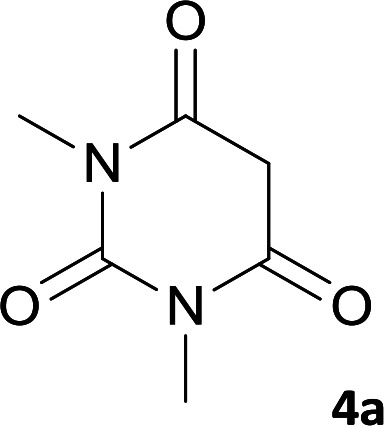	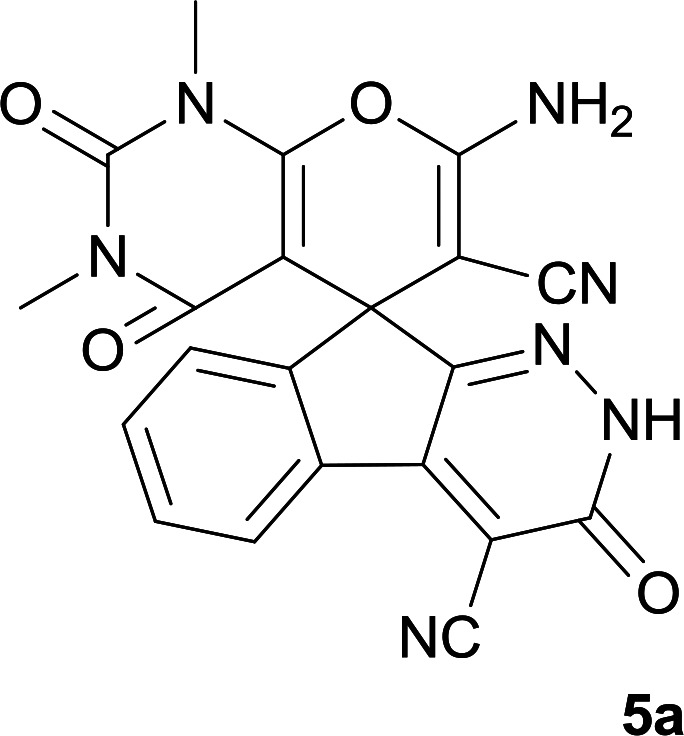	7	95	282–285
2	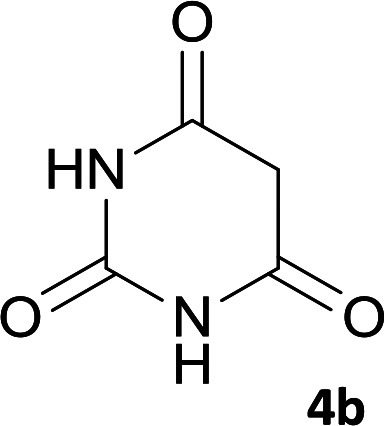	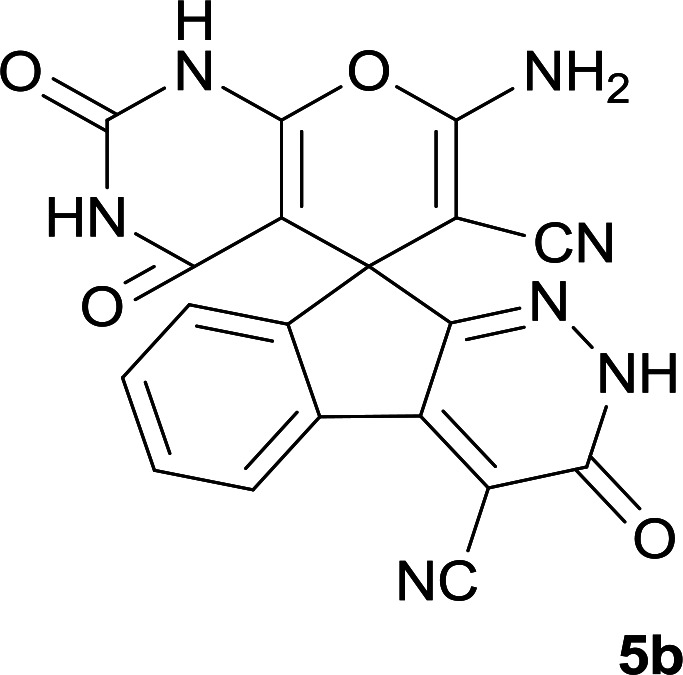	7	97	322–324
3	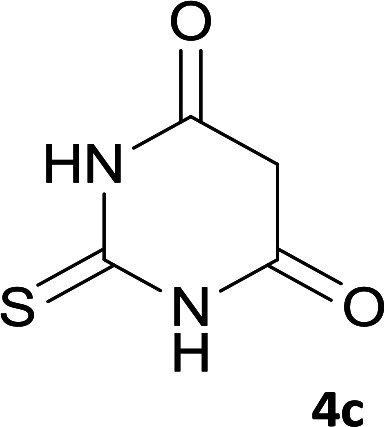	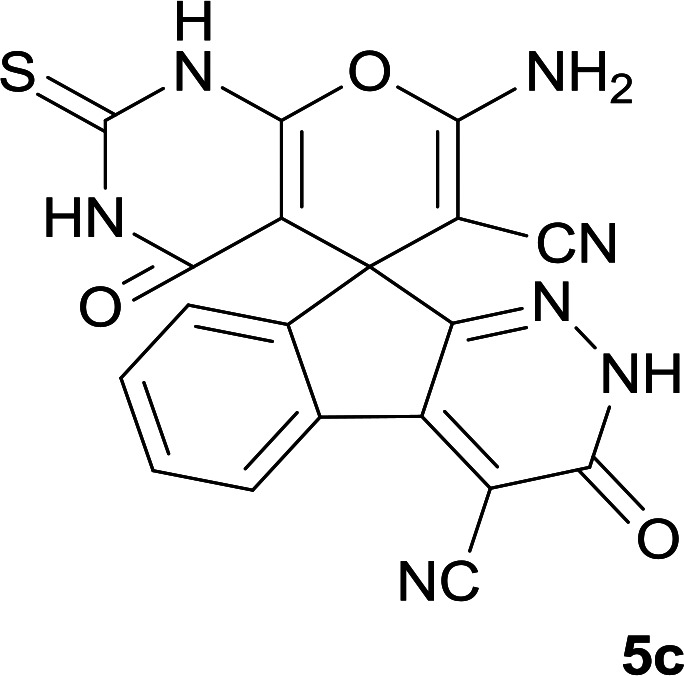	8	93	305–307
4	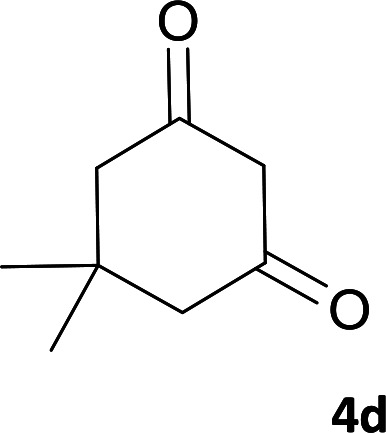	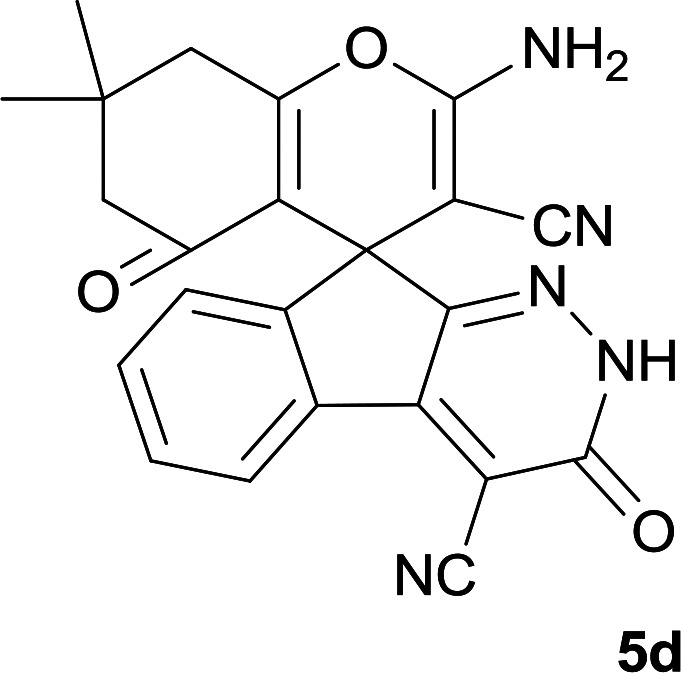	6	98	315–318
5	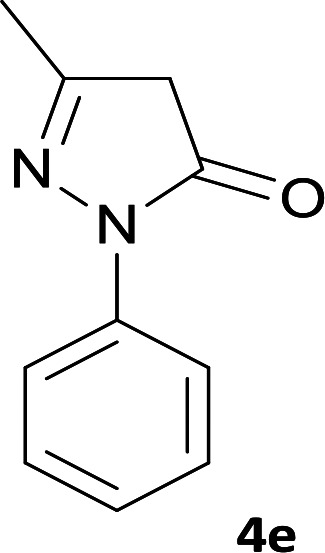	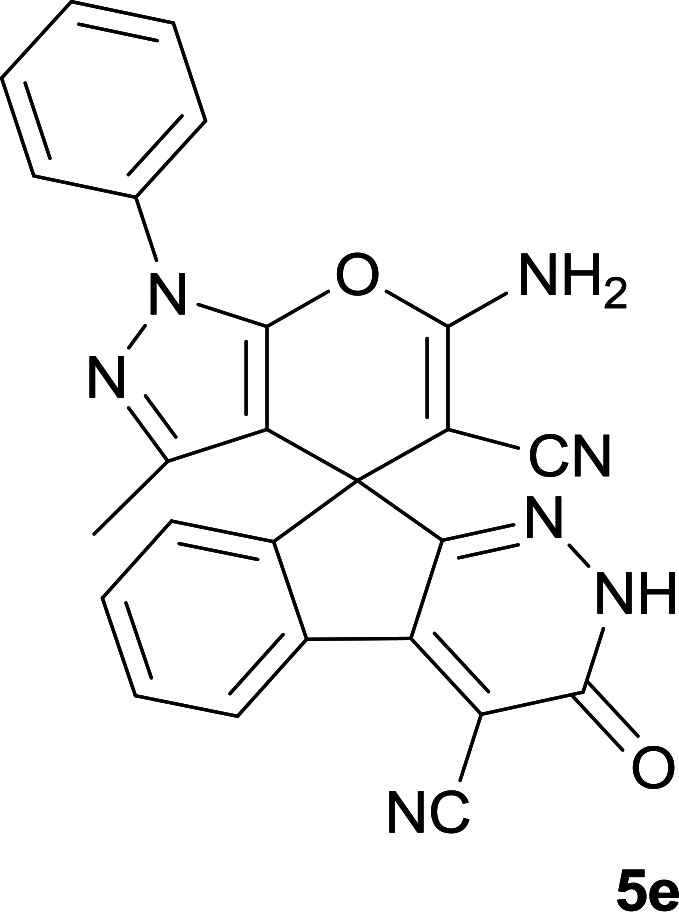	10	97	280–282
6	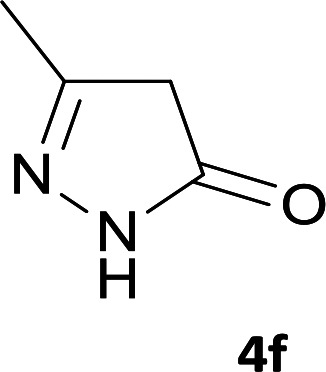	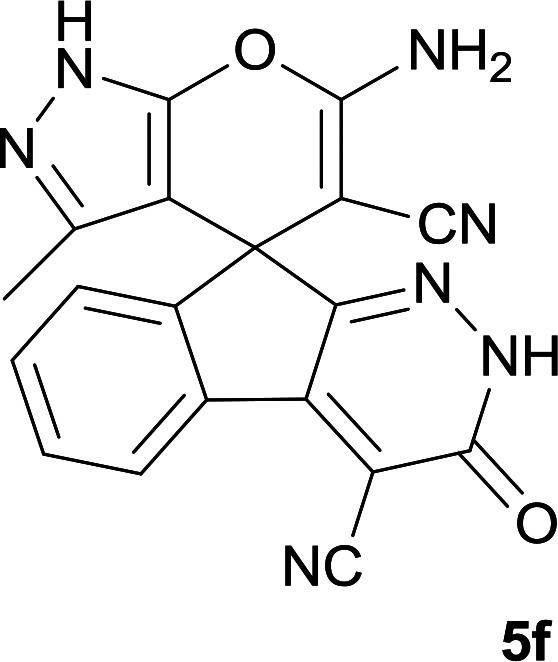	10	93	295–297
7	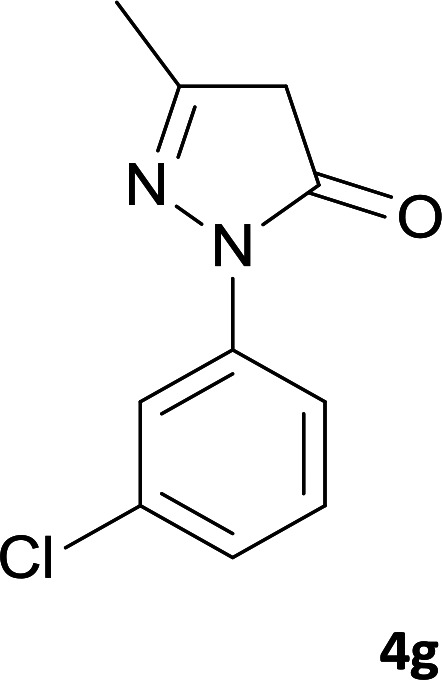	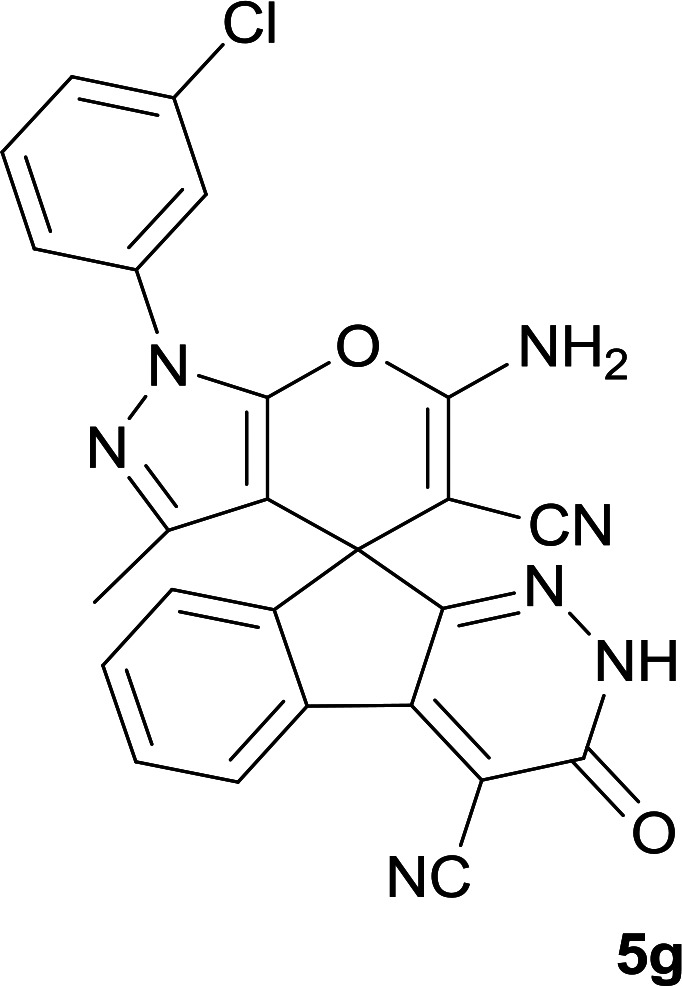	9	98	266–268
8	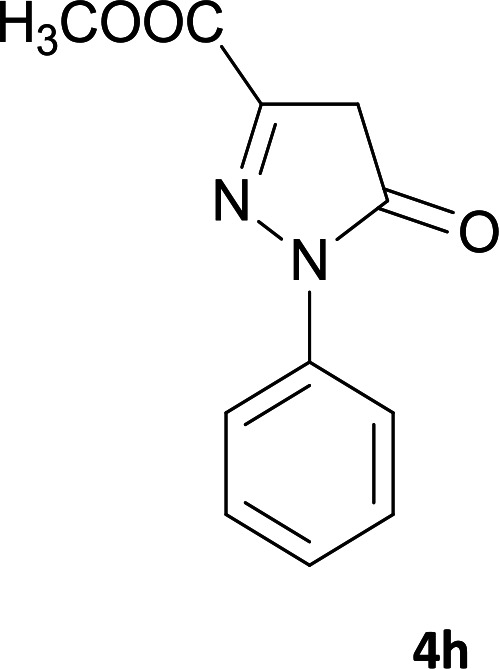	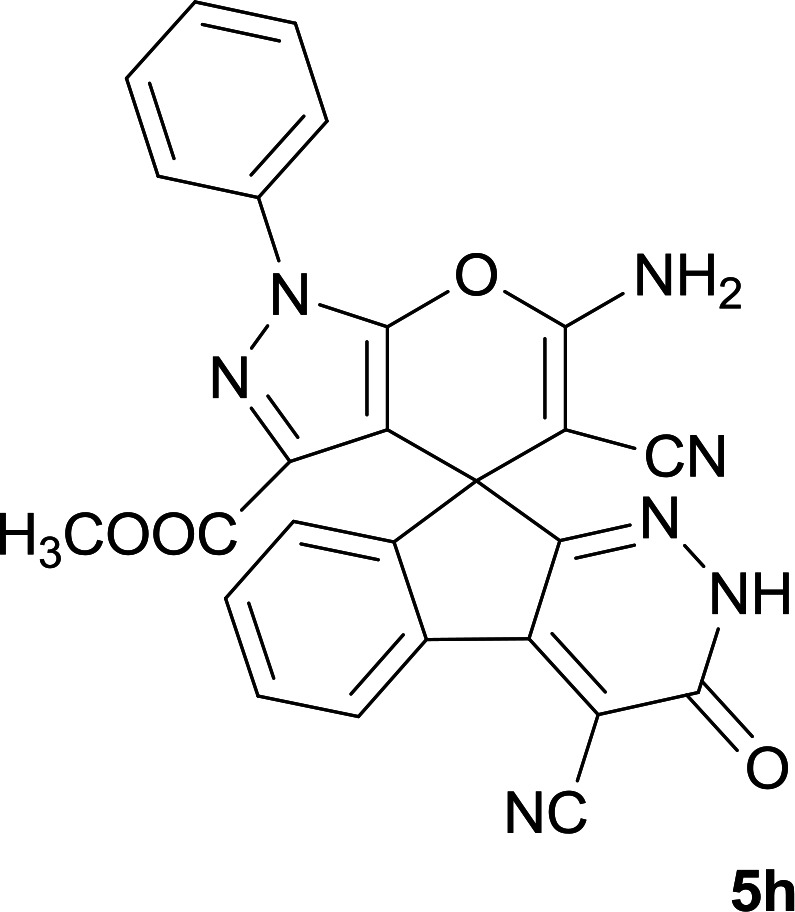	10	96	290–293
9	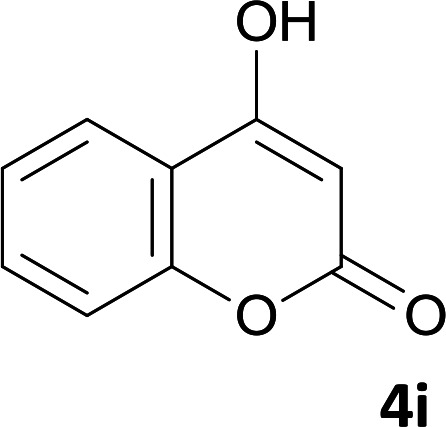	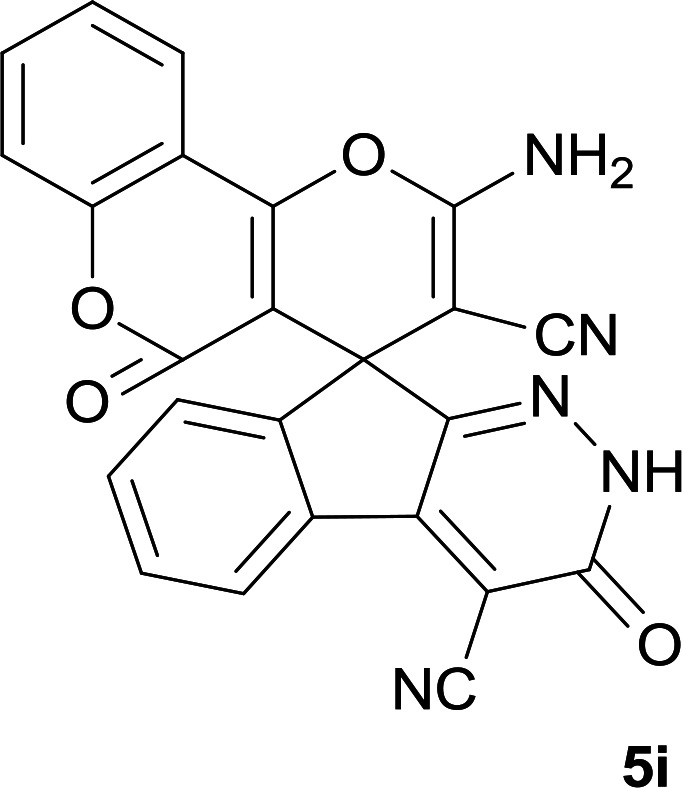	12	90	308–310
10	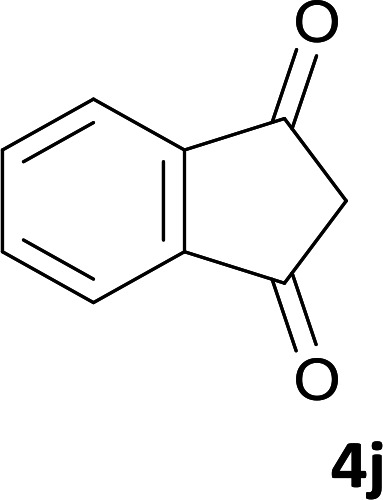	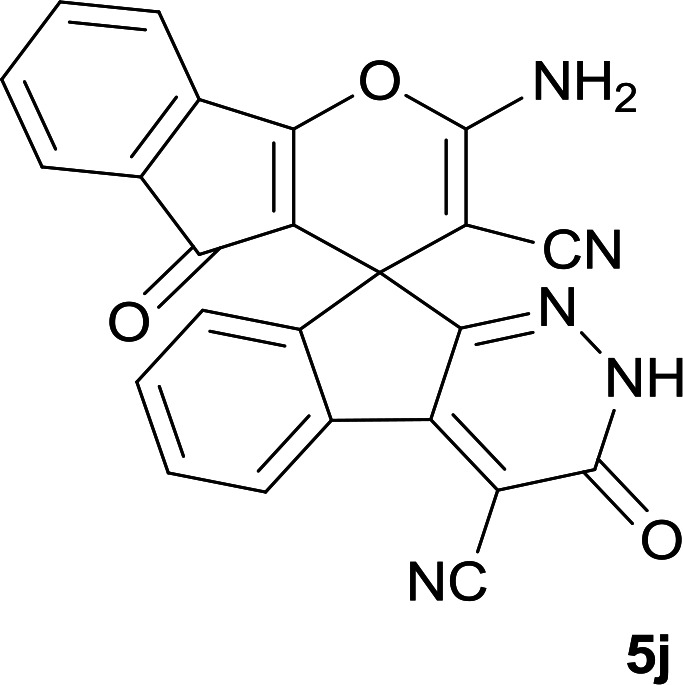	9	92	298–300

a The reaction was performed using cyanoacetohydrazide (1 mmol), ninhydrin (1 mmol), malononitrile (1 mmol), CH-acid (1 mmol), EtOH (10 mL) and reflux.

#### Structure determination

**Fig. 1:**
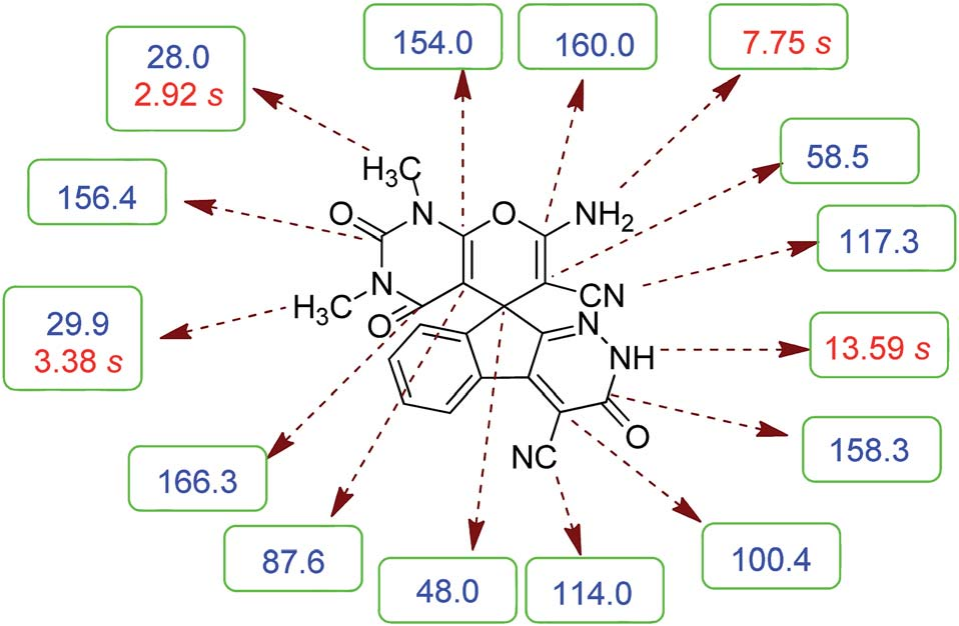
^1^H and ^13^C NMR chemical shifts of 5a.

Confirmation of the structure and purity of these analogs was obtained from ^1^H- and ^13^C-NMR and mass spectroscopic analysis. As a representative example, as seen in Fig. 1, the ^1^H NMR spectrum of 5a showed two signals identified as two methyl groups (*δ* 2.92, 3.38 ppm), multiplet signals for the aromatic region (*δ* 7.60–8.17 ppm), the amino group (*δ* 7.75 ppm) and the NH group (*δ* 13.59 ppm). The characteristic signals in ^1^H-decoupled ^13^C NMR spectrum are also demonstrated in Fig. 1. The spiro carbon and *C*=CNH_2_ were observed at *δ* 48.0 and 58.5 ppm respectively. Two signals at *δ* 160.0 and 154.0 ppm were related to two carbons in position 2 and 6 of pyran ring respectively. Three carbonyl groups appeared at *δ* 166.3, 158.3 and 156.4 ppm. The signal at *δ* 87.6 ppm was assigned to C-5 of pyran ring. Two nitrile groups were observed at *δ* 114.0, 117.3 ppm. The mass spectrum of 5a displayed the molecular-ion peak at *m*/*z* 427 in agreement with the suggested structure. The IR spectrum of this product showed absorption bands due to NH, NH_2_, CN and CO groups at 3378, 3309, 3200, 2217, 1675, 1642 cm^−1^ respectively.

#### Mechanism

**Scheme 2:**
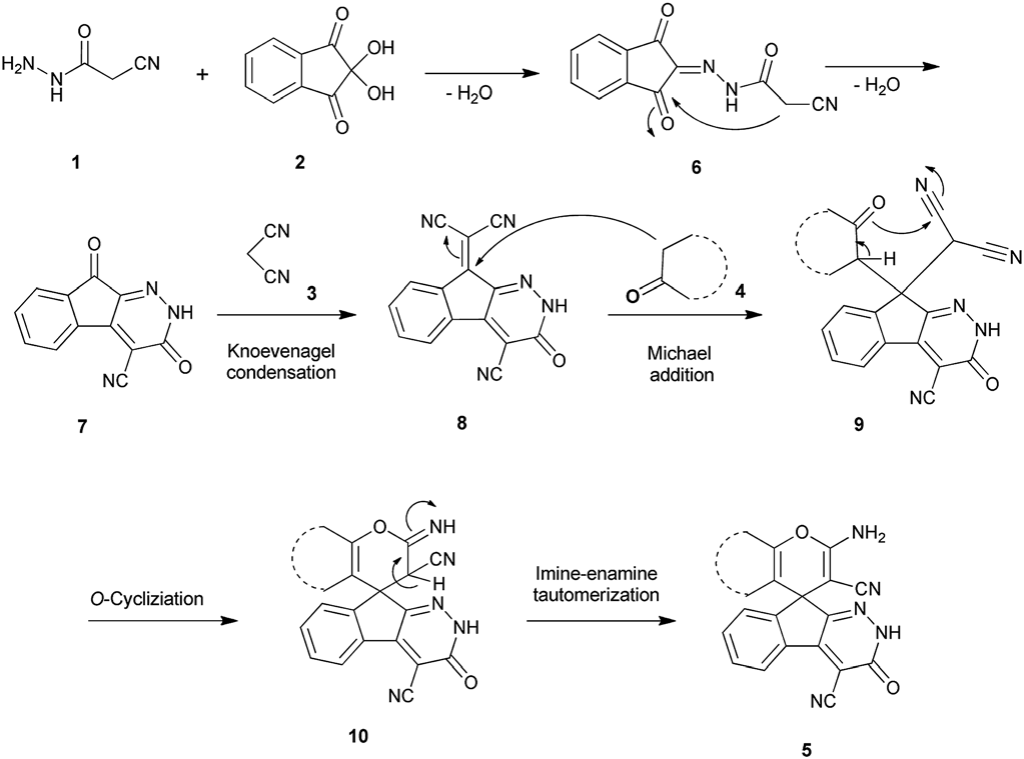
Proposed mechanism for the formation of products 5.

An acceptable mechanism for the construction of spiroindenopyridazines 5 is shown in Scheme 2. Initially, condensation of cyanoacetohydrazide 1 and ninhydrin 2 leads to intermediate 6, which undergoes intramolecular cyclization to give the corresponding indeno[2,1-*c*]pyridazine 7. Subsequent addition of malononitrile 3 to indeno[2,1-*c*]pyridazine 7 affords intermediate 8. Michael addition of CH-acid 4 on Knoevenagel adduct 8 leads to intermediate 9, which undergoes successive keto–enol tautomerization followed by *O*-cyclization *via* nucleophilic addition of oxygen to nitrile group that produces intermediate 10. Finally imine–enamine tautomerization of 10 affords the desired structures 5 (Scheme 2).

### Biology

#### Antibacterial test and MICs determination

Antibacterial effects of spiro[indeno[2,1-*c*]pyridazine-9,4′-pyran]-3′,4-dicarbonitrile derivatives 5a–j were evaluated *in vitro* by using disk diffusion method against Gram-positive (*S. aureus*) and Gram negative (*E. coli*). The diameter of inhibition zone correlates to the sensitivity of a bacterium to an antibacterial agent. A larger inhibition zone means that the antimicrobial is more potent. Disk diffusion data showed an inhibition zone 4–15 mm for the synthetic compounds against *S. aureus* (except 5d, 5h and 5j). *E. coli* was resistant against all compounds tested (Table 2). Tetracycline and DMSO were used as positive control and negative control respectively. Next, the active compounds were analysed using MIC assay to determine the lowest concentration for inhibition. A lower MIC value indicates that less drug is required for inhibiting growth of the organism; therefore, compounds with lower MIC values are more effective antimicrobial agents. The MIC scores of target compounds listed in Table 3. In the case of inhibitory effects of 7 active structures on *S. aureus*, compounds 5a, 5b, 5f, 5g and 5i showed the lowest MICs. It seems due to the difference in structure of bacterial cell wall (*i.e.*, the presence of outer membrane with lipid, lipopolysaccharide, and lipoprotein content in Gram negative bacteria), *E. coli* was resistant against the related synthetic compounds.

**Table 2:** Antimicrobial activity of synthesized compounds by using disc diffusion test at concentration 50 mM (diameter of zone of inhibition in mm)^a^

Compounds	Inhibition zone (mm)	
	*S. aureus*	*E. coli*
5a	7 mm	—
5b	5 mm	—
5c	7 mm	—
5d	—	—
5e	9 mm	—
5f	5 mm	—
5g	15 mm	—
5h	—	—
5i	4 mm	—
5j	—	—
Tetracycline	30 mm	23 mm
DMSO	0	0

a —: inactive at concentration 50 mM.

**Table 3:** Comparison of the minimum inhibitory concentrations (MICs) of synthesized compounds^a^

Compounds	MICs (mM) *S. aureus*
5a	5 mM
5b	5 mM
5c	50 mM
5e	10 mM
5f	5 mM
5g	5 mM
5i	5 mM

#### Cytotoxic activity

**Fig. 2:**
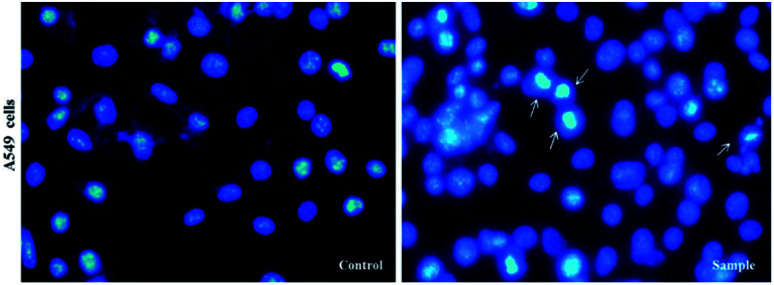
Inverted fluorescent microscopy images of chromatin damages occurrence in the nucleus of treated cells with 5a compound and DMSO (1%), which have been stained with DAPI in A549 cancer cell lines. The experiments were performed three times (original microscope magnification, 40×, scale bar, 10 μm).

At first, in order to compare anti-cancer properties, the synthesized compounds 5a–j were evaluated against A549 cells, MCF-7 cells, A375 cells, PC3 cells, LNCaP cells and HDF cells lines using the MTT colorimetric assay. The activity is expressed as 50% growth inhibitory concentration (IC_50_) values at 48 h. The results are presented in Table 4. We used DMSO (1%) as negative control and Etoposide as positive control. Among the different heterocyclic compounds in terms of chemical structure, compound 5a showed the highest cytotoxicity against A549 cell line (IC_50_ = 40 μM), A375 cell line (IC_50_ = 70.72 μM), and LNCaP cell line (IC_50_ = 32.15 μM). Next, by using DAPI staining, we showed clear morphological changes and fragmentation in the chromatin within the nucleus of A549 treated cells, but their morphology is not altered in untreated cells (or control). Inverted fluorescent microscopy images indicated that compound 5a induced cell death in A549 cells (Fig. 2).

**Table 4:** *In vitro* cytotoxic activities of compounds 5a–j against cancer cell lines, A549, PC3, and MCF-7, A375, LNCaP and normal cell HDF (human dermal fibroblast)^a^

Compounds	Cell lines (IC_50_, μm)					
	A549 cells	PC3 cells	MCF-7 cells	A375 cells	LNCaP cells	HDF cells
5a	**40±0.007**	**>100**	**>100**	**70.72±0.064**	**32.15±0.046**	>100
5b	>100	>100	>100	>100	>100	>100
5c	>100	>100	>100	>100	>100	>100
5d	>100	>100	>100	>100	>100	>100
5e	>100	>100	>100	>100	>100	>100
5f	>100	>100	>100	>100	>100	>100
5g	>100	>100	>100	>100	>100	>100
5h	>100	>100	>100	>100	>100	>100
5i	>100	>100	>100	>100	>100	>100
5j	>100	>100	>100	>100	>100	>100
**Etoposide**	**60±0.015**	**40±0.062**	**30±0.011**	**25.31±0.018**	**90±0.031**	>100

a Data represent mean ± SD of three independent experiments.

**Fig. 3:**
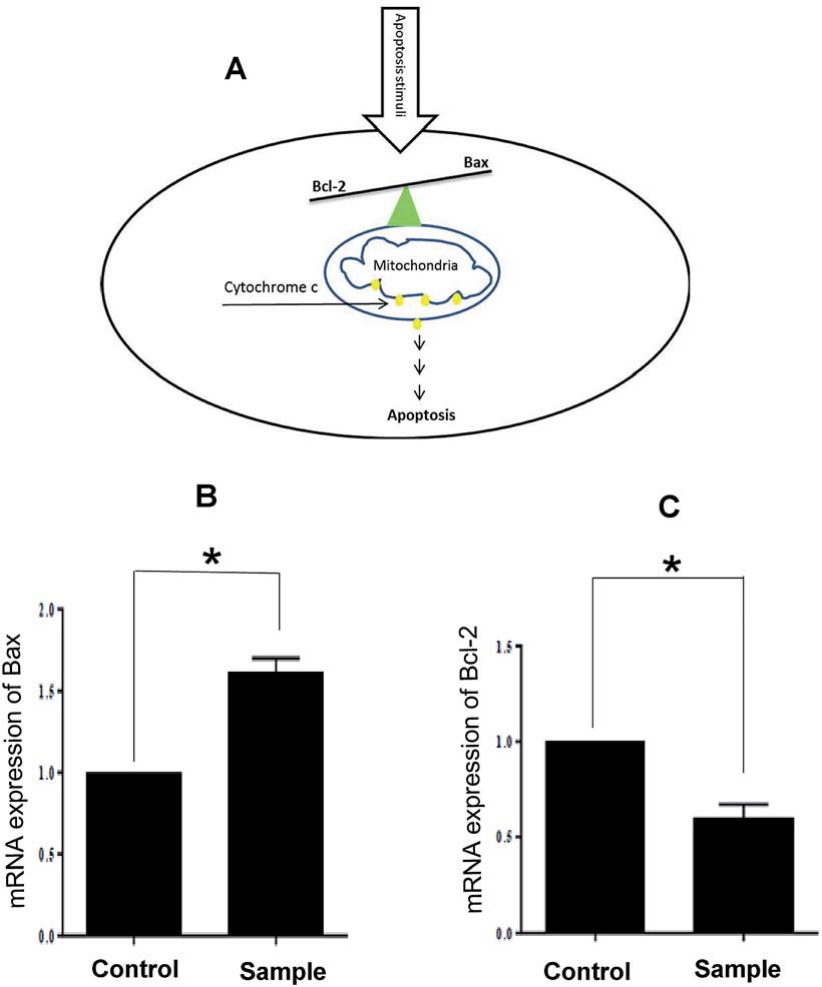
(A) Schematic model for activation of apoptosis in the cell; (B) and (C) relative expression of Bax and Bcl-2 mRNA of treated A549 cancer cells with 5a compound. Data represent mean ± SD of three independent experiments. *p* < 0.05 was considered to be statistically significant.

Finally, we confirmed that apoptosis mediated by mitochondria induces in A549 cells. It is well known that the anti-apoptotic Bcl-2 protein is localized on the outer mitochondrial membranes and antagonizes the action of pro-apoptotic Bax. The balance between these two proteins prevents translocation of cytochrome c from the mitochondria to cytoplasm and determines the apoptosis resistance. Down-regulation of Bcl-2 and/or up-regulation of Bax disturb this balance and leads to apoptosis (Fig. 3A). Our results demonstrated that our compounds have no inhibition effect on two cancer cell lines, MCF-7, PC3 and normal cells, HDF. Interestingly, compound 5a has inhibition effect on A549 and LNCaP cancer cells at the concentration lower than Etoposide (A549 cells: IC50 = 40 μM in 5a and IC_50_ = 60 μM in Etoposide; LNCaP cells: IC_50_ = 32.15 μM in 5a and IC_50_ = 90 μM in Etoposide). Treatment with 5a leads to both up-regulated expression of Bax and down-regulation expression of Bcl-2 in A549 cancer cells (Fig. 3B and C).

## Chemical experiments

### Materials

All commercially available reagents and other solvents were purchased from Aldrich and Merck chemical Co. and used without further purification. The NMR spectra were recorded with a Bruker DRX-300 AVANCE instrument (300 MHz for ^1^H and 75.4 MHz for ^13^C) with DMSO-*d*_6_ as solvent. Chemical shifts are given in ppm (*δ*) relative to internal TMS, and coupling constant (*J*) are reported in Hertz (Hz). Melting points were measured with an electrotherma1 9100 apparatus. Mass spectra were recorded with an Agilent 5975C VL MSD with Triple-Axis detector operating at an ionization potential of 70 eV. IR spectra were measured with Bruker Tensor 27 spectrometer.

### General procedure of the synthesis of spiroindenopyridazine-4*H*-pyran compounds

The stoichiometric mixture of cyanoacetohydrazide 1 (1 mmol), ninhydrin 2 (1 mmol), malononitrile 3 (1 mmol) and cyclic CH-acid 4 (1 mmol) in EtOH (10 mL) was stirred at reflux temperature. The progress of the reaction was monitored by TLC using ethyl acetate-*n*-hexane (1 : 1) as an eluent. After completion of the reaction, mixture was cooled to room temperature and crude products were isolated by simple filtration. The precipitated products or crystals in some cases, were collected by filtration and washed with EtOH to give the pure product 5a–j.

### Spectral data

#### 7′-Amino-1′,3′-dimethyl-2′,3,4′-trioxo-1′,2,2′,3,3′,4′-hexahydrospiro[indeno[2,1-*c*]pyridazine-9,5′-pyrano[2,3-*d*]pyrimidine]-4,6′-dicarbonitrile (5a)

Pale yellow crystals; yield: 0.408 g (95%); mp: 282–285 °C; IR (KBr): 3378, 3309, 3200, 3063, 2885, 2217, 1721, 1675, 1642, 1496, 1387, 1190, 759, 507 cm^−1^; ^1^H NMR (300 MHz, DMSO-*d*_6_): *δ* = 13.59 (1H, s, NH), 8.15 (1H, d, *J* = 7.5 Hz, ArH), 7.75 (2H, s, NH_2_), 7.60–7.72 (3H, m, ArH), 3.38 (3H, s, NCH_3_), 2.92 (3H, s, NCH_3_); ^13^C NMR (75.4 MHz, DMSO-*d*_6_): *δ* = 166.3 (CONMe), 160.0 (CNH_2_), 158.3 (CONH), 156.4 (CO(NMe)_2_), 154.0 (CNMe), 152.6 (C=N), 150.0 (*C*C=N), 148.4, 135.5, 131.8, 130.3, 126.1, 124.4, 117.3 (CN), 114.0 (CN), 100.4 (*C*CONH), 87.6 (*C*CONMe), 58.5 (*C*=CNH_2_), 48.0 (C_spiro_), 29.9 (NCH_3_), 28.0 (NCH_3_); MS (EI, 70 eV): *m*/*z* (%) = 427 (1.22) [M]^+^, 411 (2), 363 (7), 332 (7), 271 (100), 243 (43), 215 (73), 188 (64), 156 (22), 124 (9), 99 (15), 75 (13), 56 (14).

#### 7′-Amino-2′,3,4′-trioxo-1′,2,2′,3,3′,4′-hexahydrospiro[indeno[2,1-*c*]pyridazine-9,5′-pyrano[2,3-*d*]pyrimidine]-4,6′-dicarbonitrile (5b)

Light yellow crystals; yield: 0.387 g (97%); mp: 322–324 °C; IR (KBr): 3466, 3371, 3308, 3196, 3109, 3021, 2896, 2819, 2201, 1741, 1659, 1529, 1393, 1123, 761, 598 cm^−1^; ^1^H NMR (300 MHz, DMSO-*d*_6_): *δ* = 13.59 (s, 1H, NH), 12.46 (1H, br, NH), 11.11 (1H, s, NH), 8.14 (1H, d, *J* = 7.5 Hz, ArH), 7.55–7.74 (3H, m, ArH), 7.45 (2H, s, NH_2_); ^13^C NMR (75.4 MHz, DMSO-*d*_6_): *δ* = 161.9 (CNH_2_), 158.4 (CONH), 158.3 (CONHN), 156.4 (CO(NH)_2_), 153.9 (*C*NHCO), 153.9 (C=N), 149.6 (*C*C=N), 148.3, 135.5, 131.8, 130.2, 126.1, 124.4, 117.5 (CN), 114.0 (CN), 100.3 (*C*CONHN), 87.4 (*C*CONH), 58.6 (*C*=CNH_2_), 47.2 (C_spiro_); MS (EI, 70 eV): *m*/*z* (%) = 333 (0.01), 271 (100), 243 (48), 215 (83), 188 (85), 161 (12), 124 (10), 100 (12), 75 (10), 50 (9).

#### 7′-Amino-3,4′-dioxo-2′-thioxo-1′,2,2′,3,3′,4′-hexahydrospiro[indeno[2,1-*c*]pyridazine-9,5′-pyrano[2,3-*d*]pyrimidine]-4,6′-dicarbonitrile (5c)

White powder; yield: 0.385 g (93%); mp: 305–307 °C; IR (KBr): 3305, 3168, 3069, 2918, 2359, 2201, 1676, 1648, 1559, 1391, 1339, 1170, 1124, 764, 575 cm^−1^; ^1^H NMR (300 MHz, DMSO-*d*_6_): *δ* = 13.61 (1H, s, NH), 12.40 (1H, s, NH), 12.16 (1H, s, NH), 8.14 (1H, d, *J* = 7.5 Hz, ArH), 7.63–7.70 (3H, m, ArH), 7.57 (2H, s, NH_2_); ^13^C NMR (75.4 MHz, DMSO-*d*_6_): *δ* = 174.7 (C=S), 159.7 (CNH_2_), 158.3 (CONH), 158.3 (CONHN), 156.0 (CNHCS), 153.5 (C=N), 153.4 (*C*C=N), 148.3, 135.5, 131.9, 130.4, 126.3, 124.4, 117.4 (CN), 114.0 (CN), 100.4 (*C*CONHN), 92.1 (*C*CONH), 58.2 (*C*=CNH_2_), 47.2 (C_spiro_); MS (EI, 70 eV): *m*/*z* (%) = 415 (0.04) [M]^+^, 390 (0.03), 349 (0.07), 271 (100), 243 (36), 215 (76), 188 (66), 163 (5), 144 (37), 116 (17), 87 (7), 59 (8).

#### 2-Amino-7,7-dimethyl-3′,5-dioxo-2′,3′,5,6,7,8-hexahydrospiro[chromene-4,9′-indeno[2,1-*c*]pyridazine]-3,4′-dicarbonitrile(5d)

Yellow crystals; yield: 0.407 g (98%); mp: 315–318 °C; IR (KBr): 3477, 3333, 3167, 3011, 2960, 2882, 2359, 2196, 1653, 1596, 1356, 1315, 1162, 1048, 762, 695 cm^−1^; ^1^H NMR (300 MHz, DMSO-*d*_6_): *δ* = 13.52 (1H, s, NH), 8.15 (1H, d, *J* = 7.8 Hz, ArH), 7.58–7.74 (2H, m, ArH), 7.52 (1H, d, *J* = 7.5 Hz, ArH), 7.43 (2H, s, NH_2_), 2.60 (2H, s, CH_2_), 2.07 (2H, m, CH_2_), 1.05 (3H, s, CH_3_), 1.03 (3H, s, CH_3_); ^13^C NMR (75.4 MHz, DMSO-*d*_6_): *δ* = 195.8 (*C*OCH_2_), 165.3 (CNH_2_), 158.9 (CONH), 158.3 (C=*C*CH_2_), 157.1 (C=NNH), 154.4 (*C*C=NNH), 148.4, 135.6, 131.7, 130.0, 125.5, 124.5, 117.8 (CN), 114.0 (CN), 111.2 (*C*COCH_2_), 100.3 (*C*CONH), 58.3 (*C*=CNH_2_), 50.5 (CH_2_), 47.4 (C_spiro_), 40.7 (CH_2_), 32.4 (CMe_2_), 27.9 (CH_3_), 27.5 (CH_3_); MS (EI, 70 eV): *m*/*z* (%) = 411 (100) [M]^+^, 385 (26), 355 (3), 327 (30), 299 (31), 271 (37), 243 (16), 215 (33), 188 (32), 164 (12), 140 (7), 112 (8), 83 (18), 55 (18).

#### 6′-Amino-3′-methyl-3-oxo-1′-phenyl-2,3-dihydro-1′*H*-spiro[indeno[2,1-*c*]pyridazine-9,4′-pyrano[2,3-*c*]pyrazole]-4,5′-dicarbonitrile (5e)

Bright yellow crystal; yield: 0.436 g (97%); mp: 280–282 °C; IR (KBr): 3411, 3355, 3318, 3194, 2237, 2197, 1655, 1593, 1522, 1394, 1125, 762, 695 cm^−1^; ^1^H NMR (300 MHz, DMSO-*d*_6_): *δ* = 13.68 (1H, s, NH), 8.24 (1H, d, *J* = 7.8 Hz, ArH), 7.31–7.81 (8H, m, ArH), 7.77 (2H, s, NH_2_), 1.32 (3H, s, CH_3_); ^13^C NMR (75.4 MHz, DMSO-*d*_6_): *δ* = 161.0 (CNH_2_), 158.4 (CONH), 155.1 (*C*N-Ph), 152.7 (C=NNH), 146.7 (*C*Me), 145.2 (*C*C=NNH), 144.4 (C_ipso_), 137.6, 136.0, 131.4, 130.9, 129.9, 127.1, 127.0, 124.8, 120.7, 118.3 (CN), 114.1 (CN), 101.7 (*C*CONH), 97.1 (*C*CMe), 57.9 (*C*=CNH_2_), 48.1 (C_spiro_), 12.5 (CH_3_); MS (EI, 70 eV): *m*/*z* (%) = 445 (24) [M]^+^, 419 (8), 379 (83), 350 (13), 271 (62), 243 (23), 215 (49), 188 (59), 164 (17), 132 (6), 105 (14), 77 (100), 51 (36); anal. calcd for C_25_H_15_N_7_O_2_:C, 67.41; H, 3.39; N, 22.01. Found: C, 67.7; H, 3.6; N, 22.1.

#### 6′-Amino-3′-methyl-3-oxo-2,3-dihydro-1′*H*-spiro[indeno[2,1-*c*]pyridazine-9,4′-pyrano[2,3-*c*]pyrazole]-4,5′-dicarbonitrile (5f)

Light green powder; yield: 0.335 g (93%); mp: 295–297 °C; IR (KBr): 3459, 3270, 3140, 2234, 2184, 1674, 1636, 1594, 1391, 1154, 761, 696 cm^−1^; ^1^H NMR (300 MHz, DMSO-*d*_6_): *δ* = 13.58 (1H, s, NH), 12.33 (1H, s, NH), 8.21 (1H, d, *J* = 7.03 Hz, ArH), 7.59–8.09 (2H, m, ArH), 7.44 (1H, d, *J* = 7.5 Hz, ArH), 7.34 (2H, s, NH_2_), 1.32 (3H, s, CH_3_); ^13^C NMR (75.4 MHz, DMSO-*d*_6_): *δ* = 162.5 (CNH_2_), 158.3 (CONH), 155.8 (C=*C*NH), 155.4 (C=NNH), 153.3 (*C*Me), 146.6 (*C*=CCO), 135.9, 135.5, 131.2, 130.6, 126.6, 124.7, 119.0 (CN), 114.1 (CN), 101.5 (*C*CONH), 96.3 (*C*CMe), 56.9 (*C*=CNH_2_), 47.8 (C_spiro_), 9.6 (CH_3_); MS (EI, 70 eV): *m*/*z* (%) = 369 (5) [M]^+^, 243 (4), 211 (10), 152 (14), 109 (18), 83 (100), 43 (75); anal. calcd for C_19_H_11_N_7_O_2_: C, 61.79; H, 3.00; N, 26.55. Found: C, 61.4; H, 3.4; N, 26.7.

#### 6′-Amino-1′-(3-chlorophenyl)-3′-methyl-3-oxo-2,3-dihydro-1′*H*-spiro[indeno[2,1-*c*]pyridazine-9,4′-pyrano[2,3-*c*]pyrazole]-4,5′-dicarbonitrile (5g)

Bright yellow crystals; yield: 0.469 g (98%); mp: 266–268 °C; IR (KBr): 3384, 3322, 3200, 3079, 2358, 2195, 1659, 1587, 1395, 1162, 1071, 758, 694 cm^−1^; ^1^H NMR (300 MHz, DMSO-*d*_6_): *δ* = 13.69 (1H, s, NH), 8.24 (1H, d, *J* = 7.5 Hz, ArH), 7.39–7.81 (7H, m, ArH), 7.75 (2H, s, NH_2_), 1.32 (3H, s, CH_3_); ^13^C NMR (75.4 MHz, DMSO-*d*_6_): *δ* = 160.9 (CNH_2_), 158.4 (CONH), 155.0 (*C*NAr), 152.6 (C=NNH), 146.7 (*C*Me), 145.4 (*C*C=NNH), 145.1 (C_ipso_), 138.7, 135.9, 134.3, 131.6, 131.3, 131.0, 127.0, 126.9, 124.8, 120.1, 119.0, 118.2 (CN), 114.1 (CN), 101.8 (*C*CONH), 97.5 (*C*CMe), 57.9 (*C*=CNH_2_), 48.0 (C_spiro_), 12.5 (CH_3_); MS (EI, 70 eV): *m*/*z* (%) = 480 (6) [M + 1]^+^, 479 (17) [M]^+^, 453 (7), 413 (100), 384 (12), 271 (57), 246 (35), 208 (59), 188 (54), 163 (22), 139 (15), 111 (76), 75 (38), 50 (11); anal. calcd for C_25_H_14_ClN_7_O_2_: C, 62.57; H, 2.94; N, 20.43. Found: C, 62.8; H, 3.1; N, 20.5.

#### Methyl 6′-amino-4,5′-dicyano-3-oxo-1′-phenyl-2,3-dihydro-1′*H*-spiro[indeno[2,1-*c*]pyridazine-9,4′-pyrano[2,3-*c*]pyrazole]-3′-carboxylate (5h)

White powder; yield: 0.469 g (96%); mp: 290–293 °C; IR (KBr): 3415, 3368, 3321, 3262, 3197, 2233, 2198, 1715, 1651, 1591, 1388, 1169, 938, 763, 695 cm^−1^; ^1^H NMR (300 MHz, DMSO-*d*_6_): *δ* = 13.60 (1H, s, NH), 8.22 (1H, d, *J* = 7.2 Hz, ArH), 7.87 (1H, d, *J* = 7.8 Hz, ArH), 7.47–7.75 (7H, m, ArH), 7.70 (2H, s, NH_2_), 3.38 (3H, s, OCH_3_); ^13^C NMR (75.4 MHz, DMSO-*d*_6_): *δ* = 160.8 (CNH_2_), 159.8 (*C*OOMe), 158.3 (CONH), 156.2 (*C*N-Ph), 153.6 (C=NNH), 148.7 (CCOOMe), 146.4 (*C*C=NNH), 137.2, 136.9, 131.9, 130.5, 130.0, 128.9, 126.8, 126.5, 124.3, 122.6, 118.0 (CN), 114.0 (CN), 100.4 (*C*CONH), 99.8 (*C*CCO_2_Me), 59.1 (*C*=CNH_2_), 52.2 (OCH3), 48.2 (C_spiro_); MS (EI, 70 eV): *m*/*z* (%) = 489 (22) [M]^+^, 463 (10), 423 (79), 395 (43), 318 (13), 271 (33), 215 (39), 188 (40), 99 (8), 77 (100), 51 (22); anal. calcd for C_26_H_15_N_7_O_4_:C, 63.80; H, 3.09; N, 20.03. Found: C, 63.4; H, 3.5; N, 20.2.

#### 2′-Amino-3,5′-dioxo-2,3-dihydro-5′*H*-spiro[indeno[2,1-*c*]pyridazine-9,4′-pyrano[3,2-*c*]chromene]-3′,4-dicarbonitrile (5i)

White powder; yield: 0.385 g (90%); mp: 308–310 °C; IR (KBr): 3440, 3336, 3196, 3099, 3015, 2886, 2233, 3198, 1715, 1661, 1456, 1360, 1170, 1109, 1072, 763, 518 cm^−1^; ^1^H NMR (300 MHz, DMSO-*d*_6_): *δ* = 13.68 (1H, s, NH), 8.21 (1H, d, *J* = 7.5 Hz, ArH), 7.98 (1H, d, *J* = 7.5 Hz, ArH), 7.83 (2H, s, NH_2_), 7.52–7.78 (5H, m, ArH), 7.46 (1H, d, *J* = 8.4 Hz, ArH); ^13^C NMR (75.4 MHz, DMSO-*d*_6_): *δ* = 158.8 (CNH_2_), 158.6 (COO), 158.3 (CONH), 156.1 (C=*C*–O), 155.7 (C=N), 153.1 (*C*–OCO), 152.6 (*C*C=N), 148.2, 135.7, 134.3, 132.0, 130.7, 126.5, 125.5, 124.6, 123.4, 117.2, 112.9, 117.5 (CN), 113.9 (CN), 101.9 (*C*COO), 100.7 (*C*CONHN), 58.0 (*C*=CNH_2_), 47.8 (C_spiro_); MS (EI, 70 eV): *m*/*z* (%) = 433 (12) [M]^+^, 389 (7), 263 (7), 188 (14), 135 (8), 92 (65), 41 (100).

#### 2-Amino-3′,5-dioxo-2′,3′-dihydro-5*H*-spiro[indeno[1,2-*b*]pyran-4,9′-indeno[2,1-*c*]pyridazine]-3,4′-dicarbonitrile (5j)

Yellow powder; yield: 0.383 g (92%); mp: 298–300 °C; IR (KBr): 3440, 3336, 3196, 3099, 3015, 2886, 2233, 3198, 1715, 1661, 1456, 1360, 1170, 1109, 1072, 763, 518 cm^−1^; ^1^H NMR (300 MHz, DMSO-*d*_6_): *δ* = 13.73 (1H, s, NH), 7.27–8.23 (8H, m, ArH), 7.55 (2H, s, NH_2_); ^13^C NMR (75.4 MHz, DMSO-*d*_6_): *δ* = 189.0 (C=O), 167.7 (CNH_2_), 160.8 (C=*C*–O), 158.3 (CONH), 154.7 (C=N), 152.2 (*C*C=N), 147.4, 135.8, 135.4, 134.2, 131.9, 131.5, 130.9, 130.7, 126.8, 124.8, 122.8, 119.6 (Ar), 117.7 (CN), 113.9 (CN), 107.8 (*C*–CO), 101.0 (*C*CONHN), 58.2 (*C*=CNH_2_), 46.5 (C_spiro_).

## Biological experiments

### Determination of antibacterial activity of synthetic compounds by using disk diffusion method

Antibacterial activity of the prepared synthetic compounds against the Gram-negative bacteria (*E. coli*; ATCC: 15922) and the Gram-positive bacteria (*S. aureus*; PTCC: 1112, International no. ATCC6538P) were examined by disk diffusion assay. Bacterial cultures were obtained from Persian Type Culture Collection, Tehran, Iran (PTCC). Isolated pure colonies from fresh grown bacteria were transferred from the plates into sterile normal saline solution and it was vortexed to form bacterial homogenous suspensions. The turbidity was then adjusted to 0.5 McFarland standard units, and the suspensions were poured over Mueller–Hinton agar (MHA) plates. Sterile filter paper disks were placed over these plates. The sterile disks were impregnated with the tested compounds. Positive control (tetracycline) and negative control (sterile distilled water) were used. To determine MIC, the concentrations of tested substance were prepared within the range from 30–5 mM. Incubate in incubator at 37 °C in 24 h for bacteria (Table 2). The MIC results were displayed in Table 3.

### Cell lines and cell culture

Human non-small-cell lung cancer A549 cells, human breast cancer MCF-7 cells, human malignant melanoma cells (A375), human prostate cancer cells (PC3 cells, LNCaP cells) and normal cells HDF (human dermal fibroblast) were received from Pasture Institute, Tehran, Iran. MCF-7 cells were grown in RPMI 1640 medium, A549 cells, A375 cells, PC3 cells, LNCaP cells and HDF cells were grown in DMEM medium. All media contain 10% fetal bovine serum (FBS), penicillin G, streptomycin 100 μg mL^−1^ and 1% l-glutamine. The cells were cultured and incubated under humidified 5% CO_2_ atmosphere at 37 °C.

### MTT assay

The effect of compounds treatment on the viability of cancer cell lines was measured by (3-(4,5-dimethylthiazol-2-yl)-2,5-diphenyl tetrazolium bromide or MTT) assay (MTT assay kit, Bio IDEA, CatNo: BI1017, Iran) based on the ability of live cells to cleave the tetrazolium ring to a molecule that absorb at 4900 nm as per the manufacturer's instructions.^33^ Etoposide (was kindly provided by Dr A. Foroumadi, Tehran medical Science University, Iran) and dimethyl sulfoxide (or DMSO) was used as positive and negative controls, respectively.^34^ Briefly, cells were plated in 96-well culture plates (5 × 10^3^ cells per well). After 24 h incubation, the cells were treated with different concentrations of the compounds. After 48 h at 37 °C, the medium was removed and 100 μL of MTT reagent (1 mg mL^−1^) was added to each well, and cells were further incubated at 37 °C for 4 h. The MTT solution was removed, 50 μL of DMSO was added to each well to dissolve formazan crystals, and the plates were gently shaken for 10 minutes, followed by reading with an ELISA plate reader (BiotekELx 800, USA). The 50% inhibition concentration (IC_50_) was defined as the concentration that inhibited cell proliferation by 50% when compared to DMSO treated cells (as negative control).

### DAPI staining assay

DAPI staining assay was used to determine chromatin changes. A549 cells were seeded in six well plates (5 × 10^4^ cells per well) containing 12 mm cover-slips and subsequently treated for compound 5a (sample or treated cells) and DMSO (control or untreated cells) for 24 h. Cells then were fixed with 3.7% paraformaldehyde, permeabilized in 0.5% (w/v) Triton X-100, 1% BSA (w/v) for 5 min, washed in PBS, and stained with DAPI (Sigma-Aldrich, USA). All images were taken by an inverted fluorescent microscope (Nikon Eclipse Ti-E).

### RNA extraction, CDNA synthesis and real-time PCR (RT-PCR)

For quantitative real-time RT-PCR analysis, after 48 h of treatment with 40 μM of related drug (5a), A549 cells was lysed and the total RNA was extracted using 500 μL of Trizol® reagent according to the protocol provided by the manufacturer (Invitrogen Life Technologies, Carlsbad, CA, USA) followed by reverse transcription into cDNA according to manufactures protocol (ReveretAid M-Mulv reverse transcriptase kit, Thermo Fisher Scientific, MA, USA). Real-time RT-PCR was then performed to amplify cDNA using SYBR green dye universal master mix (Bioron GmbH, Germany), on a Light Cycler 480 (Roche) using the primers for GAPDH-F: 5′-CAA GGT CAT CCA TGA CAA CTTTG-3′, R:5′-GTCCACCACCCTGTTGCTGTAG-3′; Bax-F:5′-GTCGCCCTTTTCTACTTTGCC-3′, R: 5′-CTCCCGCCACAAAGATGGTCA-3′and Bcl-2-F: 5′-CCCCTCGTCCAAGAATGCAA-3′, R: 5′- TCTCCCGGTTATCGTACCCTG-3′for forty cycles. Data represent averaged copy number normalized to the GAPDH housekeeping gene. Primer synthesis was done by Pishgam Biotech Co. Tehran, Iran. The negative control reaction was set as a reaction similar to the above but with deionized water instead of cDNA. Thermal conditions of the PCR consisted of primary denaturation at 94 °C for 2 minutes, 45 cycles of denaturation at 94 °C for 30 seconds, annealing at 59 °C for 30 seconds, amplification at 72 °C for 30 seconds. All reactions were triplicated.

### Statistical analysis

Data were analyzed using SPSS (ver. 22, Chicago, IL, USA) and graphs were generated using GraphPad Prism 7 software. Data were expressed as means ± standard deviation (SD). Experiments were performed in triplicate. Comparisons between groups were performed using independent sample *t*-test. As value of *p* < 0.05 was considered to be statistically significant.

## Conclusions

In this work, a series of novel fused spiro-4*H*-pyrans was synthesized through a one-pot four-component reaction between cyanoacetohydrazide, ninhydrin, malononitrile and cyclic CH-acids. These derivatives were examined *in vitro* for activity against *Escherichia coli* (*E. coli*) and *Staphylococcus aureus* (*S. aureus*). Antibacterial test by using agar dilution method showed that *S. aureus* was sensitive to 5a, 5b, 5c, 5e, 5f, 5g and 5i and *E. coli* was resistant to all compounds. In addition, the anticancer effects of synthetic compounds were evaluated against several cancer cell lines. Compound 5a showed the most anticancer activity on A549 and LNCaP cancer cells with IC_50_ value lower than Etoposide. It was found that compound 5a can induce apoptotic mediated mitochondria pathway *via* down-regulation of Bcl-2 and up-regulation of Bax. Overall, it seems that derivative 5a may be possible candidate to design drugs for cancer treatment strategies and infection diseases. The results demonstrated that our compounds have no cell viability effects on normal cell line, HDF, and two cancer cell lines, MCF-7 and PC3. It was demonstrated that the resistance of cancer cells to chemotherapeutic agents to be multifactorial and to be associated with mechanisms related to drug transport, drug inactivation, DNA damage response, DNA repair and the modulation of apoptosis and thereby, more experiments will be required to explain our results.^35^ It means that depending on cell context and complexity of network interactions, a variety of alternative strategies provides or inhibits resistance of cancer cells to chemotherapeutic drugs. More detailed studies will be required to be performed in order to better understand why some cancer cells were resistant to our synthetic compounds and some of them were susceptible.

## Conflicts of interest

There are no conflicts to declare.

## Supplementary Material

RA-009-C9RA03196K-s001
