# Supplementary material for: Synthesis and evaluation of antimicrobial activity, cytotoxic and pro-apoptotic effects of novel spiro-4H-pyran derivatives
Source: RSC Adv. 2019 Aug 9;9(43):24843–51. doi: 10.1039/c9ra03196k (PMC9070039; doi:10.1039/c9ra03196k)
Supplement: RA-009-C9RA03196K-s001 [file RA-009-C9RA03196K-s001.pdf]

Electronic Supplementary Material (ESI) for RSC Advances.
This journal is © The Royal Society of Chemistry 2019

Supporting Information

Synthesis and evaluation of cytotoxic activity and pro-apoptotic effects of novel spiro-4*H*-pyran derivatives on A549 cancer cells

Fatemeh Safari,* Hajar Hosseini, Mohammad Bayat* and Ashkan Ranjbar

Chemistry Department, Imam Khomeini International University, Qazvin, Iran

(Phone: +98 281 33780040; fax: +98 281 33780040; e-mail: bayat_mo@yahoo.com, m.bayat@sci.ikiu.ac.ir)

The Table of Contents

Title	Page
Title, author's name, address and table of contents	1
Experimental Section; General remarks	2
**Figure 1.** Structure of all products **5**	3
^{1}H and ^{13}C NMR and IR and Mass spectrums of **5a**	4-7
^{1}H and ^{13}C NMR and Mass spectrums of **5b**	8-10
^{1}H and ^{13}C NMR and Mass spectrums of **5c**	11-13
^{1}H and ^{13}C NMR and IR and Mass and D_2O exchange spectrums of **5d**	14-17
^{1}H and ^{13}C NMR and IR and Mass spectrums of **5e**	18-21
^{1}H and ^{13}C NMR and IR and Mass spectrums of **5f**	22-25
^{1}H and ^{13}C NMR and IR and Mass spectrums of **5g**	26-29
^{1}H and ^{13}C NMR and IR and Mass spectrums of **5h**	30-33
^{1}H and ^{13}C NMR and IR and Mass spectrums of **5i**	34-37
^{1}H and ^{13}C NMR and IR spectrums of **5j**	38-40

Experimental Section

General remarks:

Melting points were measured on an Electrothermal 9100 apparatus. Mass spectra were recorded with an Agilent 5975C VL MSD with Triple-Axis Detector operating at an ionization potential of 70 eV. ^{1}H and ^{13}C NMR spectra were measured (DMSO) with a Bruker DRX-300 AVANCE spectrometer at 300 and 75 MHz, respectively. IR spectra were recorded on a Bruker Tensor 27, ῡ in cm^{-1}. All NMR spectra at room temperature were determined in DMSO-d_6. Chemical shifts are reported in parts per million (δ) downfield from an internal tetramethylsilane reference. Coupling constants (J values) are reported in Hertz (Hz), and spin multiplicities are indicated by the following symbols: s (singlet), d (doublet), t (triplet), q (quartet), m (multiplet). All chemicals were purchased from Merck or Aldrich and were used without further purification.

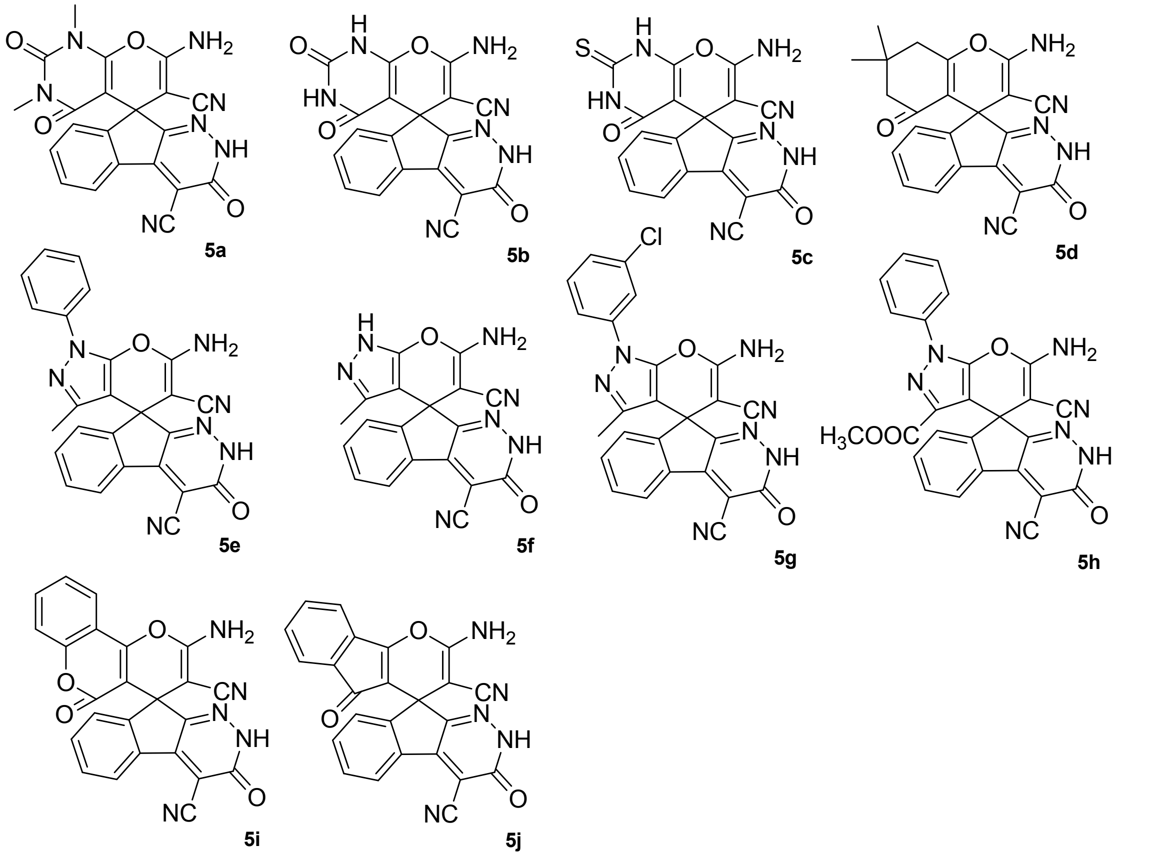

Figure 1. Structure of all products **5**

The structures of all products **5a-j** were deduced from their ^{1}H NMR, and ^{13}C NMR, IR and Mass spectra (see the Supporting Information)

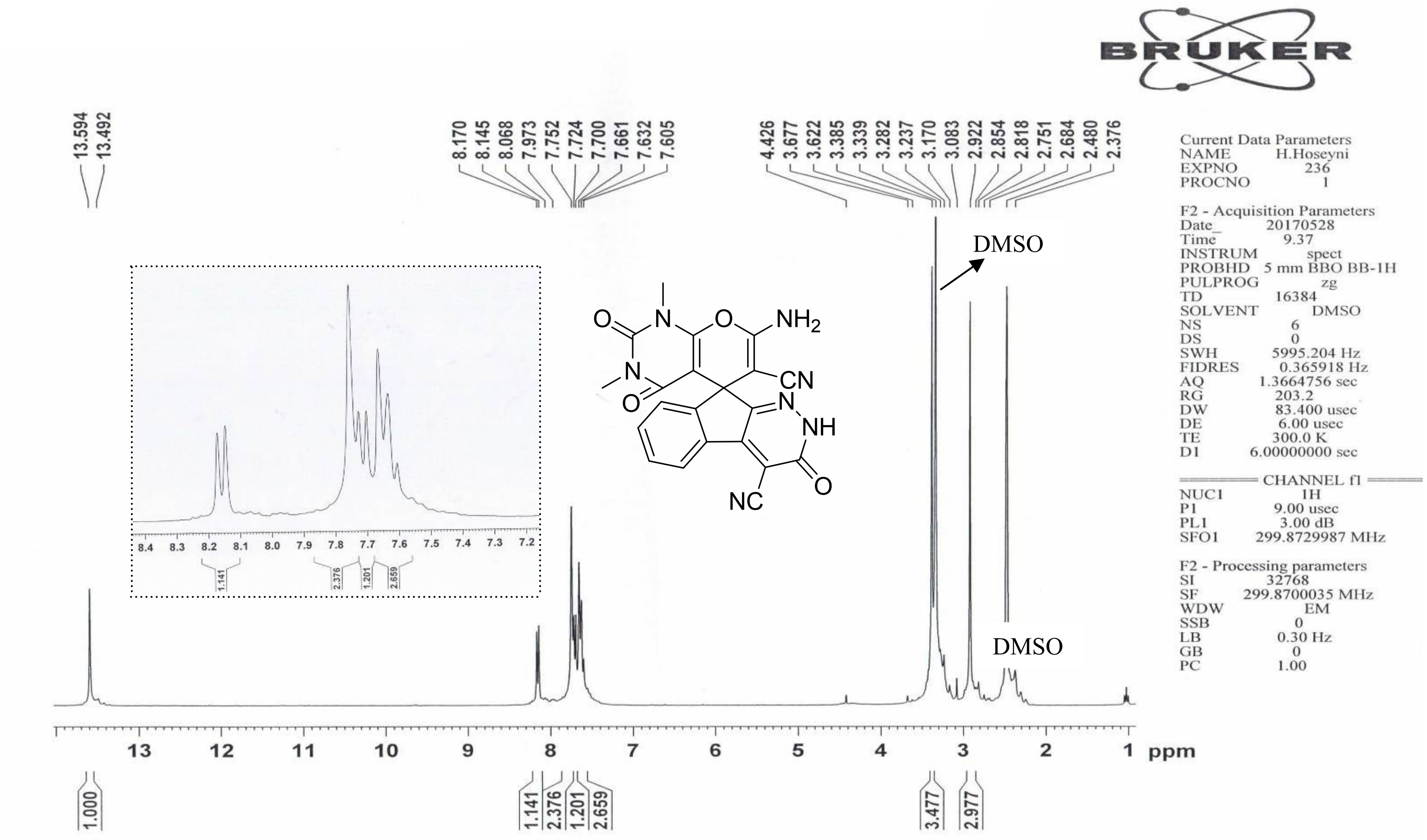
BRUKER
13.594
13.492
8.170
8.145
8.068
7.973
7.752
7.724
7.700
7.661
7.632
7.605
4.426
3.677
3.622
3.385
3.339
3.282
3.237
3.170
3.083
2.922
2.854
2.818
2.751
2.684
2.480
2.376
DMSO
DMSO
O
N
O
NH2
N
CN
O
N
NH
O
NC
Current Data Parameters
NAME H.Hoseyni
EXPNO 236
PROCNO 1
F2 - Acquisition Parameters
Date_ 20170528
Time 9.37
INSTRUM spect
PROBHD 5 mm BBO BB-1H
PULPROG zg
TD 16384
SOLVENT DMSO
NS 6
DS 0
SWH 5995.204 Hz
FIDRES 0.365918 Hz
AQ 1.3664756 sec
RG 203.2
DW 83.400 usec
DE 6.00 usec
TE 300.0 K
D1 6.00000000 sec
CHANNEL f1
NUC1 1H
P1 9.00 usec
PL1 3.00 dB
SFO1 299.8729987 MHz
F2 - Processing parameters
SI 32768
SF 299.8700035 MHz
WDW EM
SSB 0
LB 0.30 Hz
GB 0
PC 1.00
8.4 8.3 8.2 8.1 8.0 7.9 7.8 7.7 7.6 7.5 7.4 7.3 7.2
1.141 2.376 1.201 2.659
13 12 11 10 9 8 7 6 5 4 3 2 1 ppm
1.000
1.141
2.376
1.201
2.659
3.477
2.977

^{1}H NMR of 5a

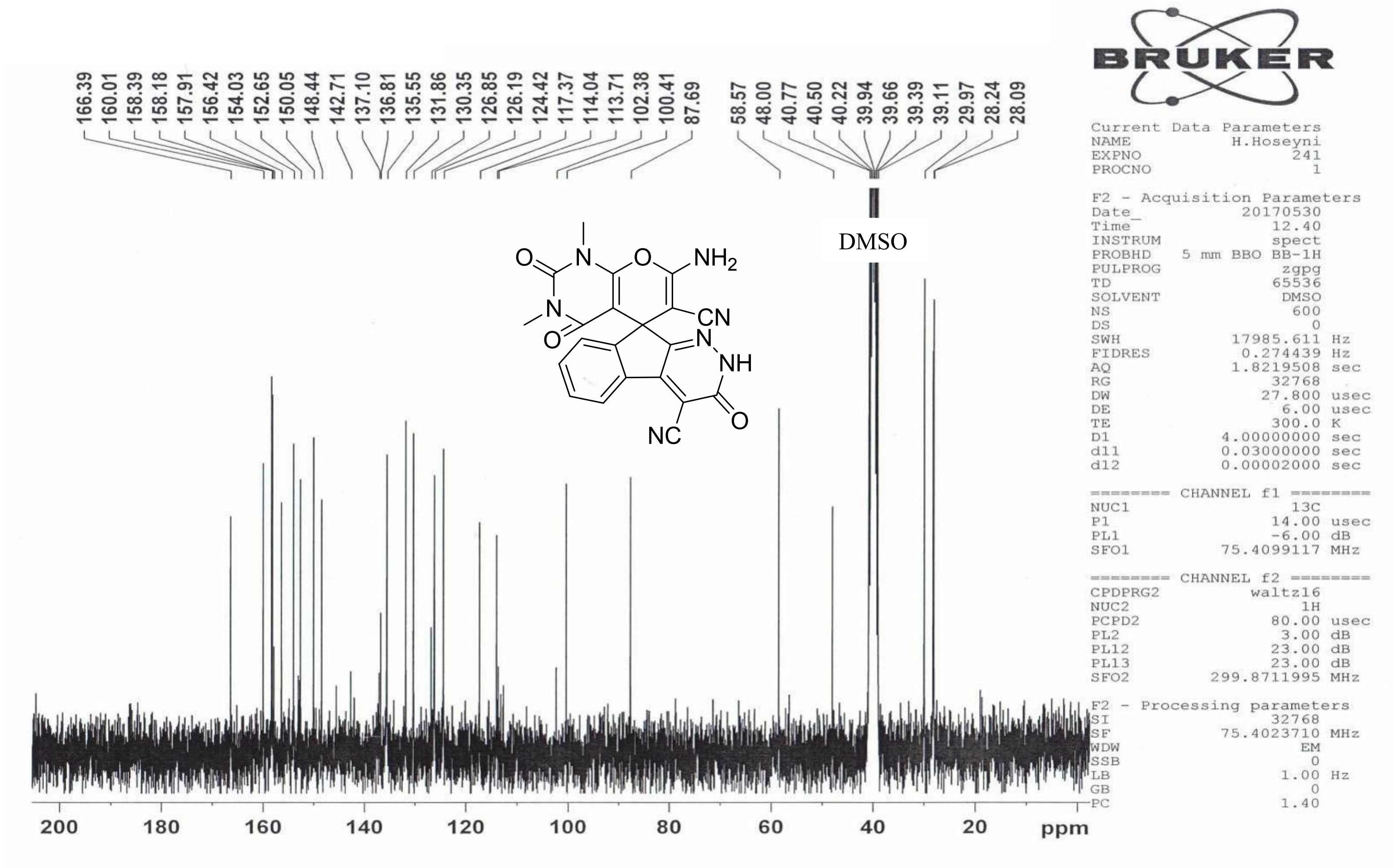
166.39
160.01
158.39
158.18
157.91
156.42
154.03
152.65
150.05
148.44
142.71
137.10
136.81
135.55
131.86
130.35
126.85
126.19
124.42
117.37
114.04
113.71
102.38
100.41
87.69
58.57
48.00
40.77
40.50
40.22
39.94
39.66
39.39
39.11
29.97
28.24
28.09
DMSO
O
N
O
NH2
CN
NH
NC
200 180 160 140 120 100 80 60 40 20 ppm
BRUKER
Current Data Parameters
NAME H.Hoseyni
EXPNO 241
PROCNO 1
F2 - Acquisition Parameters
Date_ 20170530
Time 12.40
INSTRUM spect
PROBHD 5 mm BBO BB-1H
PULPROG zgpg
TD 65536
SOLVENT DMSO
NS 600
DS 0
SWH 17985.611 Hz
FIDRES 0.274439 Hz
AQ 1.8219508 sec
RG 32768
DW 27.800 usec
DE 6.00 usec
TE 300.0 K
D1 4.00000000 sec
d11 0.03000000 sec
d12 0.00002000 sec
======== CHANNEL f1 ========
NUC1 13C
P1 14.00 usec
PL1 -6.00 dB
SFO1 75.4099117 MHz
======== CHANNEL f2 ========
CPDPRG2 waltz16
NUC2 1H
PCPD2 80.00 usec
PL2 3.00 dB
PL12 23.00 dB
PL13 23.00 dB
SFO2 299.8711995 MHz
F2 - Processing parameters
SI 32768
SF 75.4023710 MHz
WDW EM
SSB 0
LB 1.00 Hz
GB 0
PC 1.40

^{13}C NMR of 5a

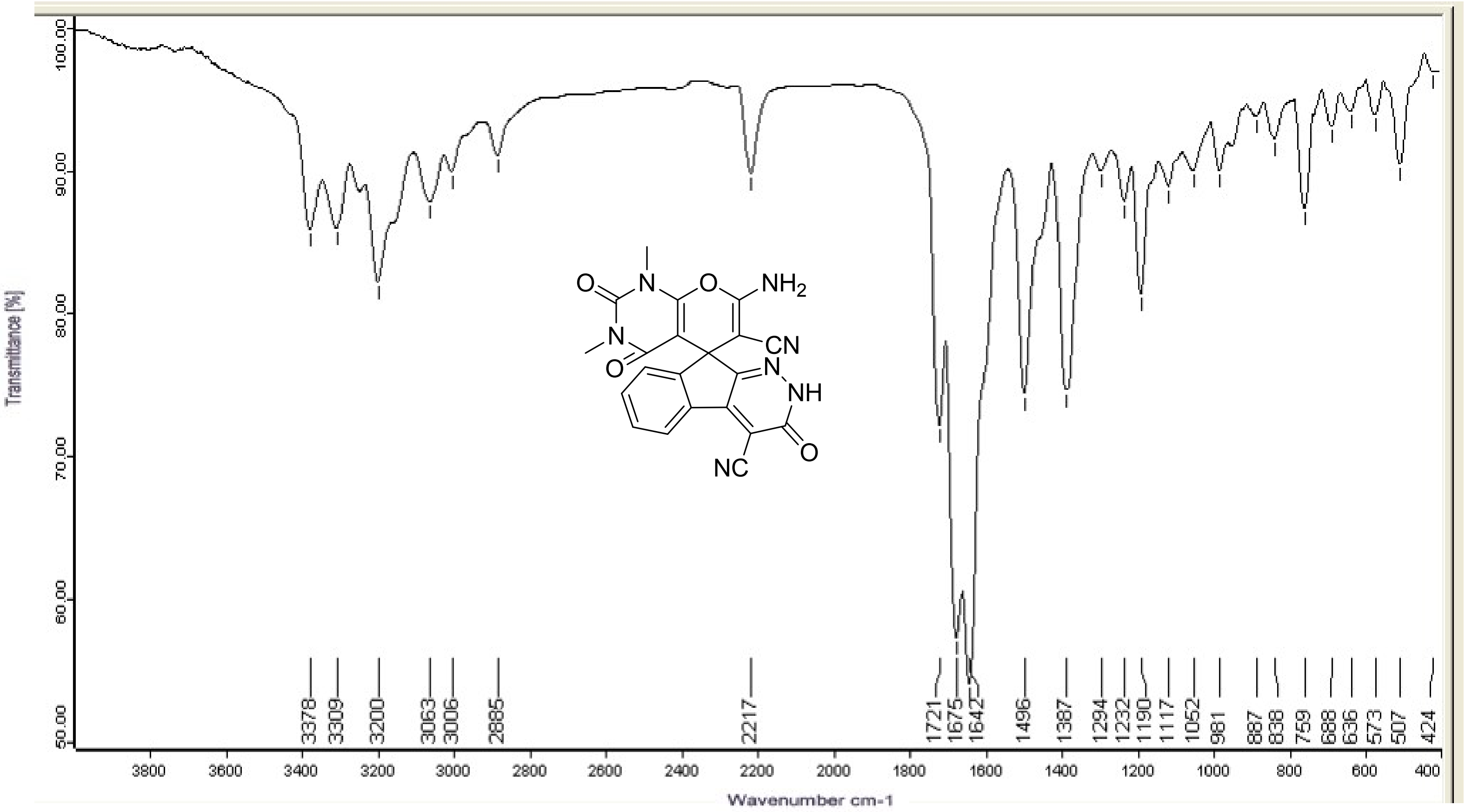
Transmittance [%]
100.00
90.00
80.00
70.00
60.00
50.00
Wavenumber cm-1
3800
3600
3400
3200
3000
2800
2600
2400
2200
2000
1800
1600
1400
1200
1000
800
600
400
3378
3309
3200
3063
3006
2885
2217
1721
1675
1642
1496
1387
1294
1232
1190
1117
1052
981
887
838
759
688
636
573
507
424
O
N
O
NH2
N
CN
O
N
NH
NC
O

IR of 5a

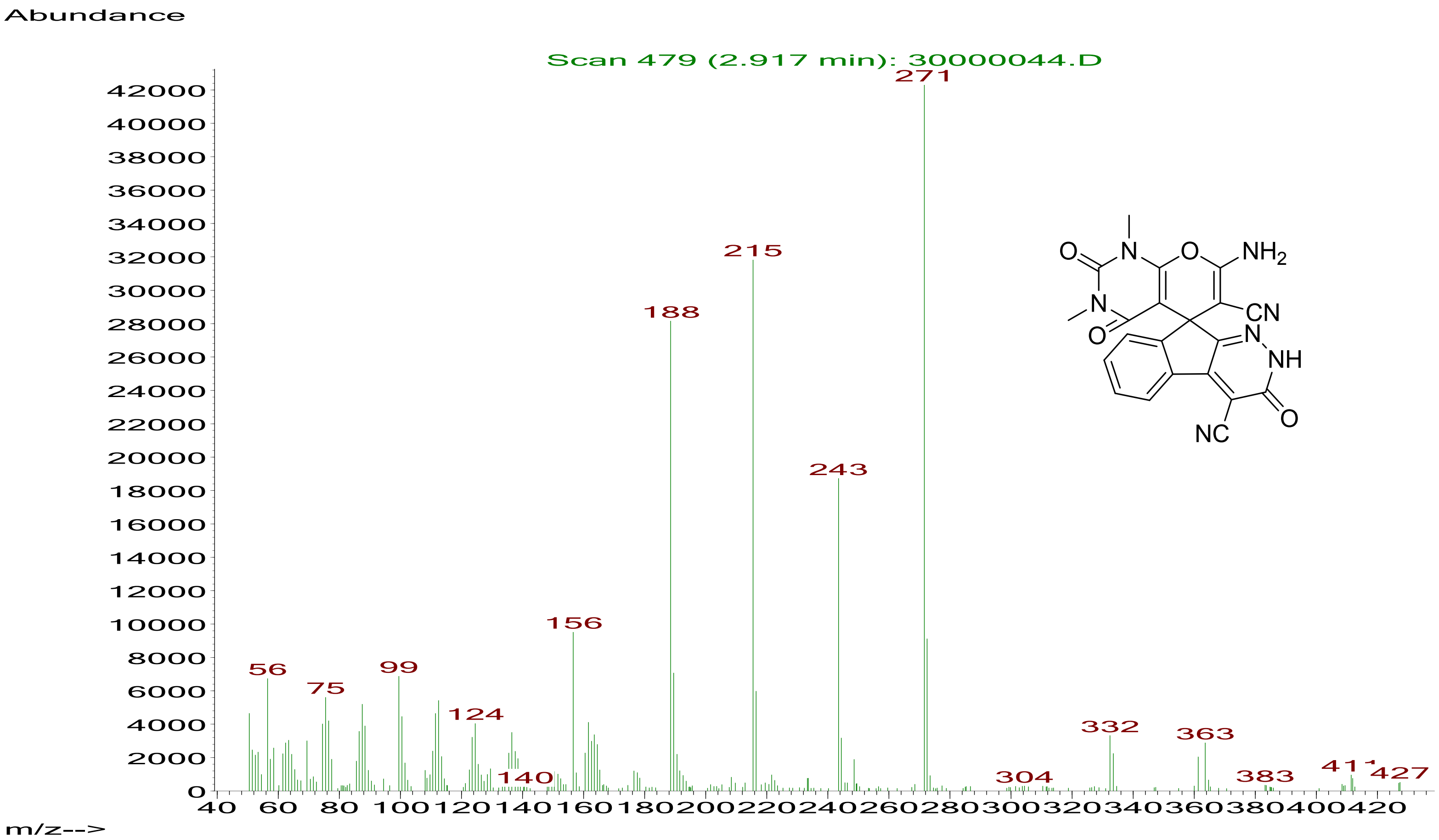

Abundance
Scan 479 (2.917 min): 30000044.D
42000
40000
38000
36000
34000
32000
30000
28000
26000
24000
22000
20000
18000
16000
14000
12000
10000
8000
6000
4000
2000
0
271
215
188
243
156
56
75
99
124
140
304
332
363
383
427
m/z-->
40 60 80 100 120 140 160 180 200 220 240 260 280 300 320 340 360 380 400 420
O
N
O
NH2
N
CN
N
NH
O
NC
O

MS of 5a

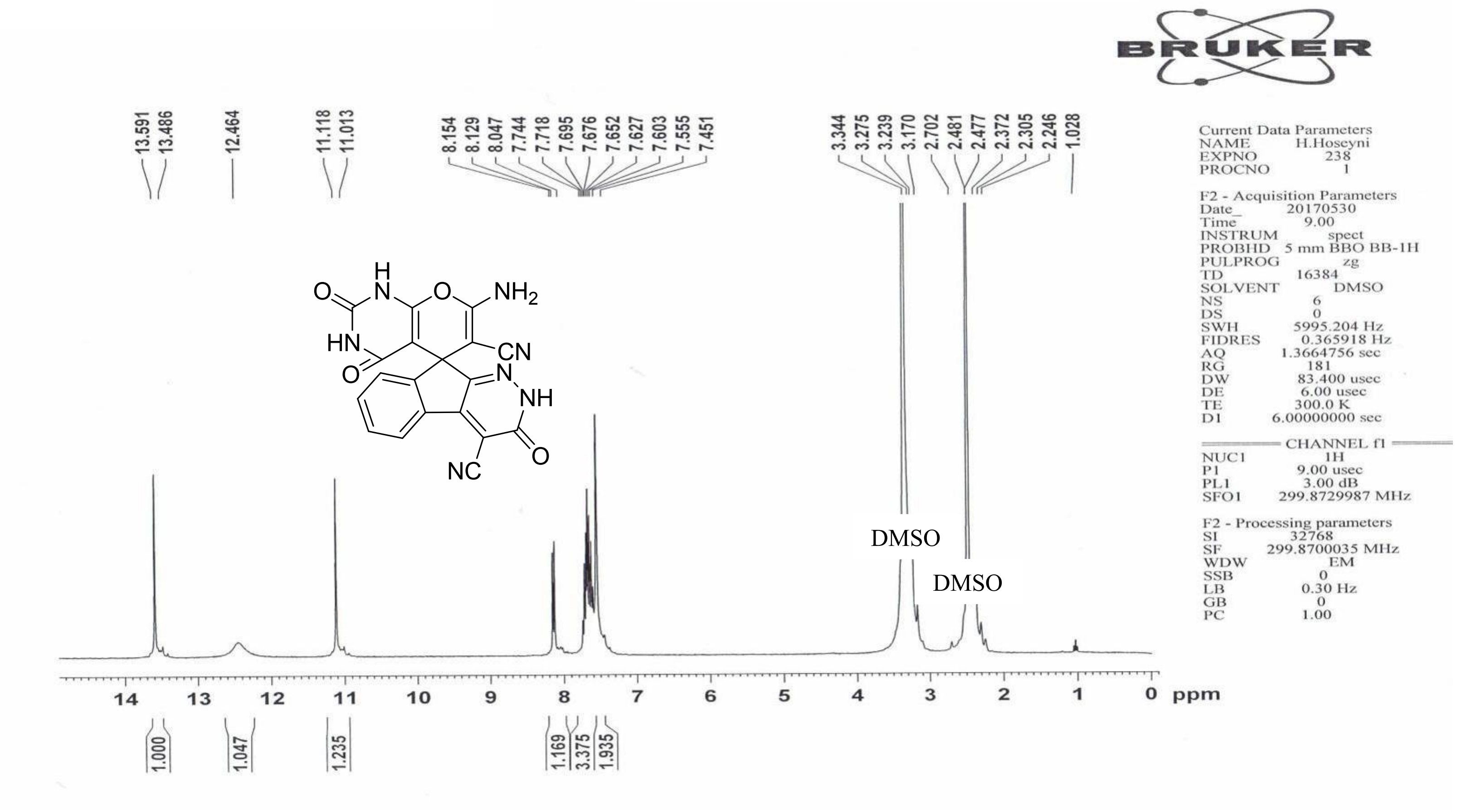
BRUKER
13.591
13.486
12.464
11.118
11.013
8.154
8.129
8.047
7.744
7.718
7.695
7.676
7.652
7.627
7.603
7.555
7.451
3.344
3.275
3.239
3.170
2.702
2.481
2.477
2.372
2.305
2.246
1.028
O
N
H
O
NH2
HN
CN
O
N
NH
NC
O
DMSO
DMSO
14 13 12 11 10 9 8 7 6 5 4 3 2 1 0 ppm
1.000
1.047
1.235
1.169
3.375
1.935
Current Data Parameters
NAME H.Hoseyni
EXPNO 238
PROCNO 1
F2 - Acquisition Parameters
Date_ 20170530
Time 9.00
INSTRUM spect
PROBHD 5 mm BBO BB-1H
PULPROG zg
TD 16384
SOLVENT DMSO
NS 6
DS 0
SWH 5995.204 Hz
FIDRES 0.365918 Hz
AQ 1.3664756 sec
RG 181
DW 83.400 usec
DE 6.00 usec
TE 300.0 K
D1 6.00000000 sec
CHANNEL f1
NUC1 1H
P1 9.00 usec
PL1 3.00 dB
SFO1 299.8729987 MHz
F2 - Processing parameters
SI 32768
SF 299.8700035 MHz
WDW EM
SSB 0
LB 0.30 Hz
GB 0
PC 1.00

^{1}H NMR of 5b

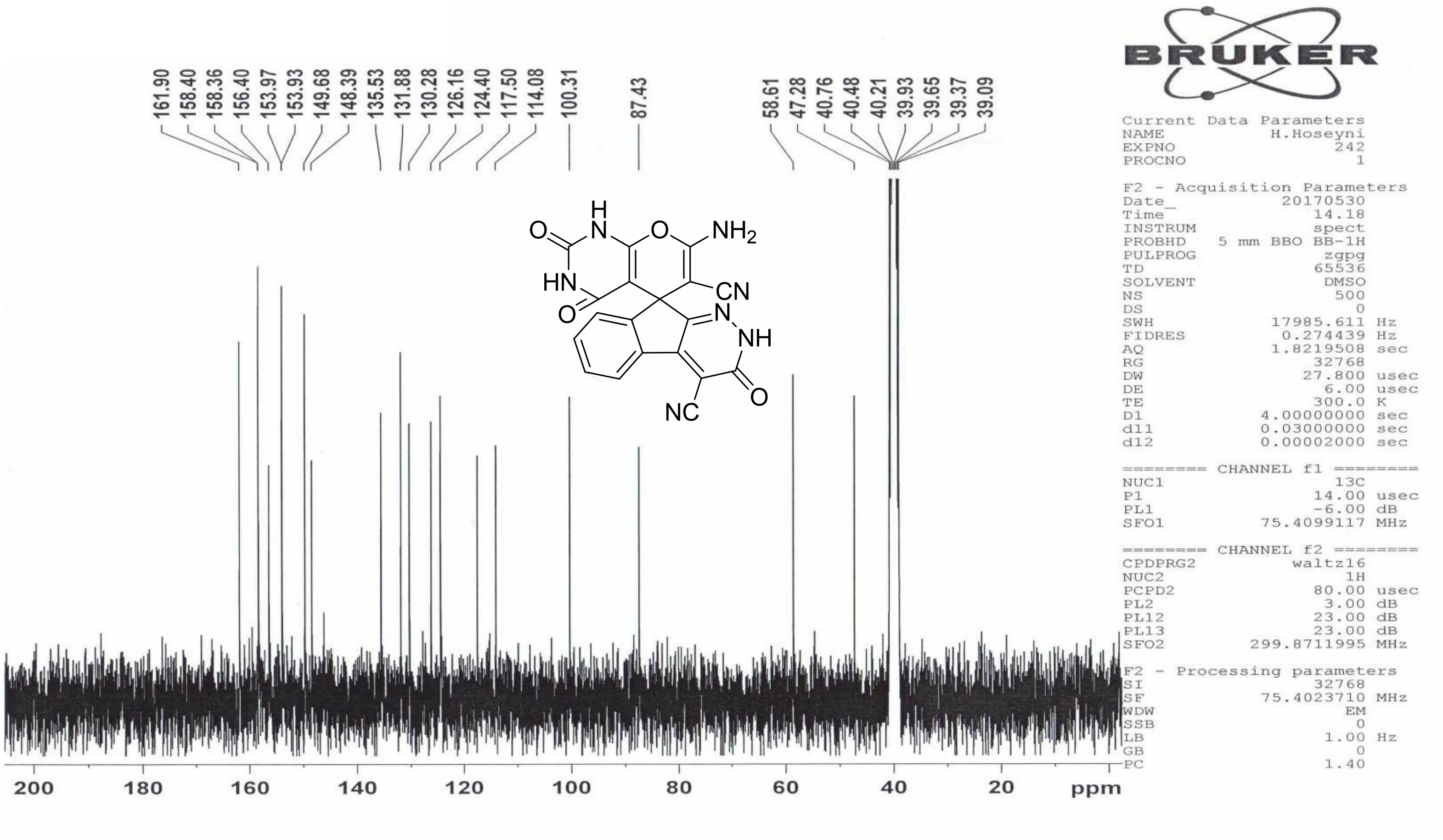
161.90
158.40
158.36
156.40
153.97
153.93
149.68
148.39
135.53
131.88
130.28
126.16
124.40
117.50
114.08
100.31
87.43
58.61
47.28
40.76
40.48
40.21
39.93
39.65
39.37
39.09
NH2
CN
NC
200 180 160 140 120 100 80 60 40 20 ppm
BRUKER
Current Data Parameters
NAME H.Hoseyni
EXPNO 242
PROCNO 1
F2 - Acquisition Parameters
Date_ 20170530
Time 14.18
INSTRUM spect
PROBHD 5 mm BBO BB-1H
PULPROG zgpg
TD 65536
SOLVENT DMSO
NS 500
DS 0
SWH 17985.611 Hz
FIDRES 0.274439 Hz
AQ 1.8219508 sec
RG 32768
DW 27.800 usec
DE 6.00 usec
TE 300.0 K
D1 4.00000000 sec
d11 0.03000000 sec
d12 0.00002000 sec
======== CHANNEL f1 ========
NUC1 13C
P1 14.00 usec
PL1 -6.00 dB
SFO1 75.4099117 MHz
======== CHANNEL f2 ========
CPDPRG2 waltz16
NUC2 1H
PCPD2 80.00 usec
PL2 3.00 dB
PL12 23.00 dB
PL13 23.00 dB
SFO2 299.8711995 MHz
F2 - Processing parameters
SI 32768
SF 75.4023710 MHz
WDW EM
SSB 0
LB 1.00 Hz
GB 0
PC 1.40

^{13}C NMR of 5b

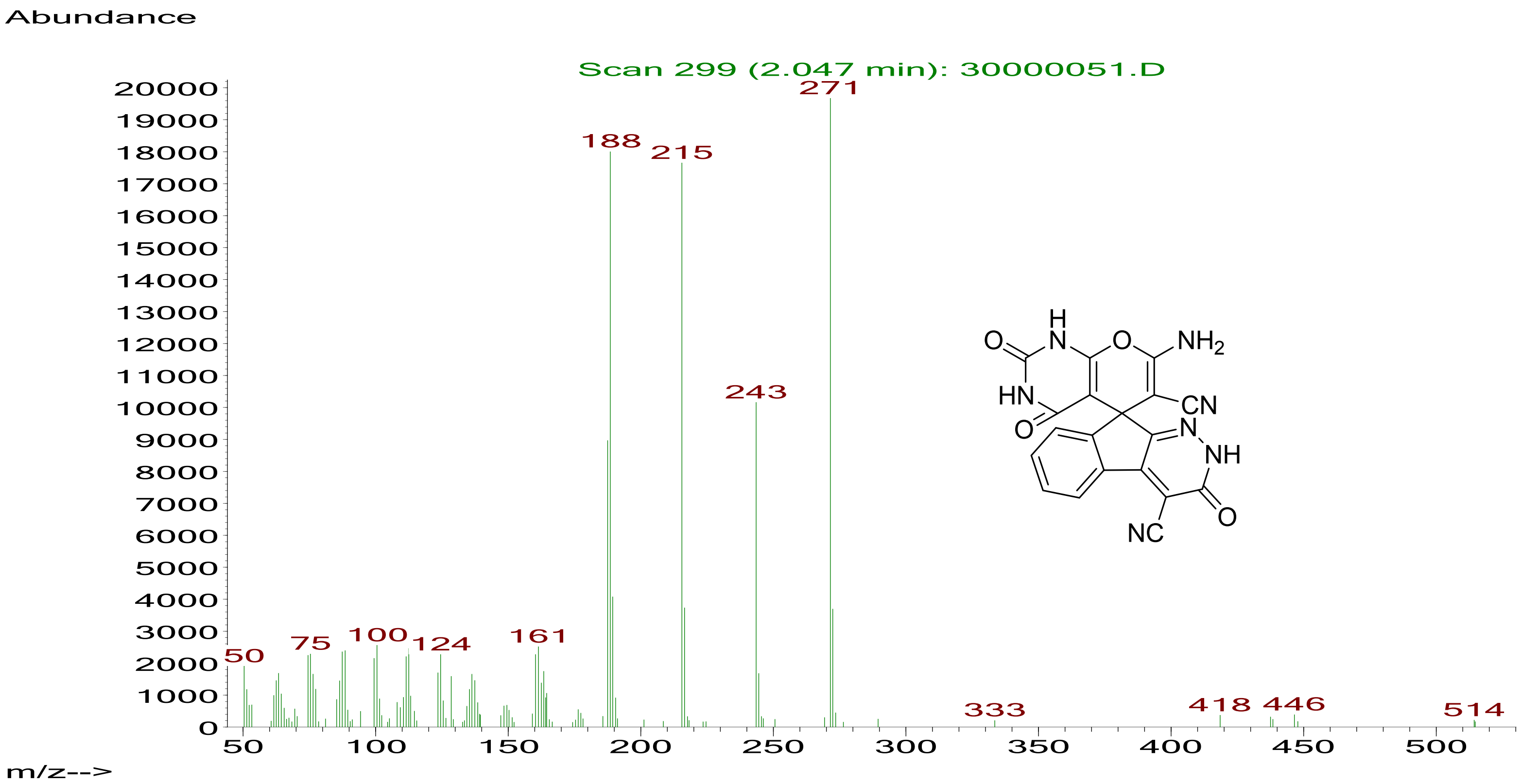
Abundance
Scan 299 (2.047 min): 30000051.D
20000
19000
18000
17000
16000
15000
14000
13000
12000
11000
10000
9000
8000
7000
6000
5000
4000
3000
2000
1000
0
271
188
215
243
50
75
100
124
161
333
418
446
514
50
100
150
200
250
300
350
400
450
500
m/z-->
H
N
O
NH2
O
HN
CN
O
N
NH
NC
O

MS of 5b

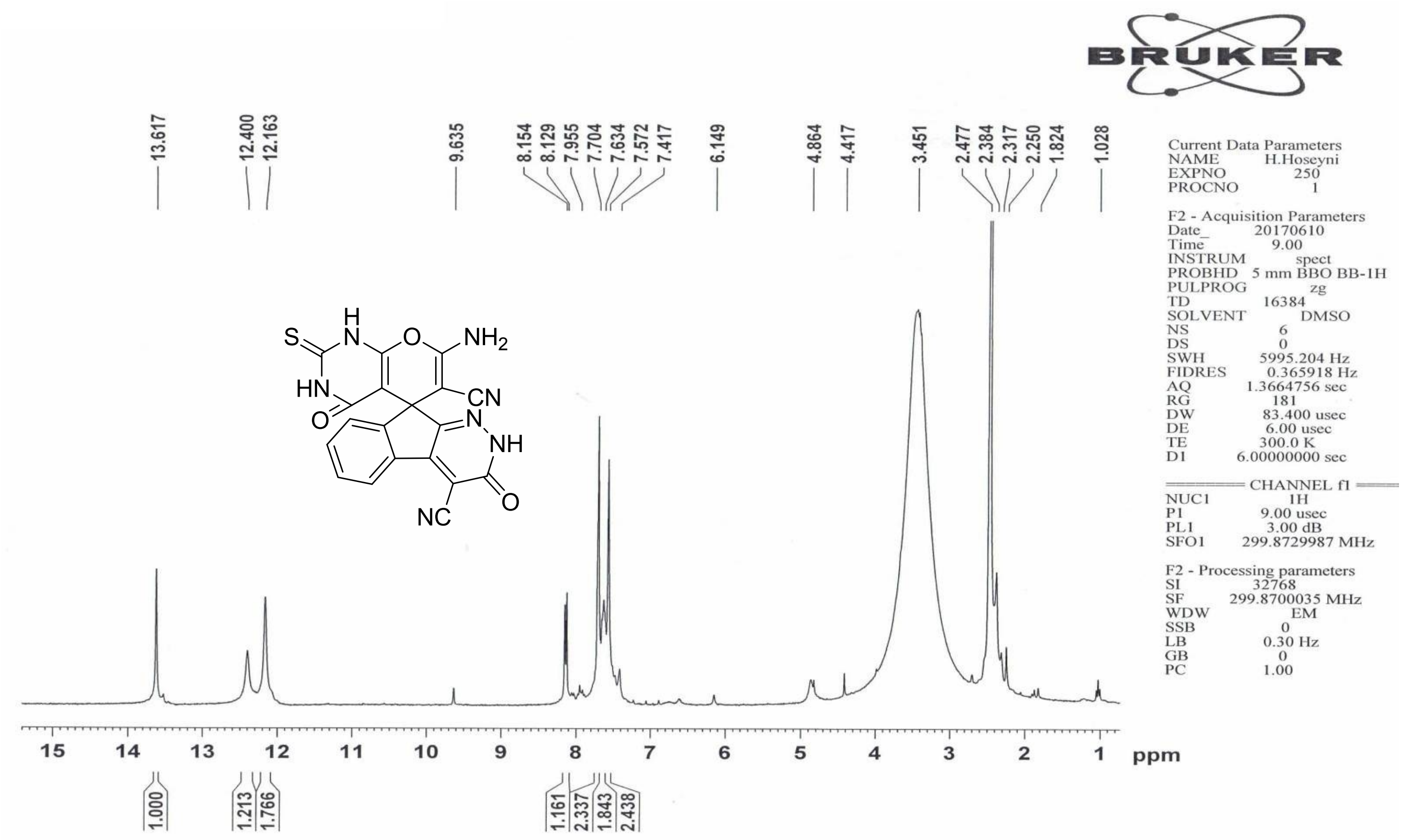

BRUKER
13.617
12.400
12.163
9.635
8.154
8.129
7.955
7.704
7.634
7.572
7.417
6.149
4.864
4.417
3.451
2.477
2.384
2.317
2.250
1.824
1.028
Current Data Parameters
NAME H.Hoseyni
EXPNO 250
PROCNO 1
F2 - Acquisition Parameters
Date_ 20170610
Time 9.00
INSTRUM spect
PROBHD 5 mm BBO BB-1H
PULPROG zg
TD 16384
SOLVENT DMSO
NS 6
DS 0
SWH 5995.204 Hz
FIDRES 0.365918 Hz
AQ 1.3664756 sec
RG 181
DW 83.400 usec
DE 6.00 usec
TE 300.0 K
D1 6.00000000 sec
CHANNEL f1
NUC1 1H
P1 9.00 usec
PL1 3.00 dB
SFO1 299.8729987 MHz
F2 - Processing parameters
SI 32768
SF 299.8700035 MHz
WDW EM
SSB 0
LB 0.30 Hz
GB 0
PC 1.00
ppm
1.000
1.213
1.766
1.161
2.337
1.843
2.438

^{1}H NMR of 5c

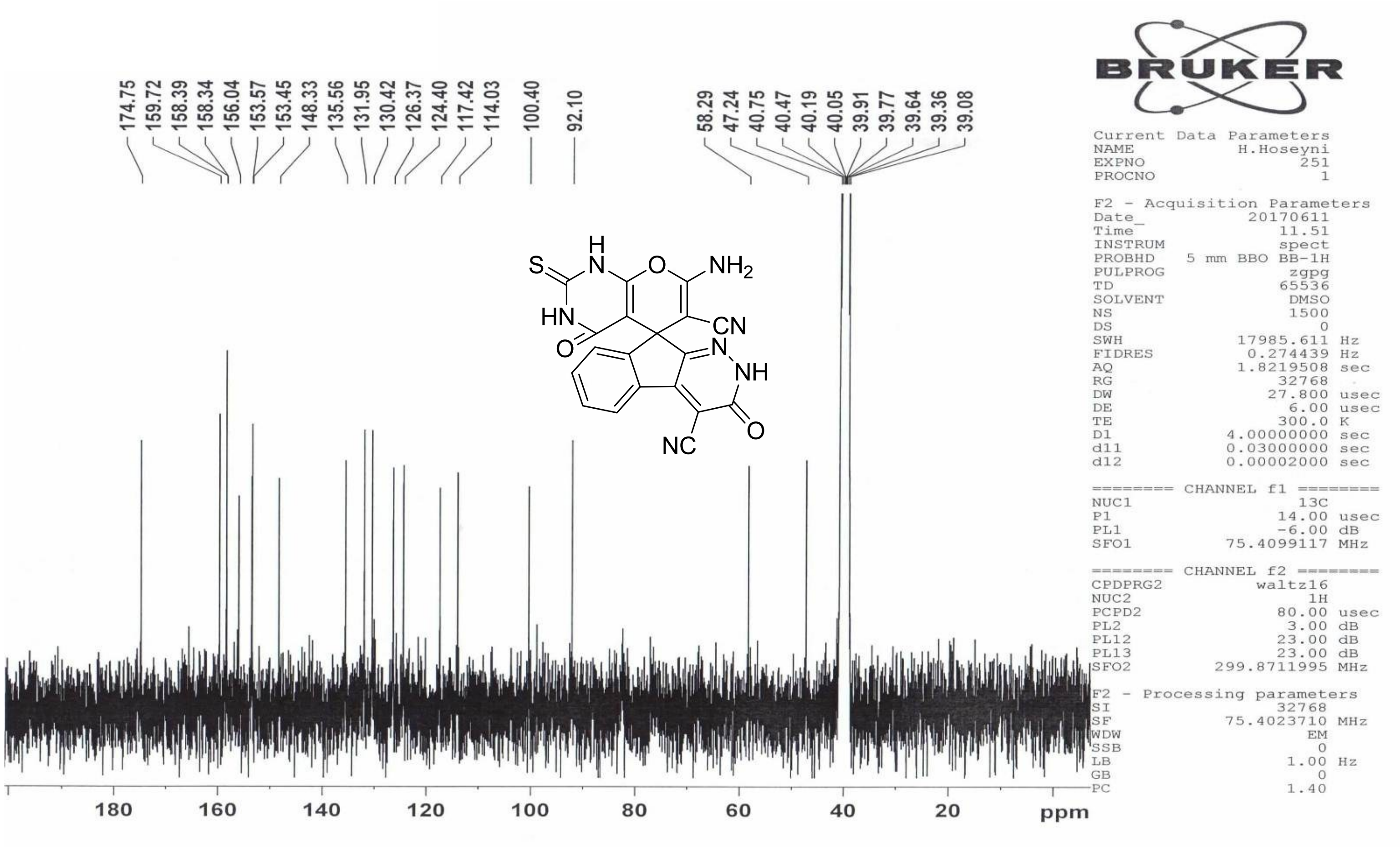
174.75
159.72
158.39
158.34
156.04
153.57
153.45
148.33
135.56
131.95
130.42
126.37
124.40
117.42
114.03
100.40
92.10
58.29
47.24
40.75
40.47
40.19
40.05
39.91
39.77
39.64
39.36
39.08
BRUKER
Current Data Parameters
NAME H.Hoseyni
EXPNO 251
PROCNO 1
F2 - Acquisition Parameters
Date_ 20170611
Time 11.51
INSTRUM spect
PROBHD 5 mm BBO BB-1H
PULPROG zgpg
TD 65536
SOLVENT DMSO
NS 1500
DS 0
SWH 17985.611 Hz
FIDRES 0.274439 Hz
AQ 1.8219508 sec
RG 32768
DW 27.800 usec
DE 6.00 usec
TE 300.0 K
D1 4.00000000 sec
d11 0.03000000 sec
d12 0.00002000 sec
======== CHANNEL f1 ========
NUC1 13C
P1 14.00 usec
PL1 -6.00 dB
SFO1 75.4099117 MHz
======== CHANNEL f2 ========
CPDPRG2 waltz16
NUC2 1H
PCPD2 80.00 usec
PL2 3.00 dB
PL12 23.00 dB
PL13 23.00 dB
SFO2 299.8711995 MHz
F2 - Processing parameters
SI 32768
SF 75.4023710 MHz
WDW EM
SSB 0
LB 1.00 Hz
GB 0
PC 1.40
180 160 140 120 100 80 60 40 20 ppm

^{13}C NMR of 5c

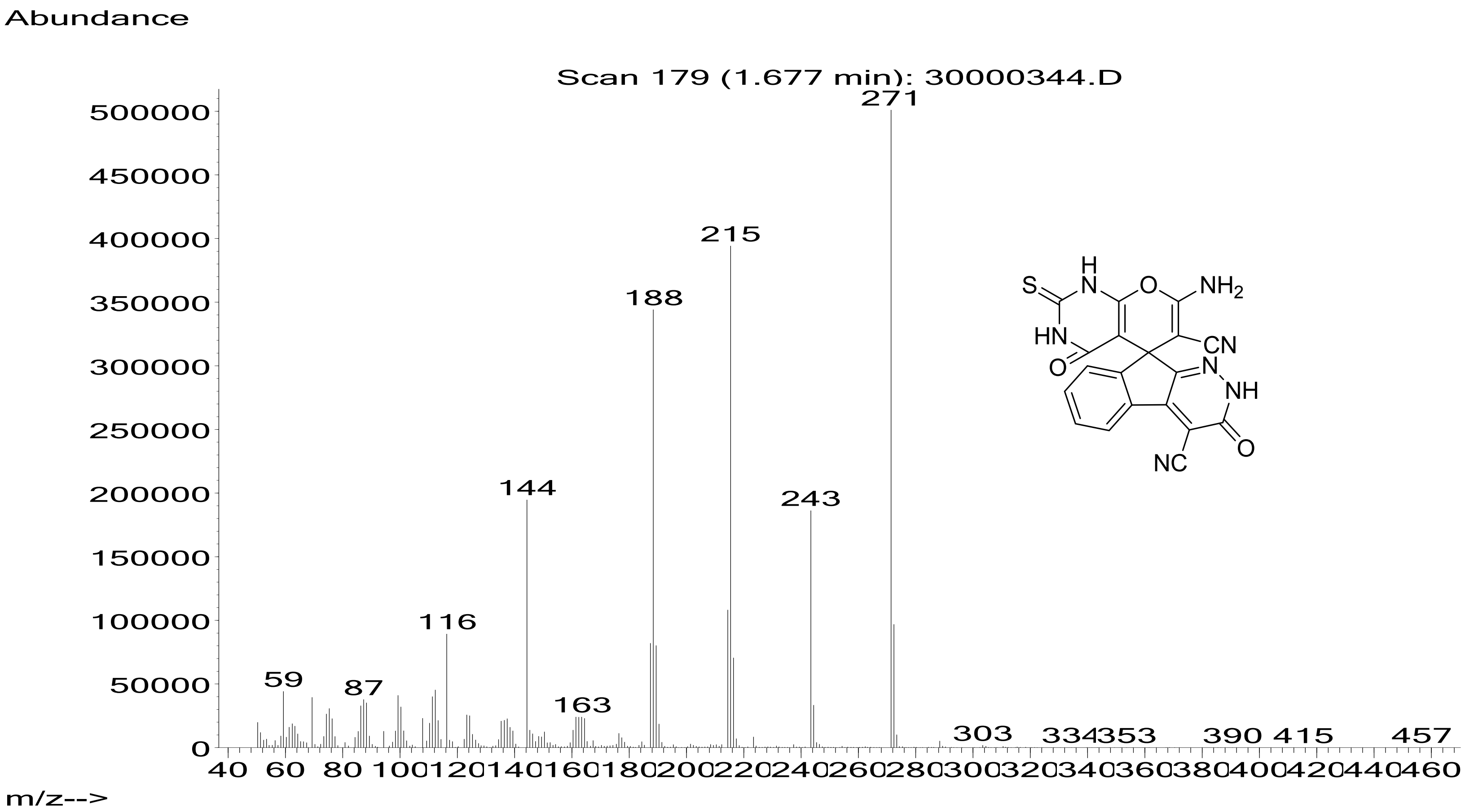
Abundance
Scan 179 (1.677 min): 30000344.D
500000
450000
400000
350000
300000
250000
200000
150000
100000
50000
0
271
215
188
144
243
116
59
87
163
303
334353
390 415
457
40 60 80 100120140160180200220240260280300320340360380400420440460
m/z-->
S
H
N
O
NH2
HN
CN
O
N
NH
NC
O

MS of 5c

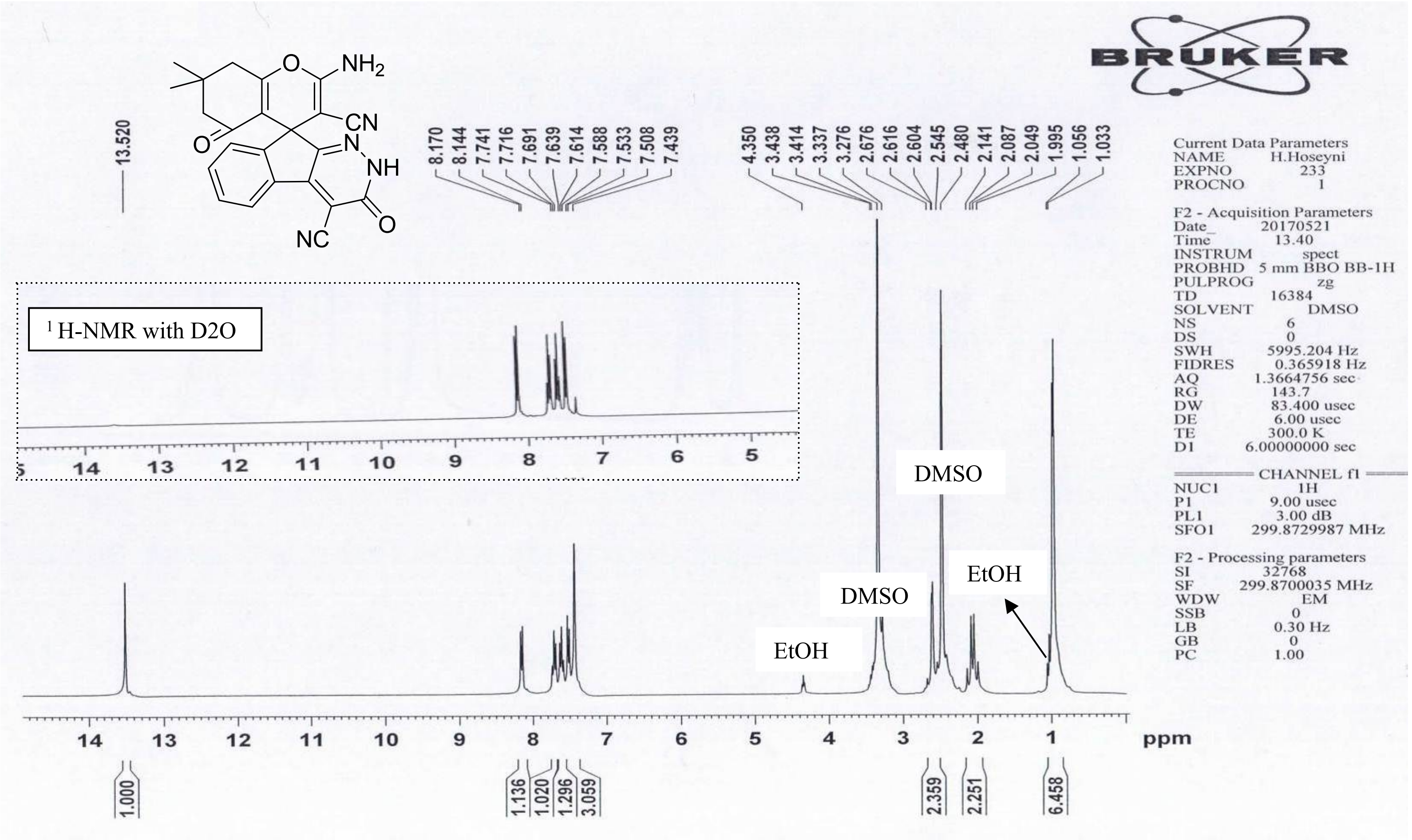

NH2
O
CN
N
NH
O
NC
O
BRUKER
13.520
8.170 8.144 7.741 7.716 7.691 7.639 7.614 7.588 7.533 7.508 7.439
4.350 3.438 3.414 3.337 3.276 2.676 2.616 2.604 2.545 2.480 2.141 2.087 2.049 1.995 1.056 1.033
1 H-NMR with D2O
DMSO
DMSO
EtOH
EtOH
ppm
1.000
1.136 1.020 1.296 3.059
2.359 2.251 6.458
Current Data Parameters
NAME H.Hoseyni
EXPNO 233
PROCNO 1
F2 - Acquisition Parameters
Date_ 20170521
Time 13.40
INSTRUM spect
PROBHD 5 mm BBO BB-1H
PULPROG zg
TD 16384
SOLVENT DMSO
NS 6
DS 0
SWH 5995.204 Hz
FIDRES 0.365918 Hz
AQ 1.3664756 sec
RG 143.7
DW 83.400 usec
DE 6.00 usec
TE 300.0 K
D1 6.00000000 sec
CHANNEL f1
NUC1 1H
P1 9.00 usec
PL1 3.00 dB
SFO1 299.8729987 MHz
F2 - Processing parameters
SI 32768
SF 299.8700035 MHz
WDW EM
SSB 0
LB 0.30 Hz
GB 0
PC 1.00

^{1}H NMR of 5d

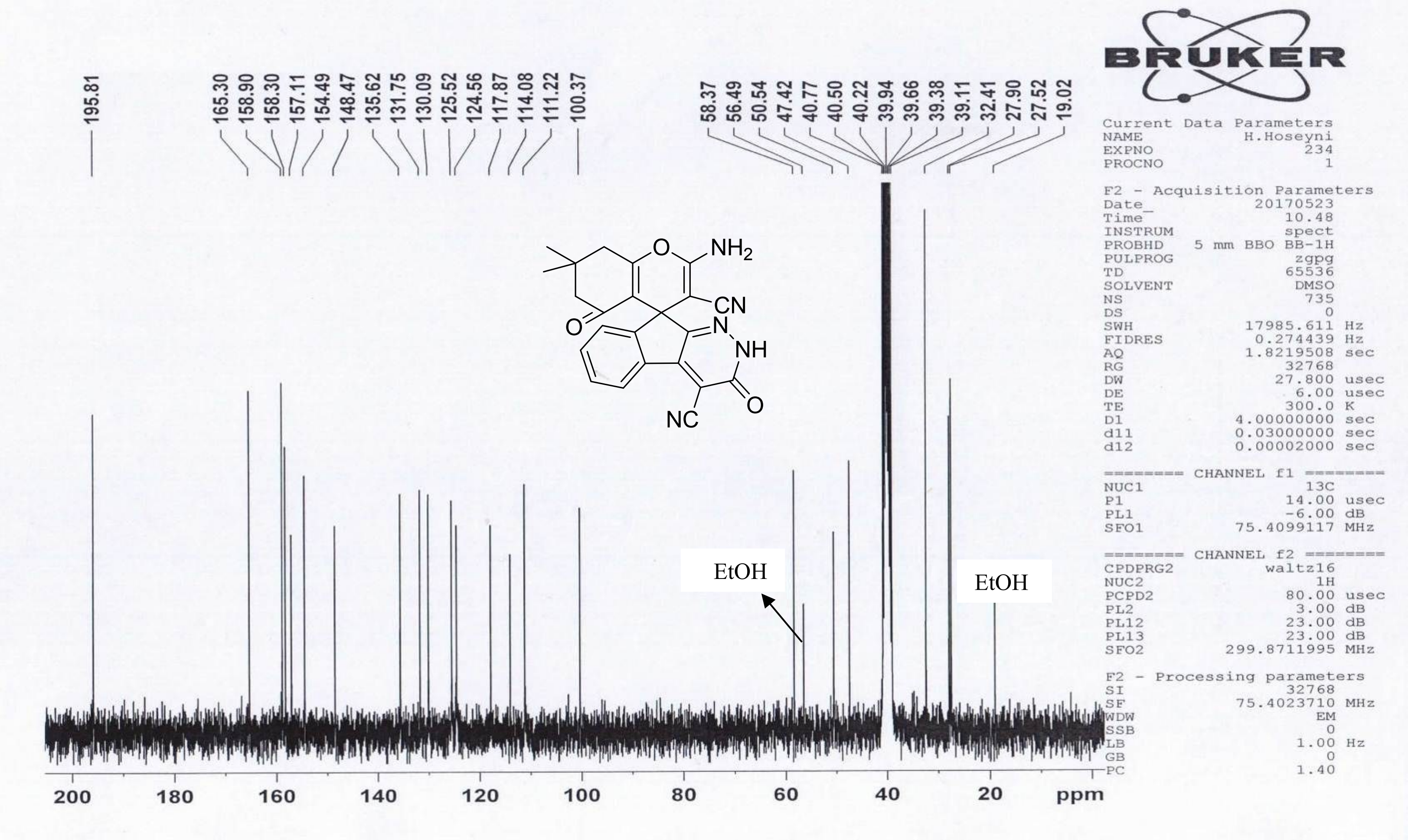
195.81
165.30
158.90
158.30
157.11
154.49
148.47
135.62
131.75
130.09
125.52
124.56
117.87
114.08
111.22
100.37
58.37
56.49
50.54
47.42
40.77
40.50
40.22
39.94
39.66
39.38
39.11
32.41
27.90
27.52
19.02
BRUKER
Current Data Parameters
NAME H.Hoseyni
EXPNO 234
PROCNO 1
F2 - Acquisition Parameters
Date_ 20170523
Time 10.48
INSTRUM spect
PROBHD 5 mm BBO BB-1H
PULPROG zgpg
TD 65536
SOLVENT DMSO
NS 735
DS 0
SWH 17985.611 Hz
FIDRES 0.274439 Hz
AQ 1.8219508 sec
RG 32768
DW 27.800 usec
DE 6.00 usec
TE 300.0 K
D1 4.00000000 sec
d11 0.03000000 sec
d12 0.00002000 sec
======== CHANNEL f1 ========
NUC1 13C
P1 14.00 usec
PL1 -6.00 dB
SFO1 75.4099117 MHz
======== CHANNEL f2 ========
CPDPRG2 waltz16
NUC2 1H
PCPD2 80.00 usec
PL2 3.00 dB
PL12 23.00 dB
PL13 23.00 dB
SFO2 299.8711995 MHz
F2 - Processing parameters
SI 32768
SF 75.4023710 MHz
WDW EM
SSB 0
LB 1.00 Hz
GB 0
PC 1.40
NH2
O
CN
N
NH
O
NC
O
EtOH
EtOH
200
180
160
140
120
100
80
60
40
20
ppm

^{13}C NMR of 5d

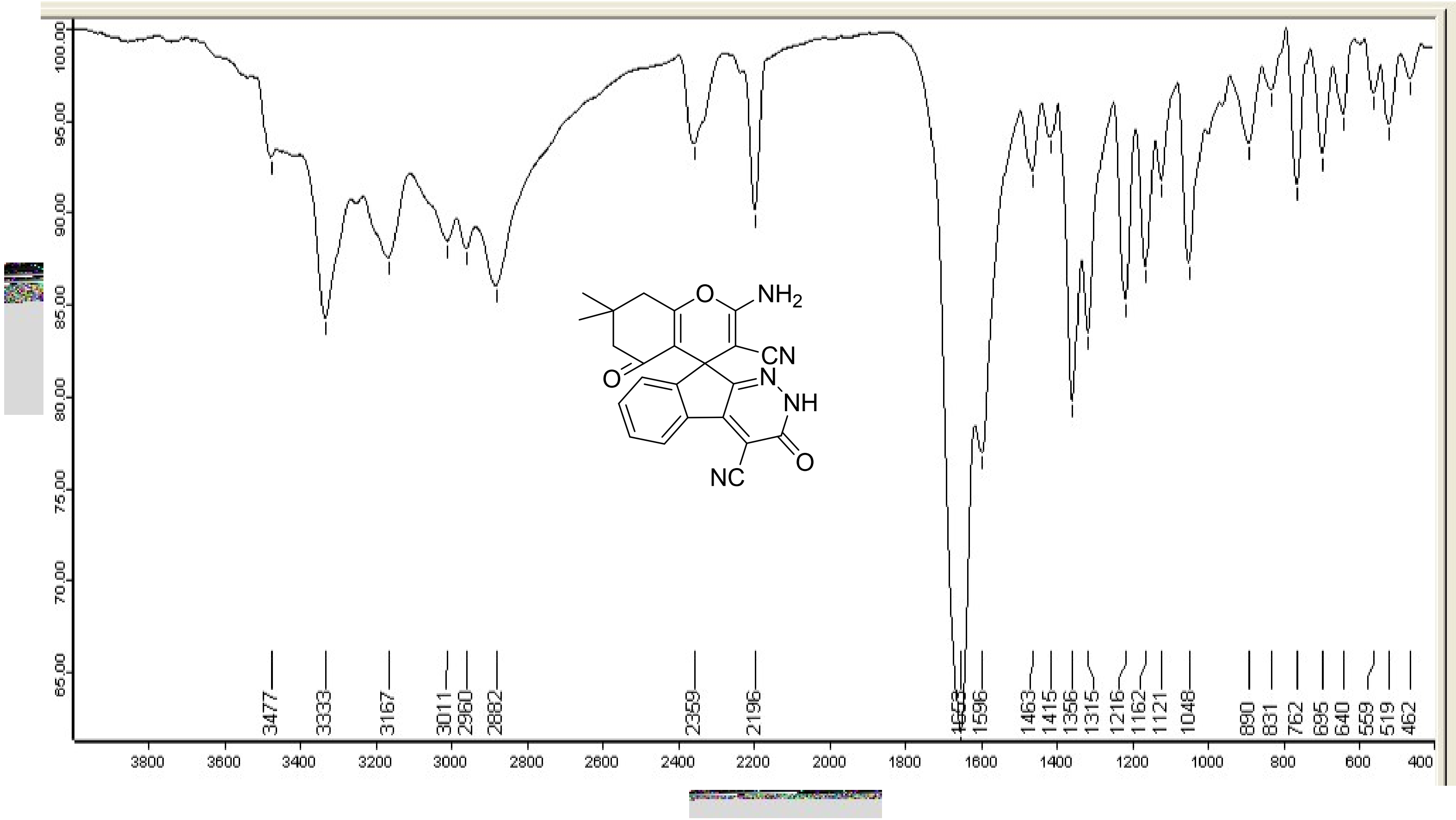

100.00
95.00
90.00
85.00
80.00
75.00
70.00
65.00
3477
3333
3167
3011
2960
2882
2359
2196
1596
1463
1415
1356
1315
1216
1162
1121
1048
890
831
762
695
640
559
519
462
3800
3600
3400
3200
3000
2800
2600
2400
2200
2000
1800
1600
1400
1200
1000
800
600
400
O
NH2
CN
N
NH
O
NC
O

IR of 5d

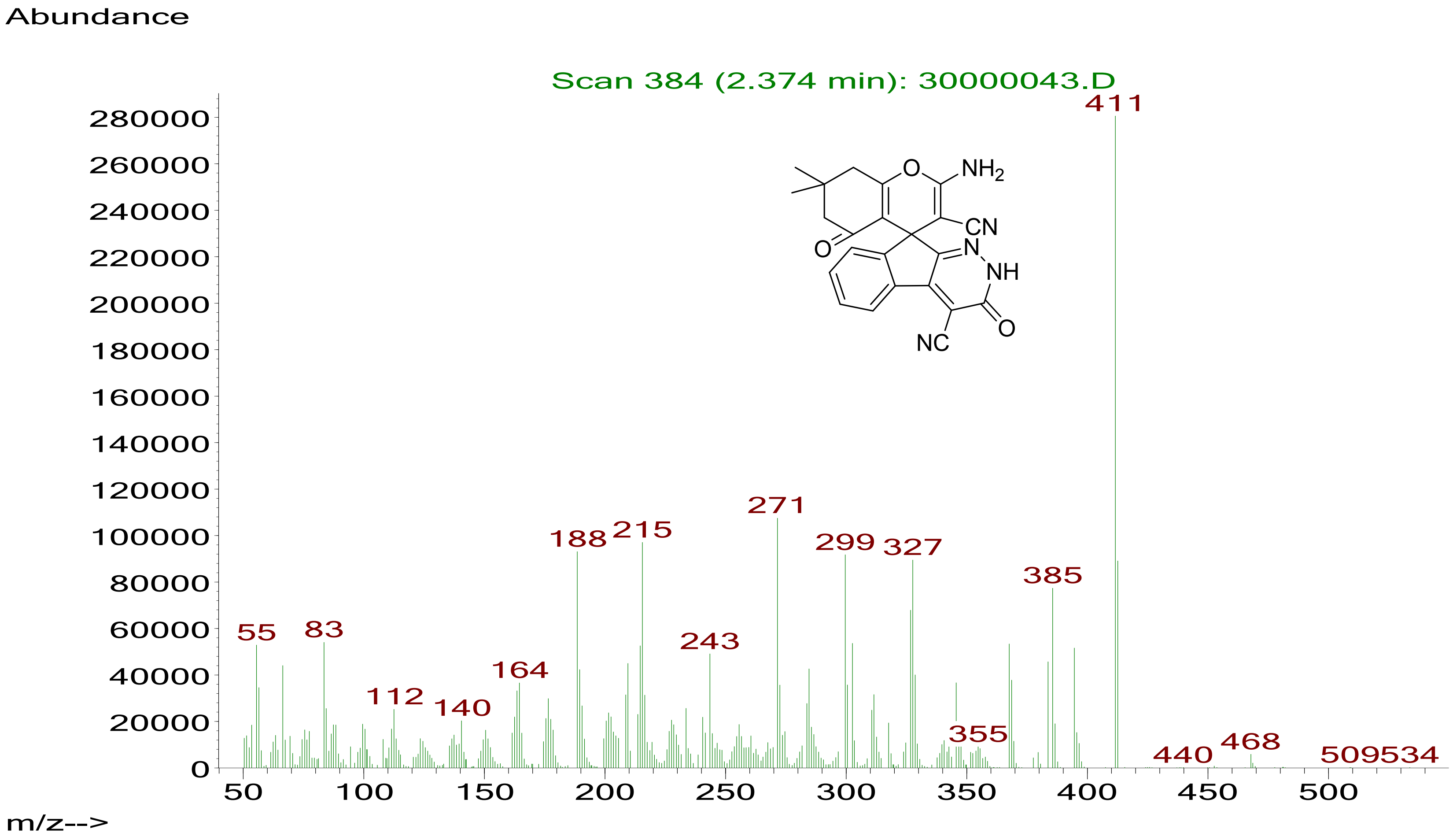
Abundance
Scan 384 (2.374 min): 30000043.D
280000
260000
240000
220000
200000
180000
160000
140000
120000
100000
80000
60000
40000
20000
0
411
385
355
327
299
271
243
215
188
164
140
112
83
55
440
468
509534
50
100
150
200
250
300
350
400
450
500
m/z-->
NH2
CN
N
NH
O
O
O
NC

MS of 5d

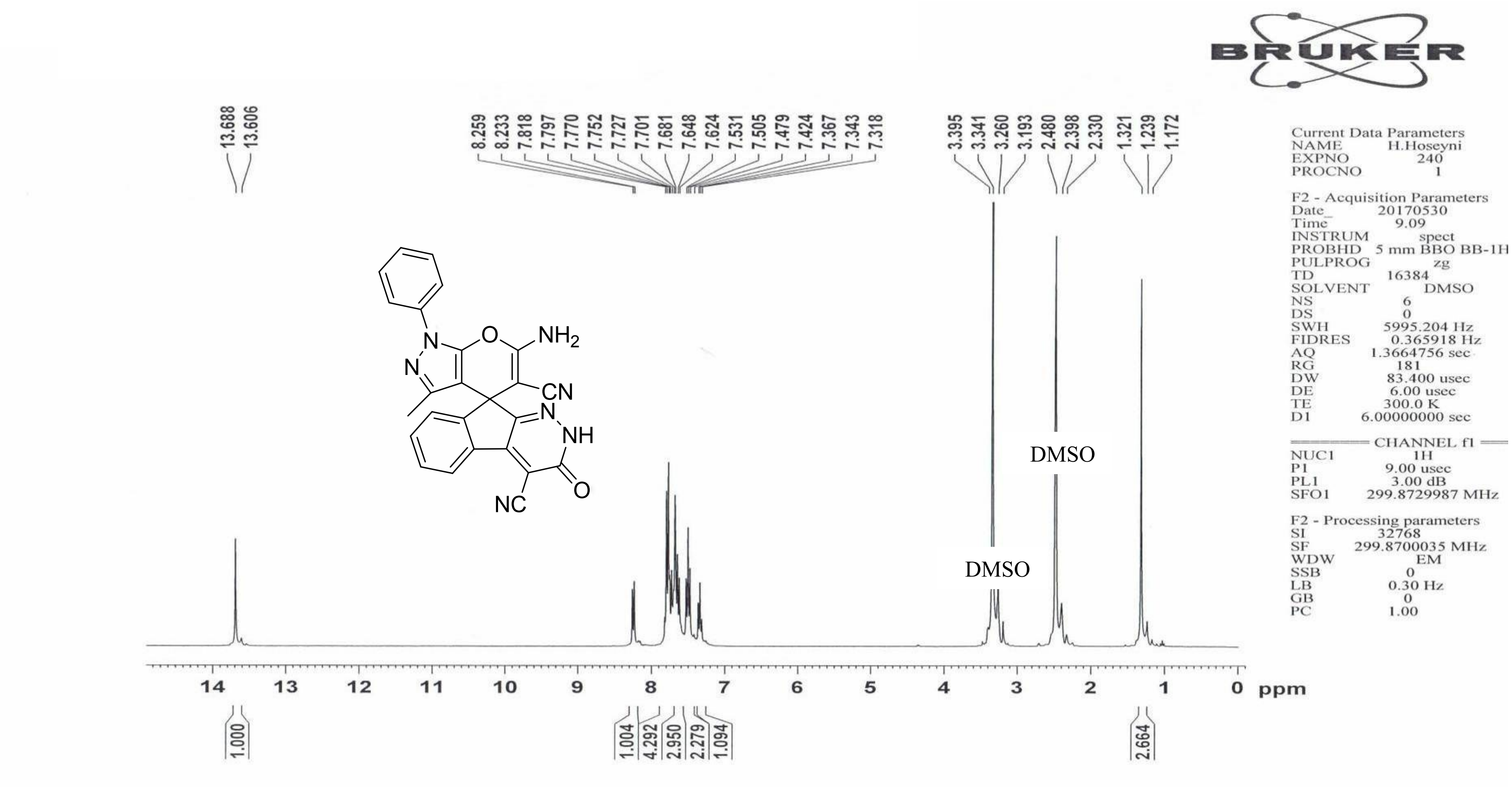
BRUKER
13.688
13.606
8.259
8.233
7.818
7.797
7.770
7.752
7.727
7.701
7.681
7.648
7.624
7.531
7.505
7.479
7.424
7.367
7.343
7.318
3.395
3.341
3.260
3.193
2.480
2.398
2.330
1.321
1.239
1.172
NH2
N
O
N
CN
N
NH
O
NC
DMSO
DMSO
14 13 12 11 10 9 8 7 6 5 4 3 2 1 0 ppm
1.000
1.004
4.292
2.950
2.279
1.094
2.664
Current Data Parameters
NAME H.Hoseyni
EXPNO 240
PROCNO 1
F2 - Acquisition Parameters
Date_ 20170530
Time 9.09
INSTRUM spect
PROBHD 5 mm BBO BB-1H
PULPROG zg
TD 16384
SOLVENT DMSO
NS 6
DS 0
SWH 5995.204 Hz
FIDRES 0.365918 Hz
AQ 1.3664756 sec
RG 181
DW 83.400 usec
DE 6.00 usec
TE 300.0 K
D1 6.00000000 sec
CHANNEL f1
NUC1 1H
P1 9.00 usec
PL1 3.00 dB
SFO1 299.8729987 MHz
F2 - Processing parameters
SI 32768
SF 299.8700035 MHz
WDW EM
SSB 0
LB 0.30 Hz
GB 0
PC 1.00

^{1}H NMR of 5e

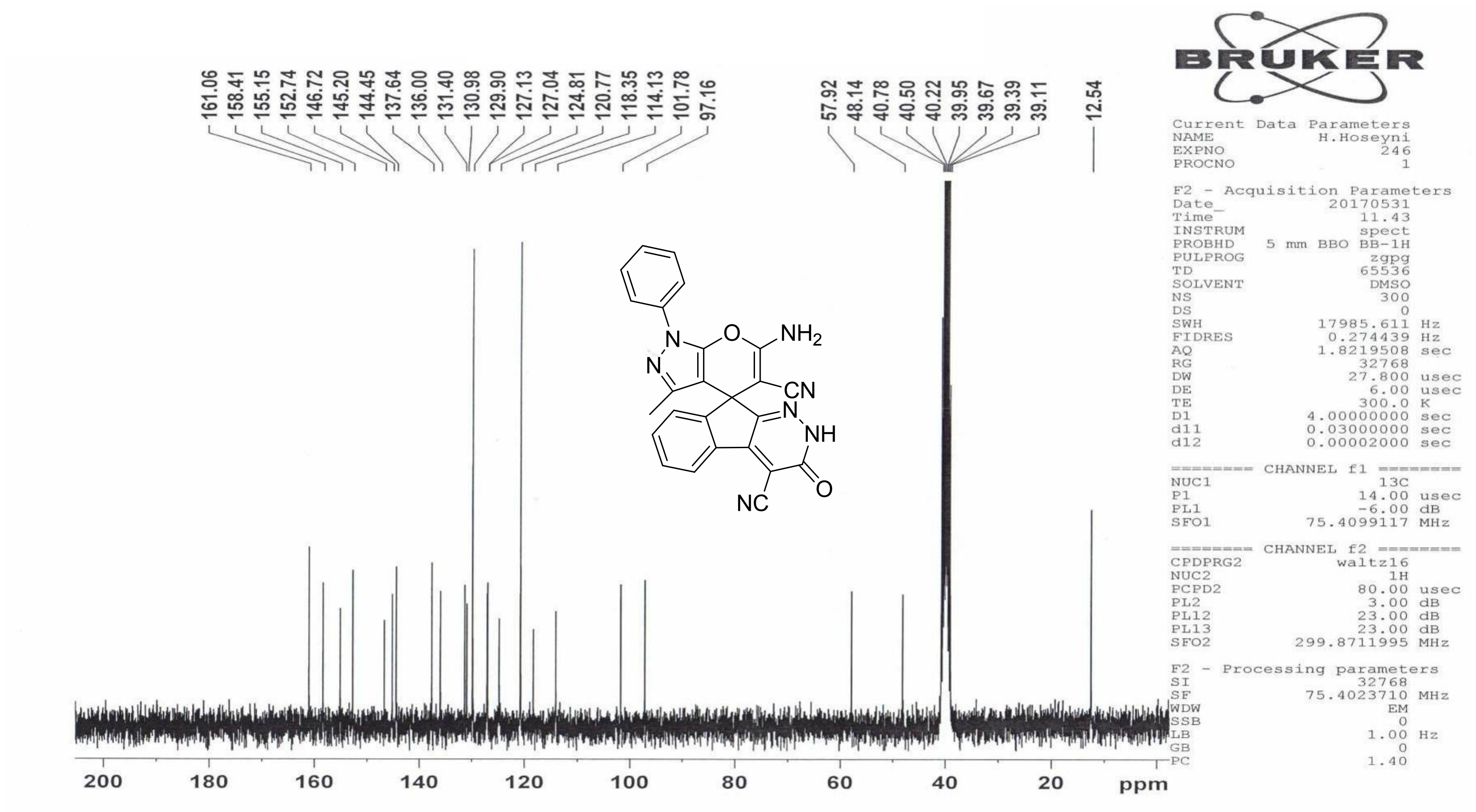

161.06
158.41
155.15
152.74
146.72
145.20
144.45
137.64
136.00
131.40
130.98
129.90
127.13
127.04
124.81
120.77
118.35
114.13
101.78
97.16
57.92
48.14
40.78
40.50
40.22
39.95
39.67
39.39
39.11
12.54
BRUKER
Current Data Parameters
NAME H.Hoseyni
EXPNO 246
PROCNO 1
F2 - Acquisition Parameters
Date_ 20170531
Time 11.43
INSTRUM spect
PROBHD 5 mm BBO BB-1H
PULPROG zgpg
TD 65536
SOLVENT DMSO
NS 300
DS 0
SWH 17985.611 Hz
FIDRES 0.274439 Hz
AQ 1.8219508 sec
RG 32768
DW 27.800 usec
DE 6.00 usec
TE 300.0 K
D1 4.00000000 sec
d11 0.03000000 sec
d12 0.00002000 sec
======== CHANNEL f1 ========
NUC1 13C
P1 14.00 usec
PL1 -6.00 dB
SFO1 75.4099117 MHz
======== CHANNEL f2 ========
CPDPRG2 waltz16
NUC2 1H
PCPD2 80.00 usec
PL2 3.00 dB
PL12 23.00 dB
PL13 23.00 dB
SFO2 299.8711995 MHz
F2 - Processing parameters
SI 32768
SF 75.4023710 MHz
WDW EM
SSB 0
LB 1.00 Hz
GB 0
PC 1.40
NH2
CN
N
N
O
N
NH
O
NC
200
180
160
140
120
100
80
60
40
20
ppm

^{13}C NMR of 5e

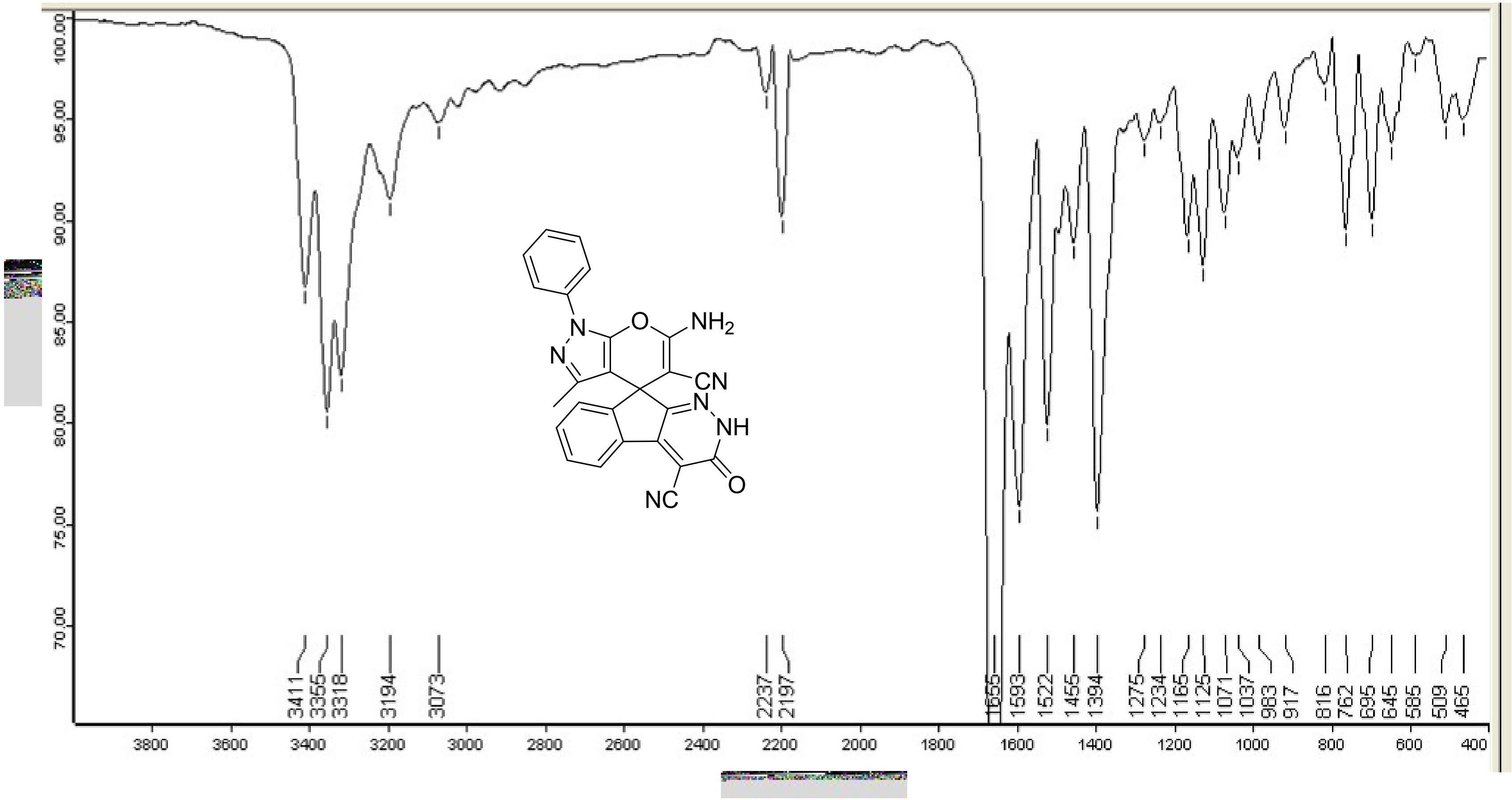
100,00
95,00
90,00
85,00
80,00
75,00
70,00
NH2
CN
N
NH
O
NC
3411
3355
3318
3194
3073
2237
2197
1655
1593
1522
1455
1394
1275
1234
1165
1125
1071
1037
983
917
816
762
695
645
585
509
465
3800
3600
3400
3200
3000
2800
2600
2400
2200
2000
1800
1600
1400
1200
1000
800
600
400

IR of 5e

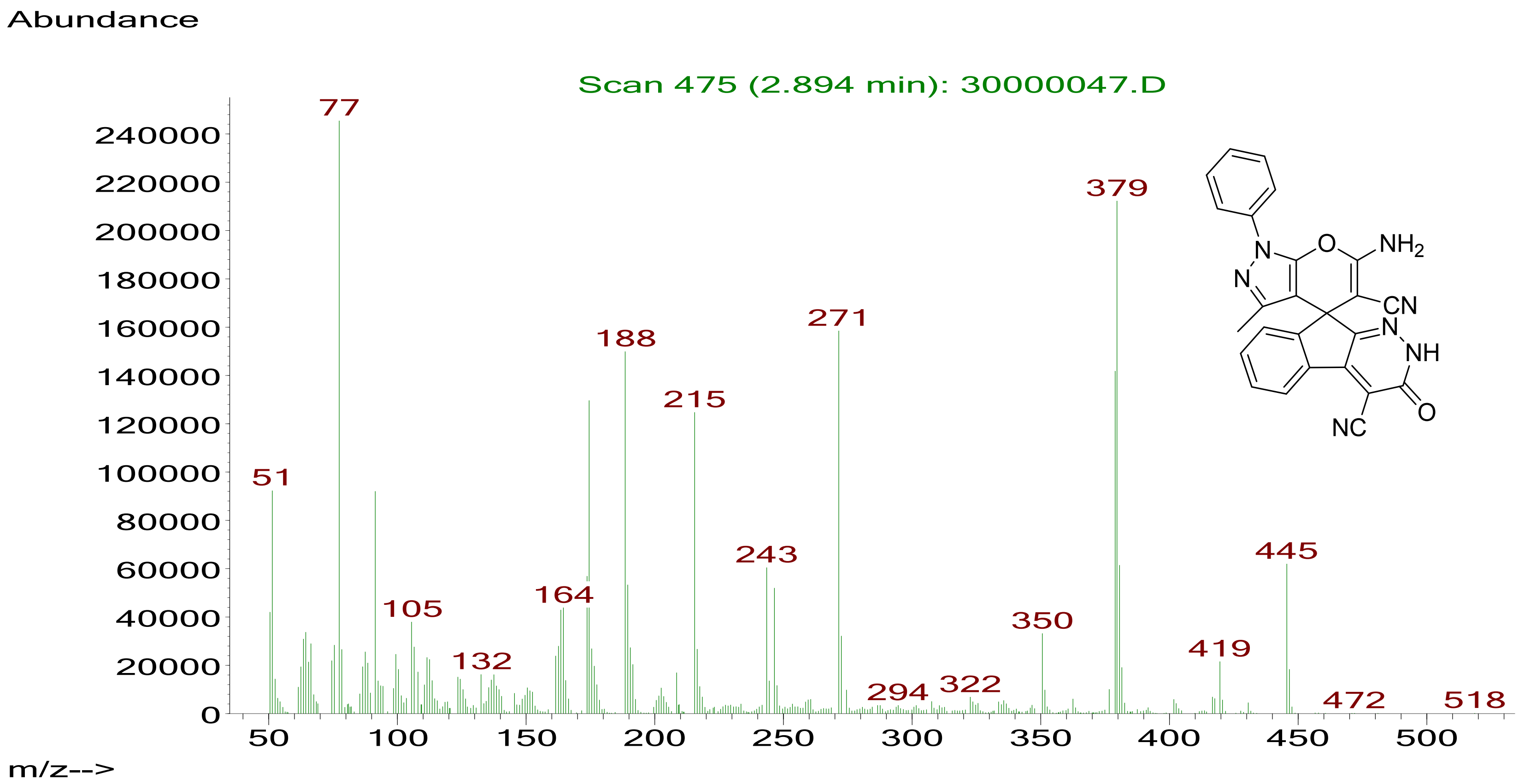

Abundance
Scan 475 (2.894 min): 30000047.D
240000
220000
200000
180000
160000
140000
120000
100000
80000
60000
40000
20000
0
77
379
271
188
215
51
243
445
105
164
350
132
419
294
322
472
518
50
100
150
200
250
300
350
400
450
500
m/z-->
N
N
O
NH2
CN
N
NH
O
NC

MS of 5e

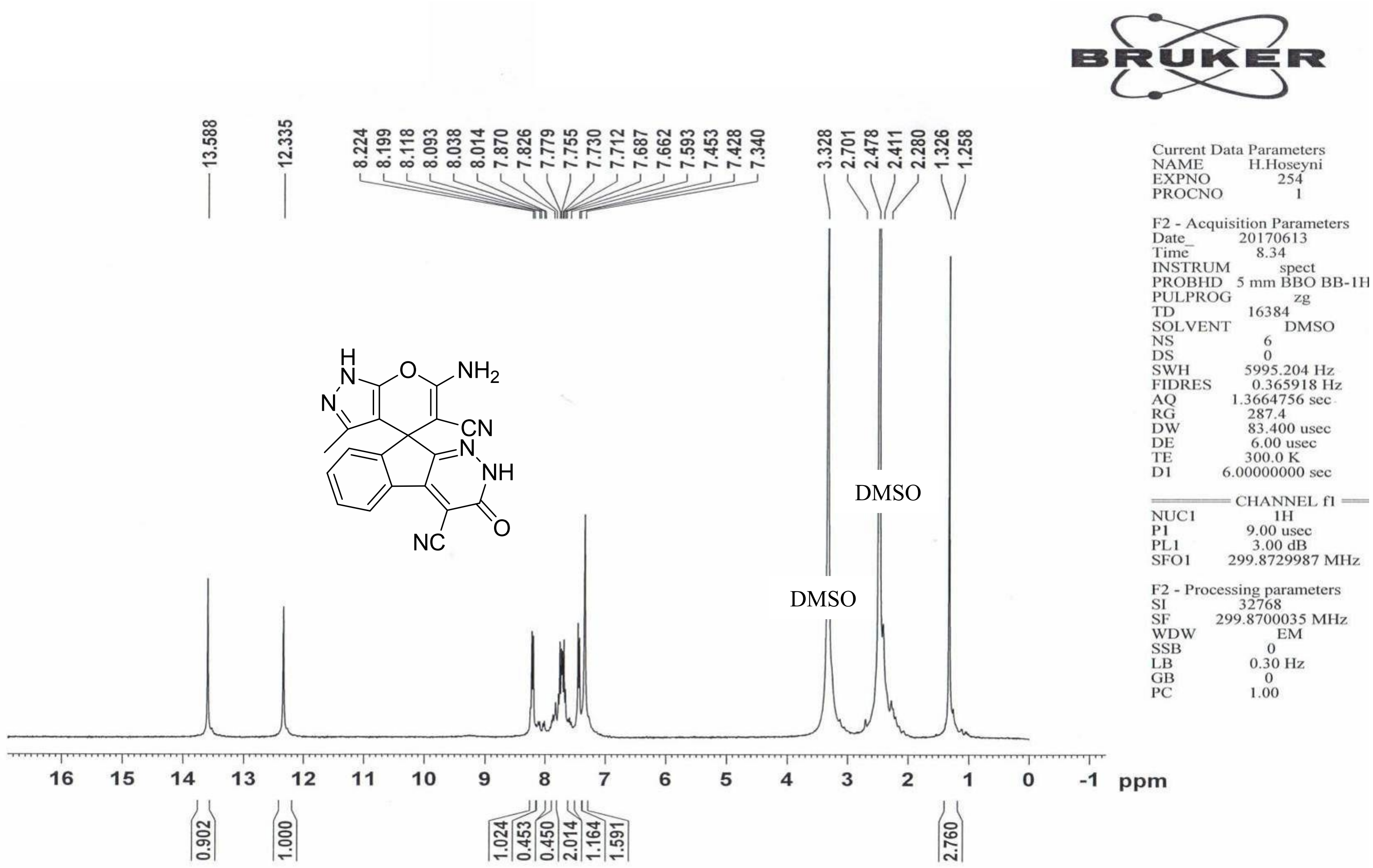
BRUKER
13.588
12.335
8.224
8.199
8.118
8.093
8.038
8.014
7.870
7.826
7.779
7.755
7.730
7.712
7.687
7.662
7.593
7.453
7.428
7.340
3.328
2.701
2.478
2.411
2.280
1.326
1.258
Current Data Parameters
NAME H.Hoseyni
EXPNO 254
PROCNO 1
F2 - Acquisition Parameters
Date_ 20170613
Time 8.34
INSTRUM spect
PROBHD 5 mm BBO BB-1H
PULPROG zg
TD 16384
SOLVENT DMSO
NS 6
DS 0
SWH 5995.204 Hz
FIDRES 0.365918 Hz
AQ 1.3664756 sec
RG 287.4
DW 83.400 usec
DE 6.00 usec
TE 300.0 K
D1 6.00000000 sec
CHANNEL f1
NUC1 1H
P1 9.00 usec
PL1 3.00 dB
SFO1 299.8729987 MHz
F2 - Processing parameters
SI 32768
SF 299.8700035 MHz
WDW EM
SSB 0
LB 0.30 Hz
GB 0
PC 1.00
DMSO
DMSO
NH2
CN
NH
O
NC
ppm
0.902
1.000
1.024
0.453
0.450
2.014
1.164
1.591
2.760

^{1}H NMR of 5f

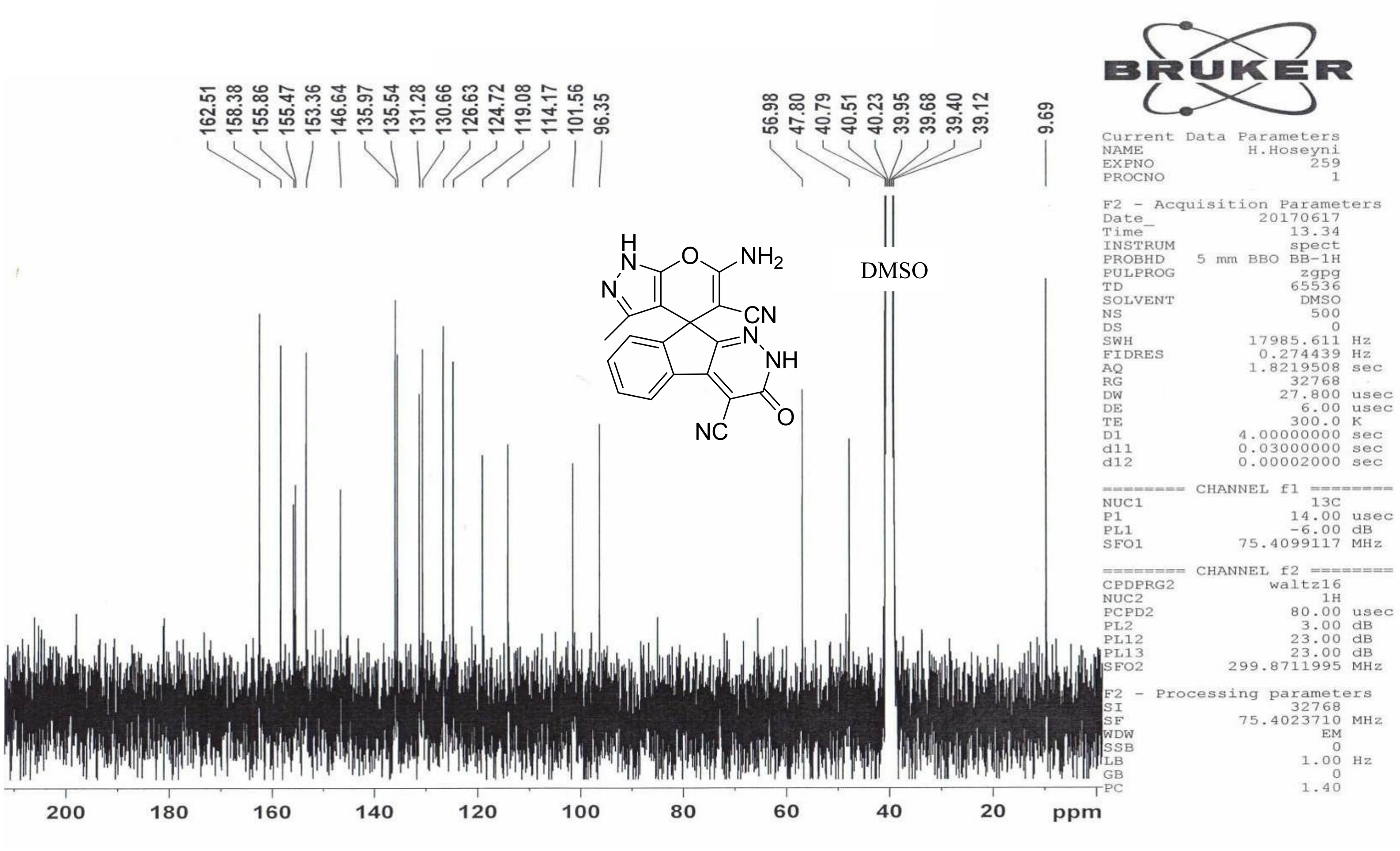
162.51
158.38
155.86
155.47
153.36
146.64
135.97
135.54
131.28
130.66
126.63
124.72
119.08
114.17
101.56
96.35
56.98
47.80
40.79
40.51
40.23
39.95
39.68
39.40
39.12
9.69
DMSO
Current Data Parameters
NAME H.Hoseyni
EXPNO 259
PROCNO 1
F2 - Acquisition Parameters
Date_ 20170617
Time 13.34
INSTRUM spect
PROBHD 5 mm BBO BB-1H
PULPROG zgpg
TD 65536
SOLVENT DMSO
NS 500
DS 0
SWH 17985.611 Hz
FIDRES 0.274439 Hz
AQ 1.8219508 sec
RG 32768
DW 27.800 usec
DE 6.00 usec
TE 300.0 K
D1 4.00000000 sec
d11 0.03000000 sec
d12 0.00002000 sec
======== CHANNEL f1 ========
NUC1 13C
P1 14.00 usec
PL1 -6.00 dB
SFO1 75.4099117 MHz
======== CHANNEL f2 ========
CPDPRG2 waltz16
NUC2 1H
PCPD2 80.00 usec
PL2 3.00 dB
PL12 23.00 dB
PL13 23.00 dB
SFO2 299.8711995 MHz
F2 - Processing parameters
SI 32768
SF 75.4023710 MHz
WDW EM
SSB 0
LB 1.00 Hz
GB 0
PC 1.40
200 180 160 140 120 100 80 60 40 20 ppm

^{13}C NMR of 5f

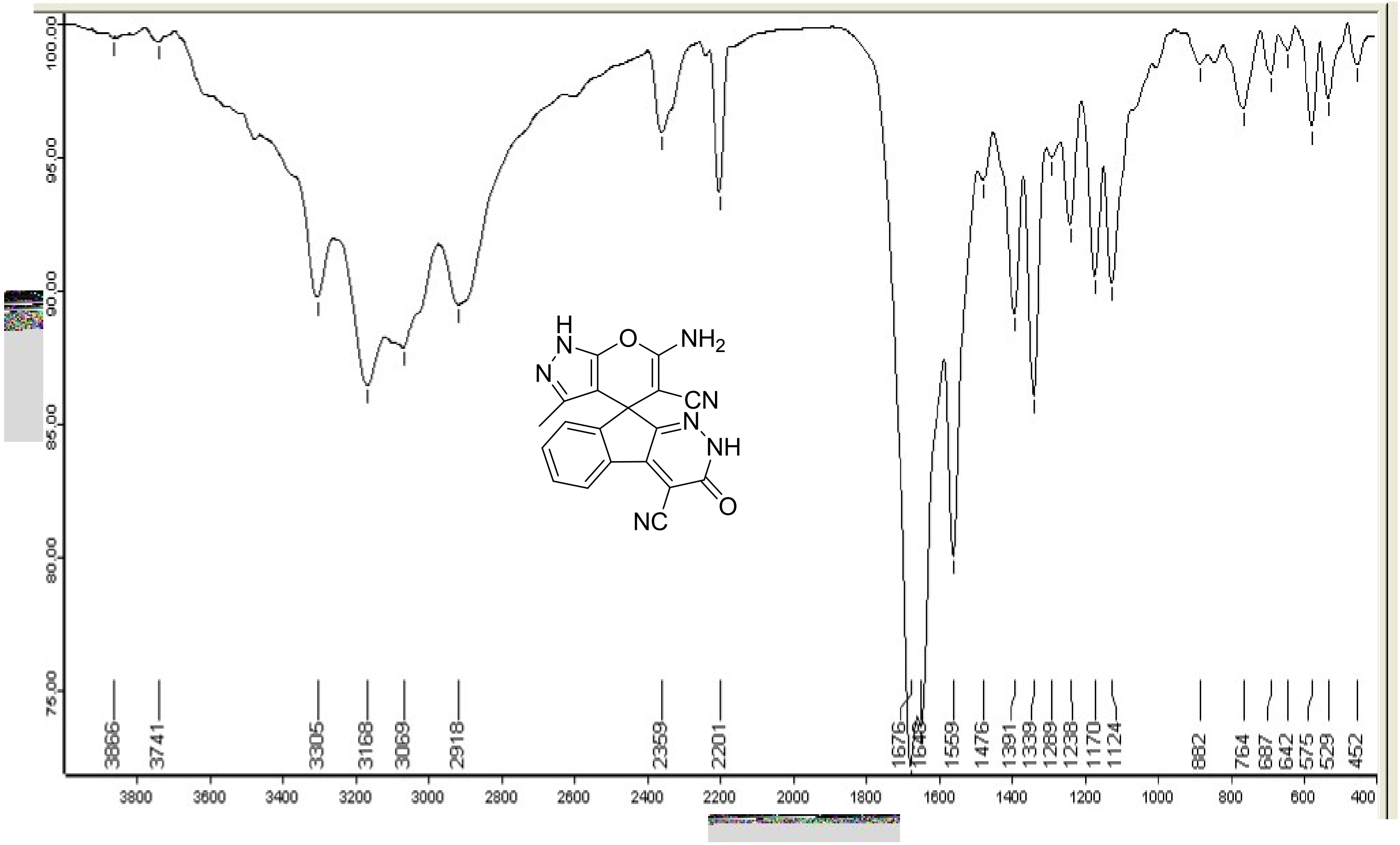
100,00
95,00
90,00
85,00
80,00
75,00
3866
3741
3305
3168
3069
2918
2359
2201
1676
1643
1559
1476
1391
1339
1289
1238
1170
1124
882
764
687
642
575
529
452
3800
3600
3400
3200
3000
2800
2600
2400
2200
2000
1800
1600
1400
1200
1000
800
600
400
H
N
N
O
NH_2
CN
N
NH
O
NC

IR of 5f

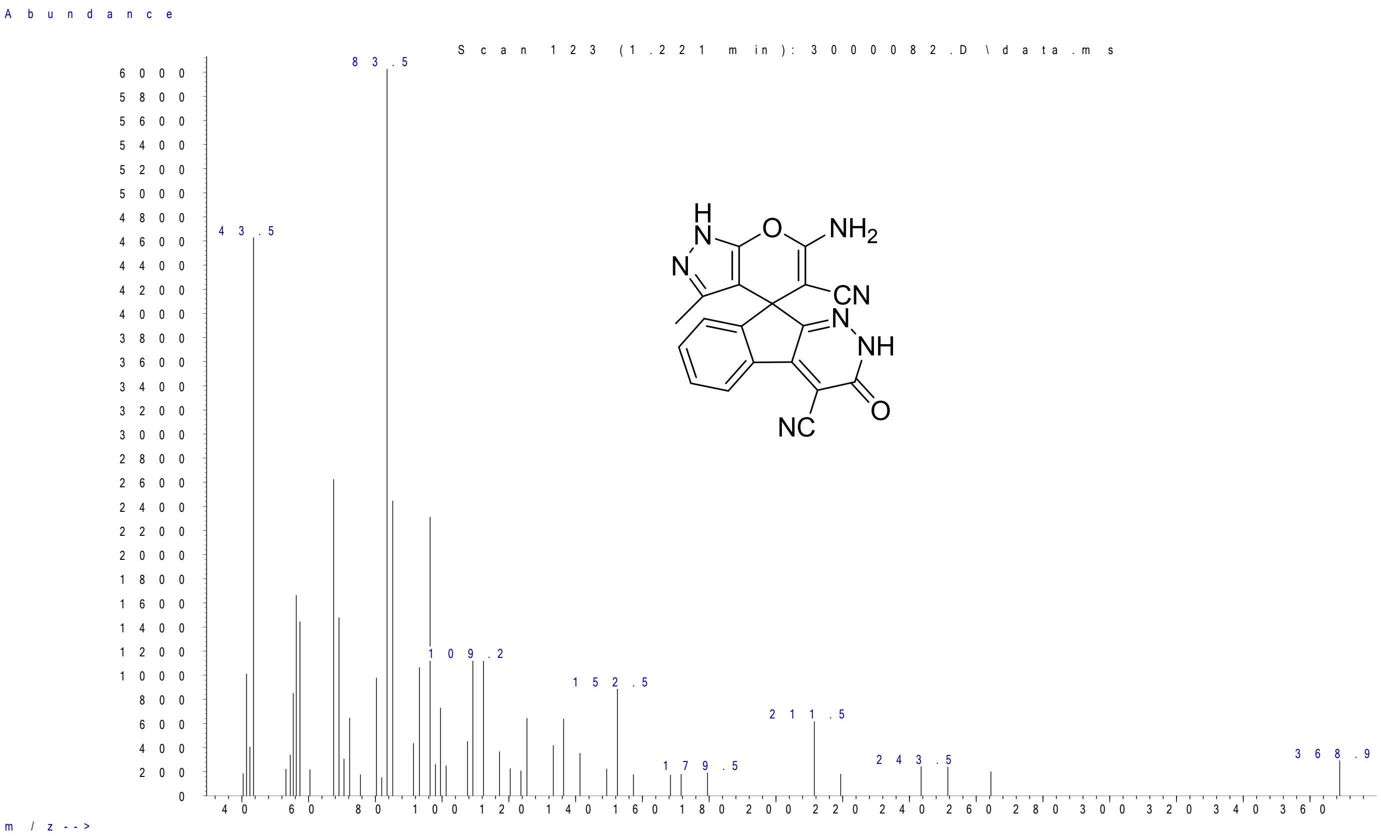

Abundance
Scan 123 (1.221 min): 3000082.D\data.ms
83.5
43.5
109.2
152.5
211.5
179.5
243.5
368.9
m/z-->
NH
N
O
NH2
CN
N
NH
O
NC

MS of 5f

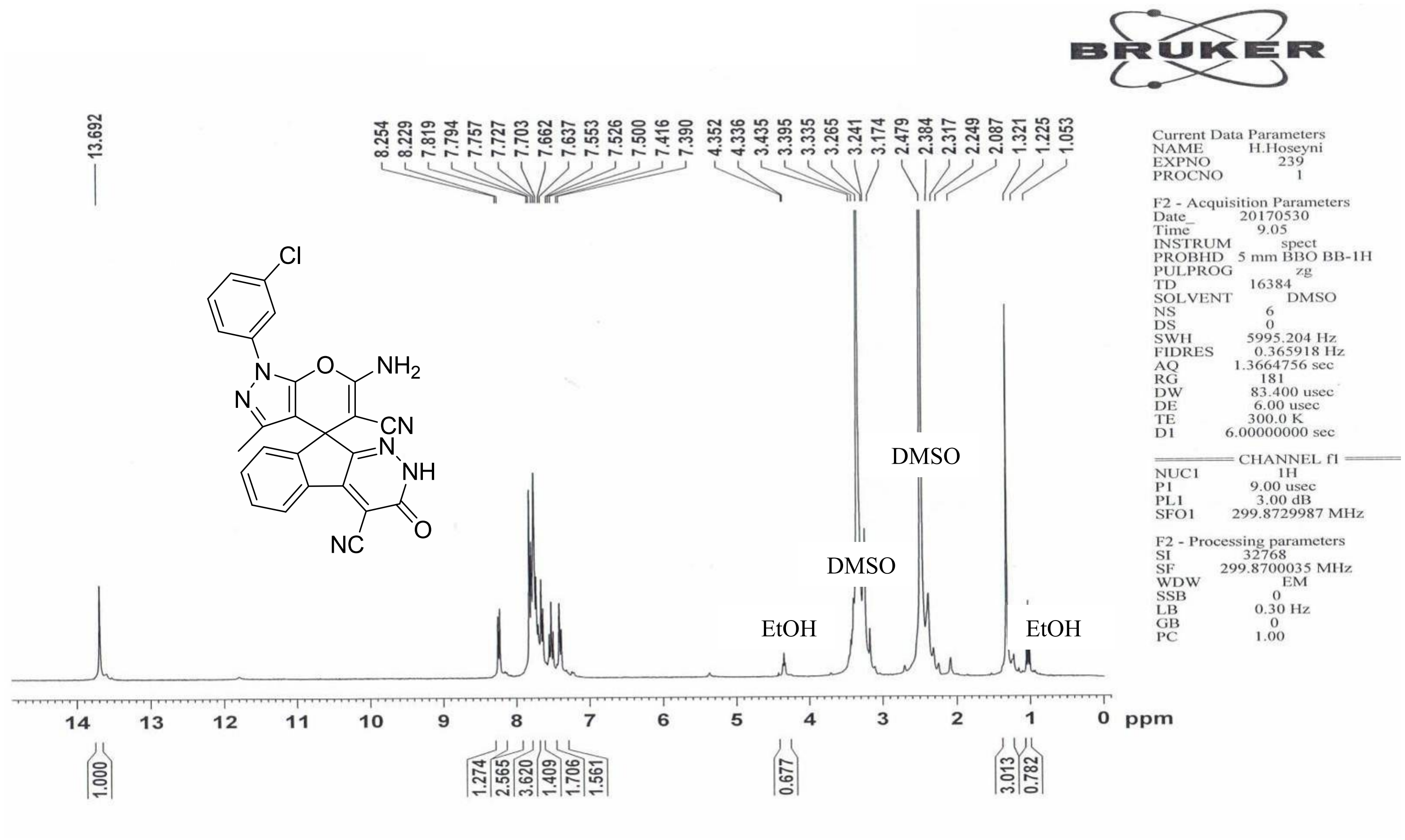

13.692
8.254
8.229
7.819
7.794
7.757
7.727
7.703
7.662
7.637
7.553
7.526
7.500
7.416
7.390
4.352
4.336
3.435
3.395
3.335
3.265
3.241
3.174
2.479
2.384
2.317
2.249
2.087
1.321
1.225
1.053
Current Data Parameters
NAME H.Hoseyni
EXPNO 239
PROCNO 1
F2 - Acquisition Parameters
Date_ 20170530
Time 9.05
INSTRUM spect
PROBHD 5 mm BBO BB-1H
PULPROG zg
TD 16384
SOLVENT DMSO
NS 6
DS 0
SWH 5995.204 Hz
FIDRES 0.365918 Hz
AQ 1.3664756 sec
RG 181
DW 83.400 usec
DE 6.00 usec
TE 300.0 K
D1 6.00000000 sec
CHANNEL f1
NUC1 1H
P1 9.00 usec
PL1 3.00 dB
SFO1 299.8729987 MHz
F2 - Processing parameters
SI 32768
SF 299.8700035 MHz
WDW EM
SSB 0
LB 0.30 Hz
GB 0
PC 1.00
Cl
N
O
NH2
N
CN
N
NH
NC
O
DMSO
DMSO
EtOH
EtOH
14 13 12 11 10 9 8 7 6 5 4 3 2 1 0 ppm
1.000
1.274
2.565
3.620
1.409
1.706
1.561
0.677
3.013
0.782

^{1}H NMR of 5g

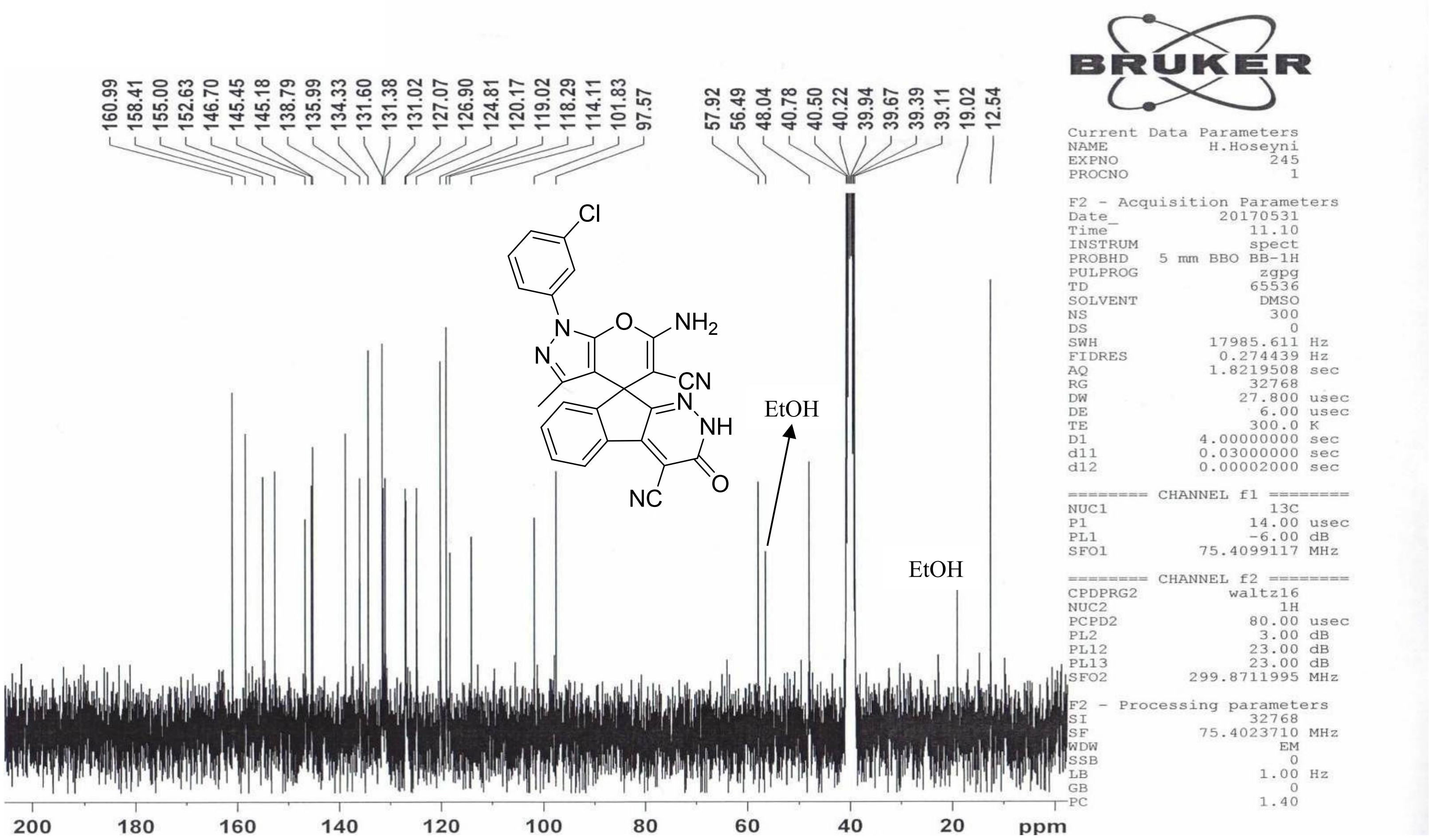
160.99
158.41
155.00
152.63
146.70
145.45
145.18
138.79
135.99
134.33
131.60
131.38
131.02
127.07
126.90
124.81
120.17
119.02
118.29
114.11
101.83
97.57
57.92
56.49
48.04
40.78
40.50
40.22
39.94
39.67
39.39
39.11
19.02
12.54
Cl
N
N
O
NH2
CN
N
NH
O
NC
EtOH
EtOH
BRUKER
Current Data Parameters
NAME H.Hoseyni
EXPNO 245
PROCNO 1
F2 - Acquisition Parameters
Date_ 20170531
Time 11.10
INSTRUM spect
PROBHD 5 mm BBO BB-1H
PULPROG zgpg
TD 65536
SOLVENT DMSO
NS 300
DS 0
SWH 17985.611 Hz
FIDRES 0.274439 Hz
AQ 1.8219508 sec
RG 32768
DW 27.800 usec
DE 6.00 usec
TE 300.0 K
D1 4.00000000 sec
d11 0.03000000 sec
d12 0.00002000 sec
======== CHANNEL f1 ========
NUC1 13C
P1 14.00 usec
PL1 -6.00 dB
SFO1 75.4099117 MHz
======== CHANNEL f2 ========
CPDPRG2 waltz16
NUC2 1H
PCPD2 80.00 usec
PL2 3.00 dB
PL12 23.00 dB
PL13 23.00 dB
SFO2 299.8711995 MHz
F2 - Processing parameters
SI 32768
SF 75.4023710 MHz
WDW EM
SSB 0
LB 1.00 Hz
GB 0
PC 1.40
200
180
160
140
120
100
80
60
40
20
ppm

^{13}C NMR of 5g

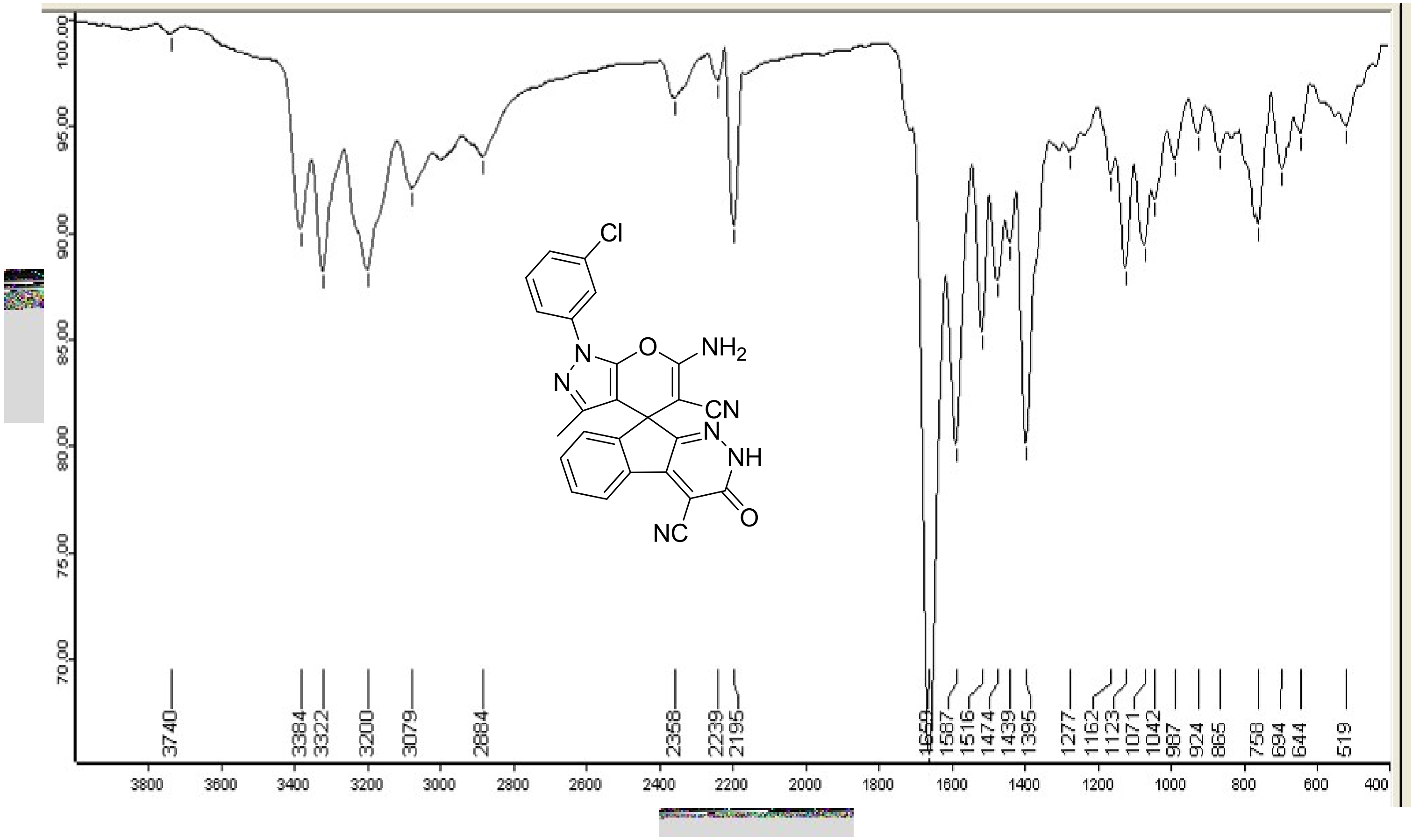
100,00
95,00
90,00
85,00
80,00
75,00
70,00
3740
3384
3322
3200
3079
2884
2358
2239
2195
1659
1587
1516
1474
1439
1395
1277
1162
1123
1071
1042
987
924
865
758
694
644
519
3800
3600
3400
3200
3000
2800
2600
2400
2200
2000
1800
1600
1400
1200
1000
800
600
400
Cl
N
O
NH2
N
CN
N
NH
O
NC

IR of 5g

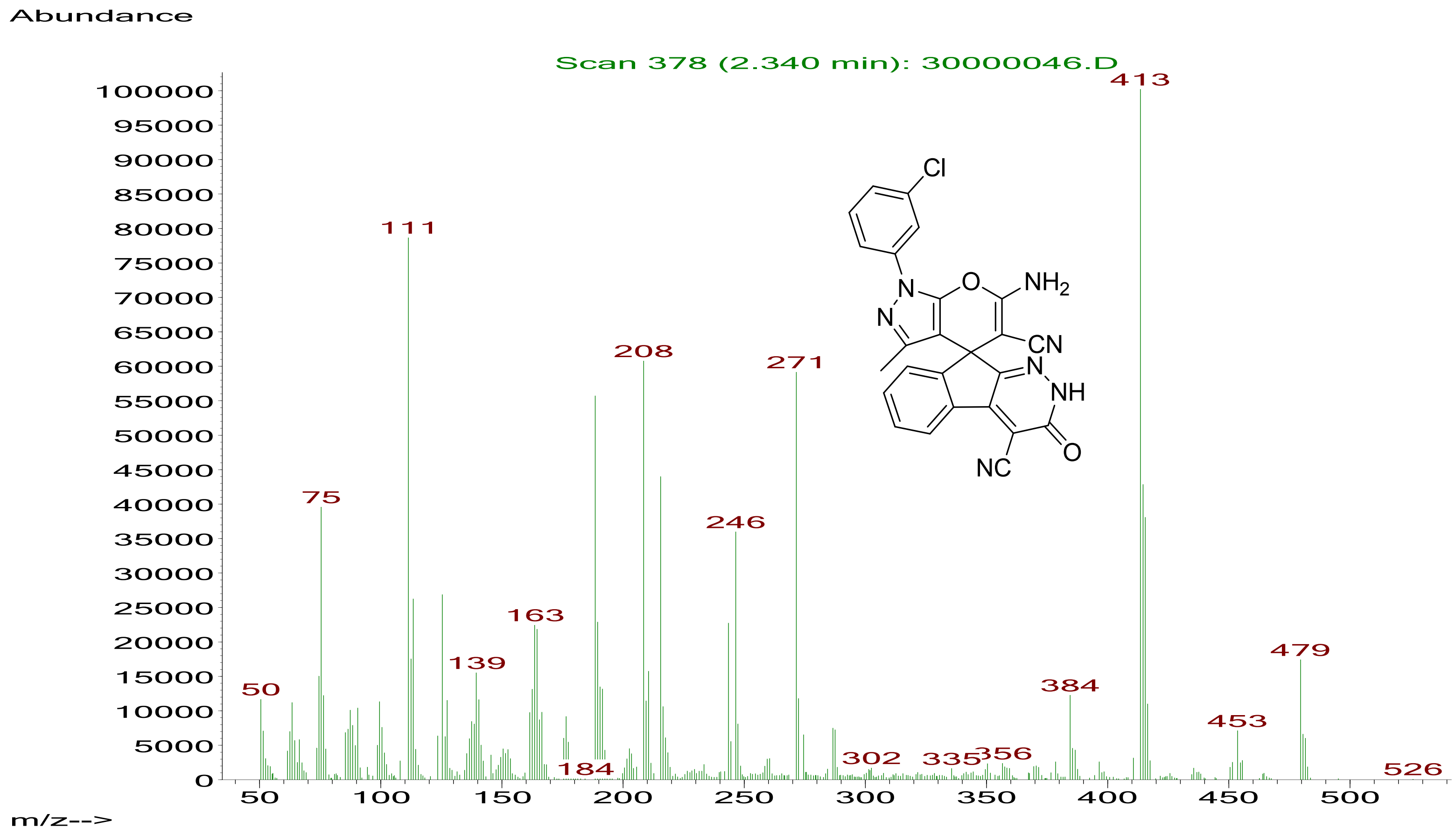
Abundance
Scan 378 (2.340 min): 30000046.D
m/z-->
111
75
50
139
163
184
208
246
271
302
335
356
384
413
453
479
526
Cl
N
O
NH2
CN
NH
NC

MS of 5g

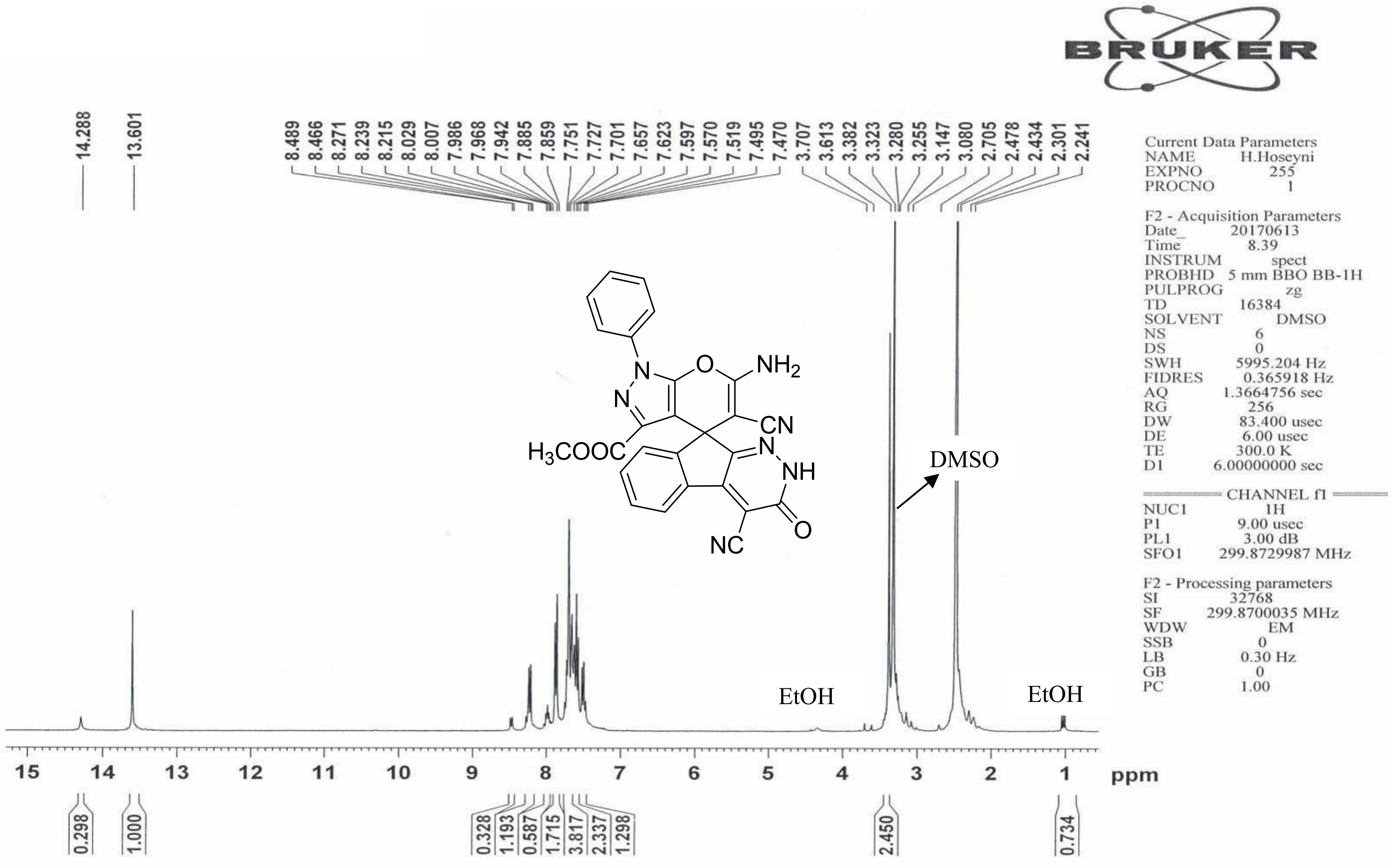
BRUKER
14.288
13.601
8.489
8.466
8.271
8.239
8.215
8.029
8.007
7.986
7.968
7.942
7.885
7.859
7.751
7.727
7.701
7.657
7.623
7.597
7.570
7.519
7.495
7.470
3.707
3.613
3.382
3.323
3.280
3.255
3.147
3.080
2.705
2.478
2.434
2.301
2.241
Current Data Parameters
NAME H.Hoseyni
EXPNO 255
PROCNO 1
F2 - Acquisition Parameters
Date_ 20170613
Time 8.39
INSTRUM spect
PROBHD 5 mm BBO BB-1H
PULPROG zg
TD 16384
SOLVENT DMSO
NS 6
DS 0
SWH 5995.204 Hz
FIDRES 0.365918 Hz
AQ 1.3664756 sec
RG 256
DW 83.400 usec
DE 6.00 usec
TE 300.0 K
D1 6.00000000 sec
CHANNEL f1
NUC1 1H
P1 9.00 usec
PL1 3.00 dB
SFO1 299.8729987 MHz
F2 - Processing parameters
SI 32768
SF 299.8700035 MHz
WDW EM
SSB 0
LB 0.30 Hz
GB 0
PC 1.00
N
O
NH2
N
CN
H3COOC
N
NH
O
NC
DMSO
EtOH
EtOH
15
14
13
12
11
10
9
8
7
6
5
4
3
2
1
ppm
0.298
1.000
0.328
1.193
0.587
1.715
3.817
2.337
1.298
2.450
0.734

^{1}H NMR of 5h

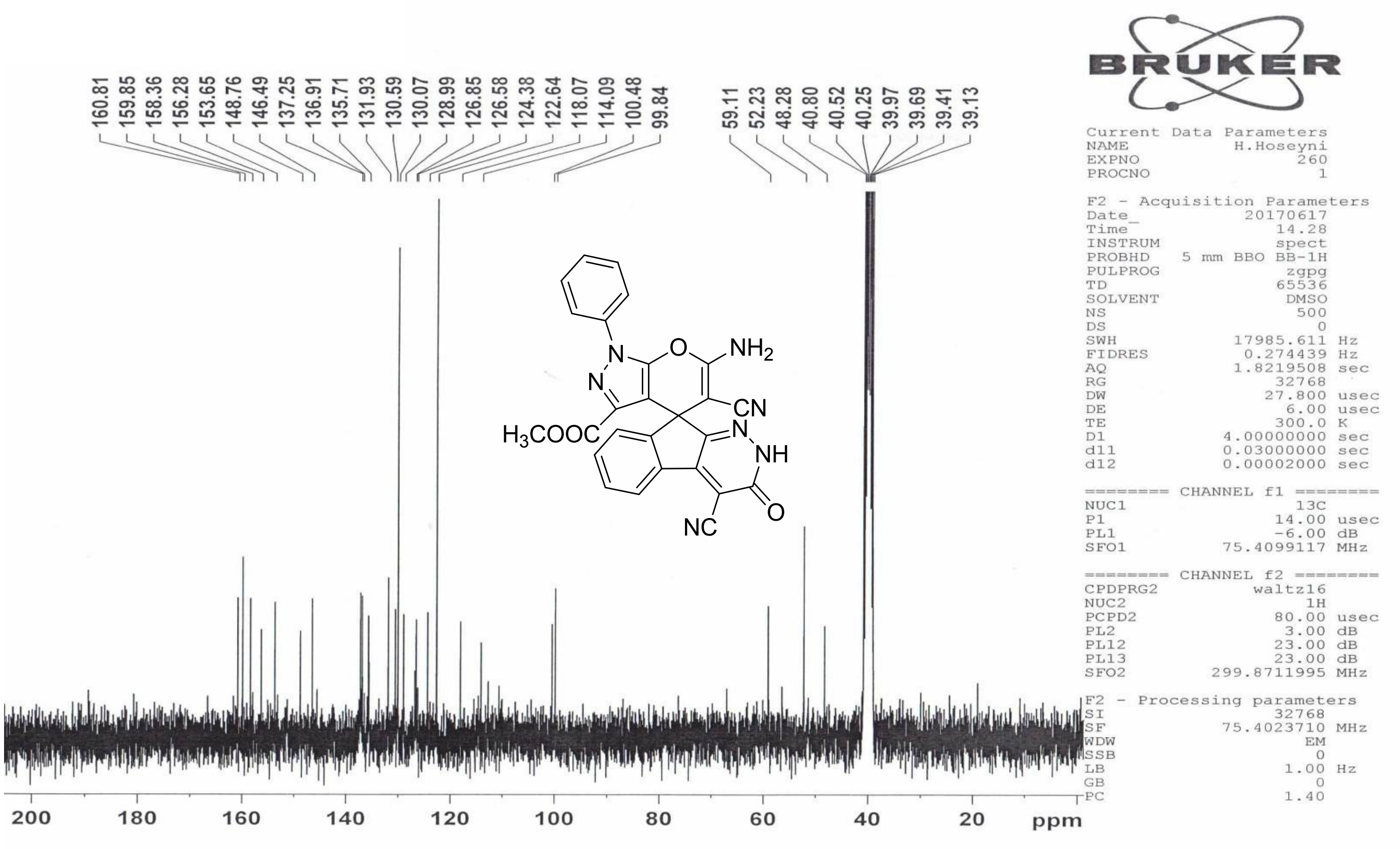

160.81 159.85 158.36 156.28 153.65 148.76 146.49 137.25 136.91 135.71 131.93 130.59 130.07 128.99 126.85 126.58 124.38 122.64 118.07 114.09 100.48 99.84
59.11 52.23 48.28 40.80 40.52 40.25 39.97 39.69 39.41 39.13
BRUKER
Current Data Parameters
NAME H.Hoseyni
EXPNO 260
PROCNO 1
F2 - Acquisition Parameters
Date_ 20170617
Time 14.28
INSTRUM spect
PROBHD 5 mm BBO BB-1H
PULPROG zgpg
TD 65536
SOLVENT DMSO
NS 500
DS 0
SWH 17985.611 Hz
FIDRES 0.274439 Hz
AQ 1.8219508 sec
RG 32768
DW 27.800 usec
DE 6.00 usec
TE 300.0 K
D1 4.00000000 sec
d11 0.03000000 sec
d12 0.00002000 sec
======== CHANNEL f1 ========
NUC1 13C
P1 14.00 usec
PL1 -6.00 dB
SFO1 75.4099117 MHz
======== CHANNEL f2 ========
CPDPRG2 waltz16
NUC2 1H
PCPD2 80.00 usec
PL2 3.00 dB
PL12 23.00 dB
PL13 23.00 dB
SFO2 299.8711995 MHz
F2 - Processing parameters
SI 32768
SF 75.4023710 MHz
WDW EM
SSB 0
LB 1.00 Hz
GB 0
PC 1.40
NH2
CN
H3COOC
N
NH
O
NC
200 180 160 140 120 100 80 60 40 20 ppm

^{13}C NMR of 5h

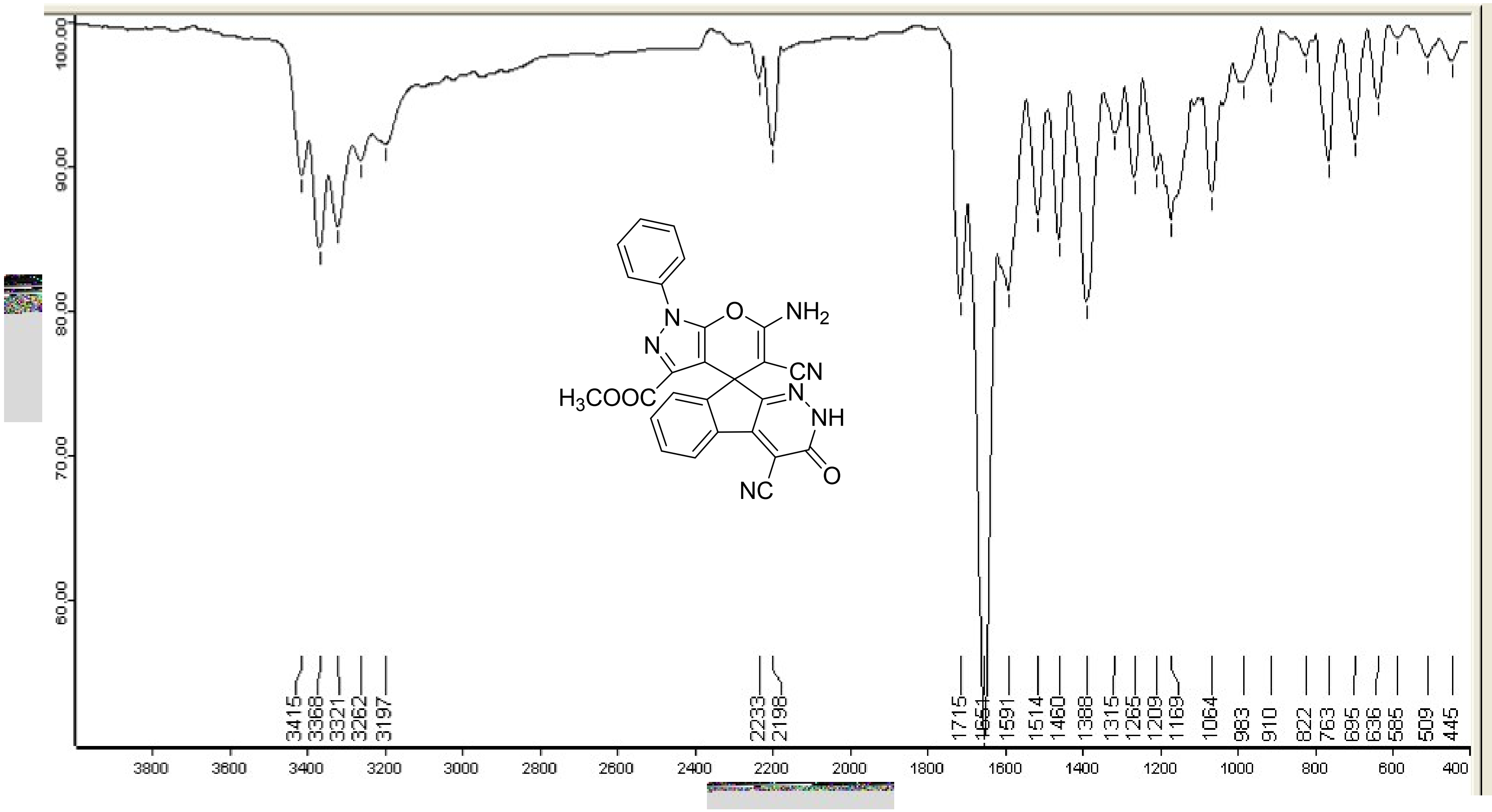
100,00
90,00
80,00
70,00
60,00
3415
3368
3321
3262
3197
2233
2198
1715
1651
1591
1514
1460
1388
1315
1265
1209
1169
1064
983
910
822
763
695
636
585
509
445
3800
3600
3400
3200
3000
2800
2600
2400
2200
2000
1800
1600
1400
1200
1000
800
600
400
NH2
CN
H3COOC
NH
NC

IR of 5h

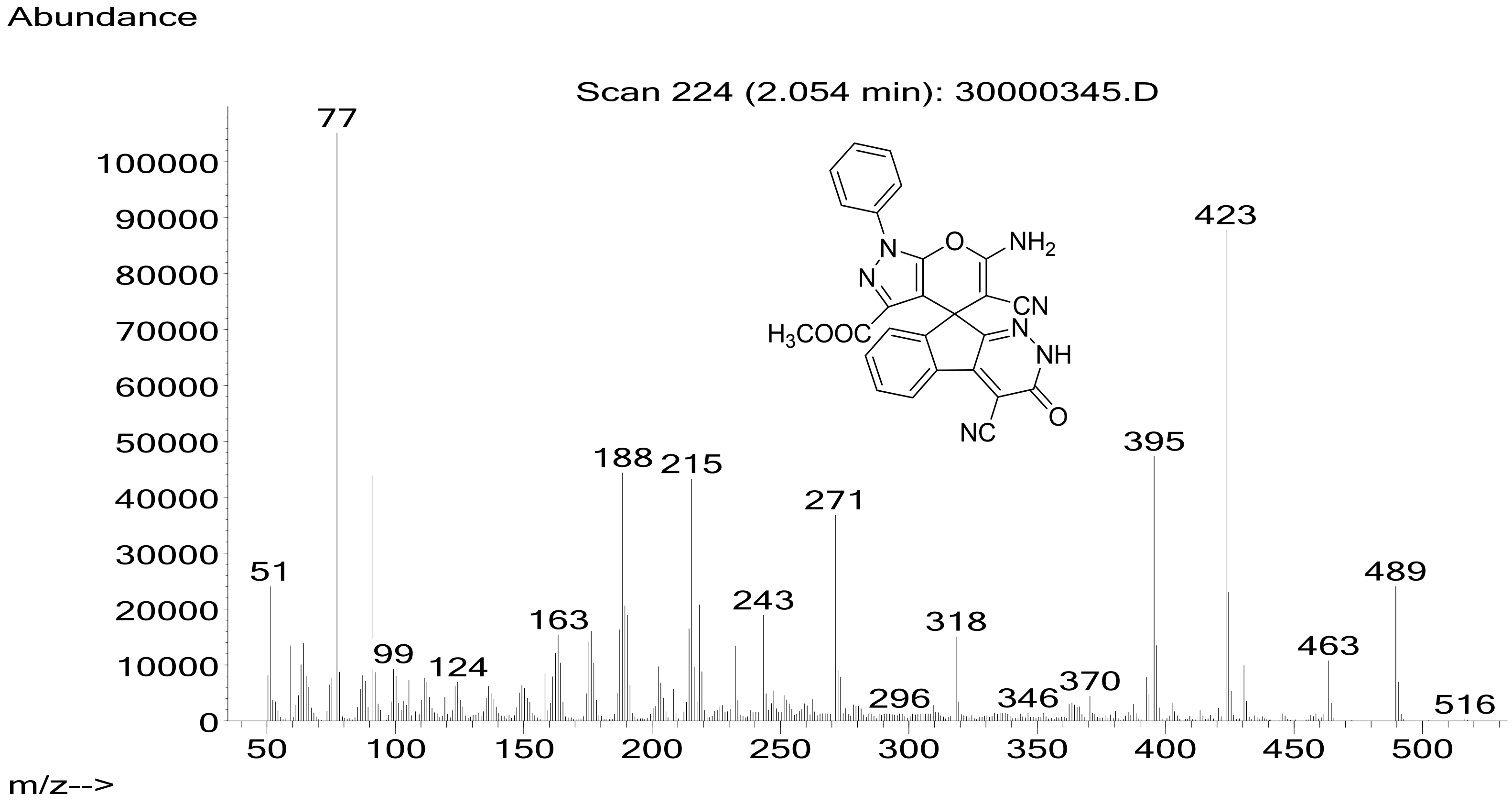

Abundance
Scan 224 (2.054 min): 30000345.D
100000
90000
80000
70000
60000
50000
40000
30000
20000
10000
0
77
51
99
124
163
188
215
243
271
296
318
346
370
395
423
463
489
516
50
100
150
200
250
300
350
400
450
500
m/z-->
NH2
O
N
N
CN
H3COOC
N
NH
O
NC

MS of 5h

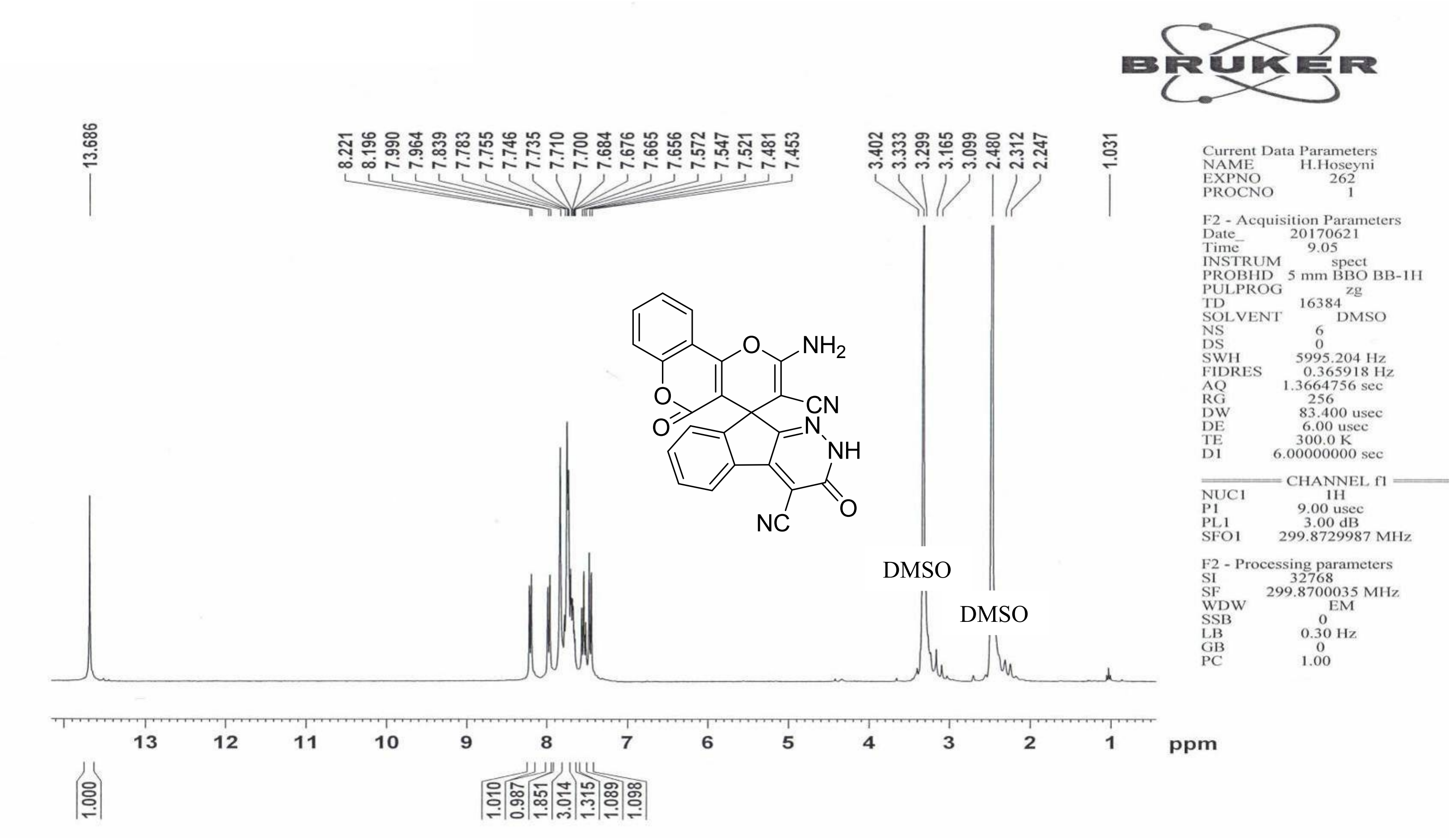
BRUKER
13.686
8.221
8.196
7.990
7.964
7.839
7.783
7.755
7.746
7.735
7.710
7.700
7.684
7.676
7.665
7.656
7.572
7.547
7.521
7.481
7.453
3.402
3.333
3.299
3.165
3.099
2.480
2.312
2.247
1.031
NH2
CN
N
NH
O
NC
DMSO
DMSO
Current Data Parameters
NAME H.Hoseyni
EXPNO 262
PROCNO 1
F2 - Acquisition Parameters
Date_ 20170621
Time 9.05
INSTRUM spect
PROBHD 5 mm BBO BB-1H
PULPROG zg
TD 16384
SOLVENT DMSO
NS 6
DS 0
SWH 5995.204 Hz
FIDRES 0.365918 Hz
AQ 1.3664756 sec
RG 256
DW 83.400 usec
DE 6.00 usec
TE 300.0 K
D1 6.00000000 sec
CHANNEL f1
NUC1 1H
P1 9.00 usec
PL1 3.00 dB
SFO1 299.8729987 MHz
F2 - Processing parameters
SI 32768
SF 299.8700035 MHz
WDW EM
SSB 0
LB 0.30 Hz
GB 0
PC 1.00
13
12
11
10
9
8
7
6
5
4
3
2
1
ppm
1.000
1.010
0.987
1.851
3.014
1.315
1.089
1.098

^{1}H NMR of 5i

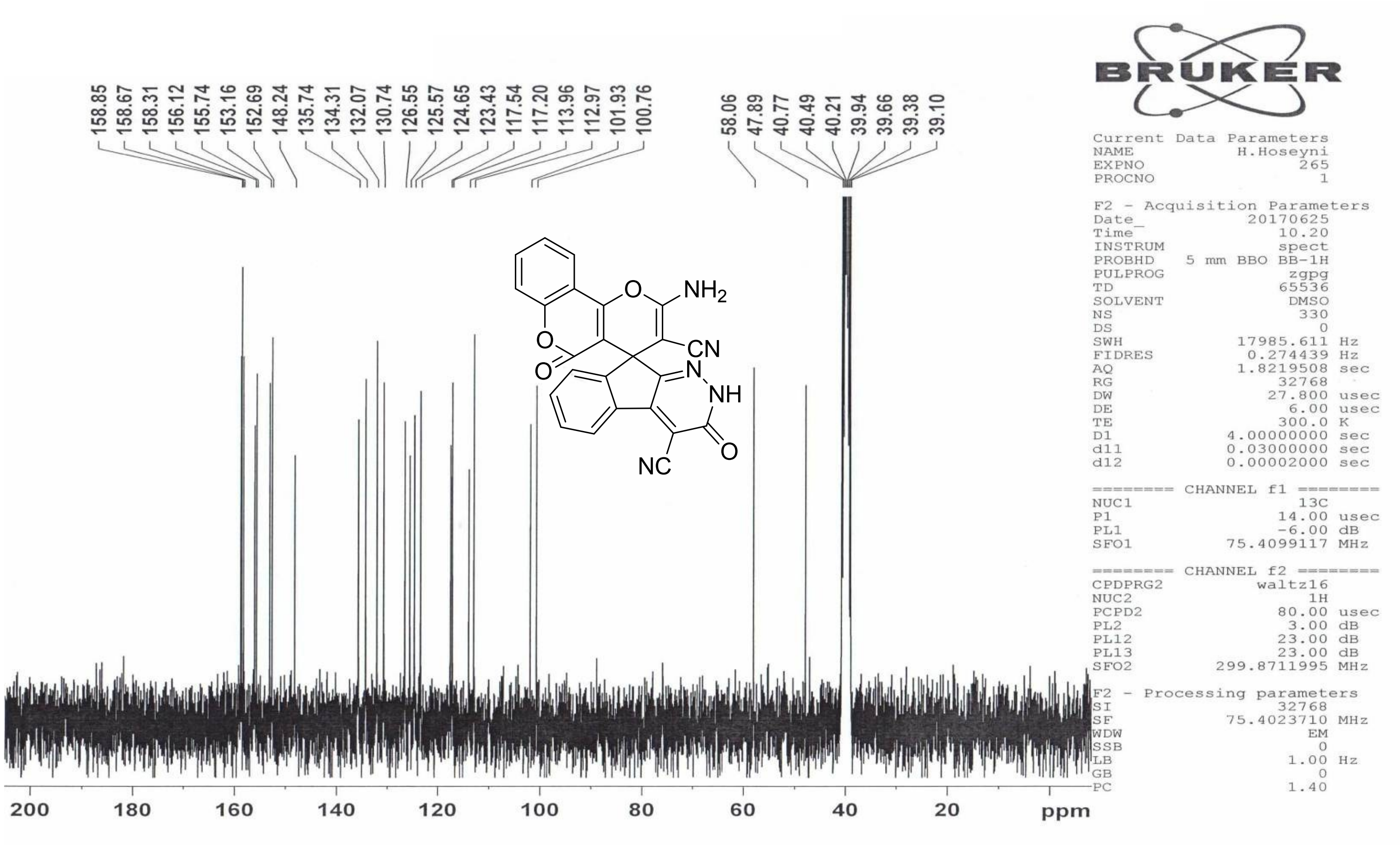

158.85
158.67
158.31
156.12
155.74
153.16
152.69
148.24
135.74
134.31
132.07
130.74
126.55
125.57
124.65
123.43
117.54
117.20
113.96
112.97
101.93
100.76
58.06
47.89
40.77
40.49
40.21
39.94
39.66
39.38
39.10
BRUKER
Current Data Parameters
NAME H.Hoseyni
EXPNO 265
PROCNO 1
F2 - Acquisition Parameters
Date_ 20170625
Time 10.20
INSTRUM spect
PROBHD 5 mm BBO BB-1H
PULPROG zgpg
TD 65536
SOLVENT DMSO
NS 330
DS 0
SWH 17985.611 Hz
FIDRES 0.274439 Hz
AQ 1.8219508 sec
RG 32768
DW 27.800 usec
DE 6.00 usec
TE 300.0 K
D1 4.00000000 sec
d11 0.03000000 sec
d12 0.00002000 sec
======== CHANNEL f1 ========
NUC1 13C
P1 14.00 usec
PL1 -6.00 dB
SFO1 75.4099117 MHz
======== CHANNEL f2 ========
CPDPRG2 waltz16
NUC2 1H
PCPD2 80.00 usec
PL2 3.00 dB
PL12 23.00 dB
PL13 23.00 dB
SFO2 299.8711995 MHz
F2 - Processing parameters
SI 32768
SF 75.4023710 MHz
WDW EM
SSB 0
LB 1.00 Hz
GB 0
PC 1.40
200
180
160
140
120
100
80
60
40
20
ppm

^{13}C NMR of 5i

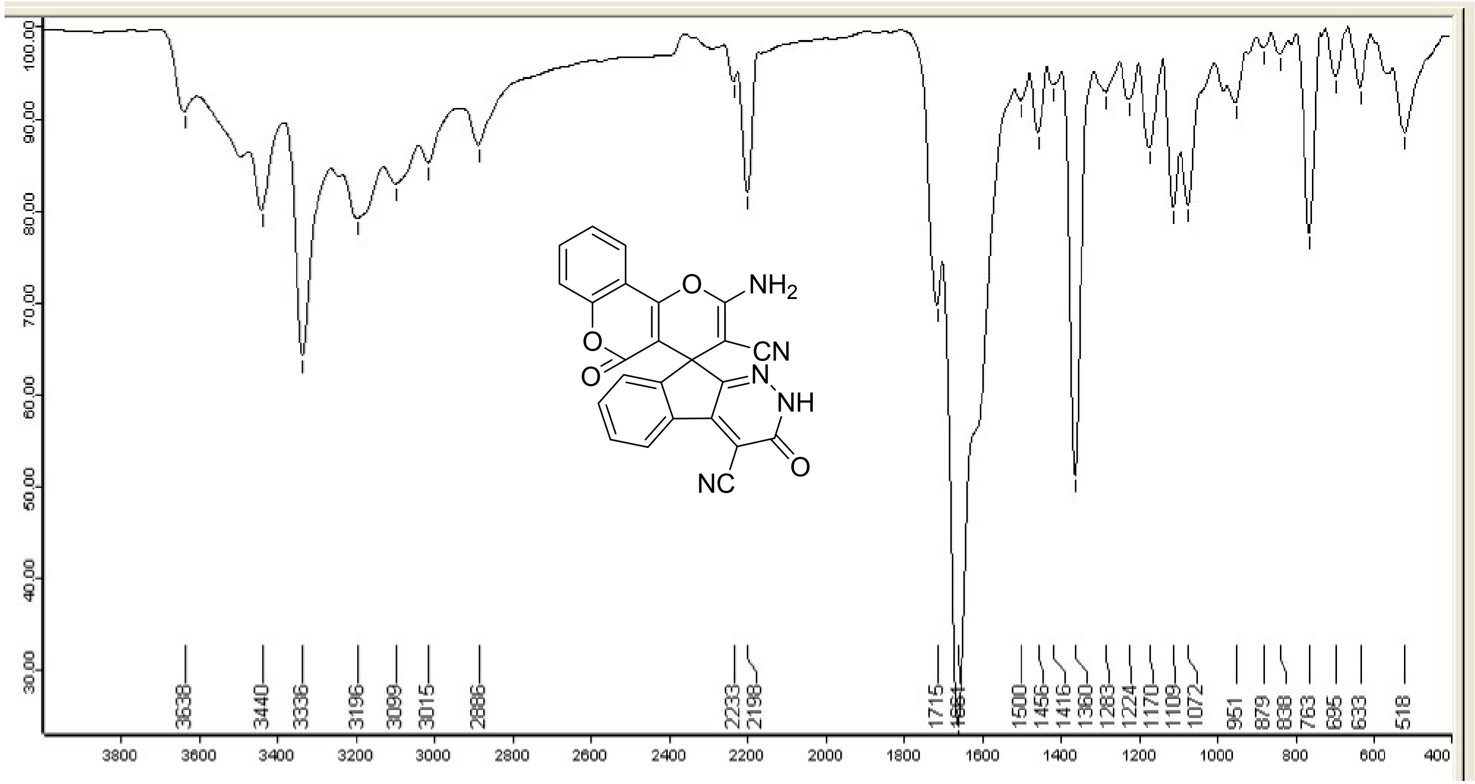
100.00
90.00
80.00
70.00
60.00
50.00
40.00
30.00
3638
3440
3336
3196
3099
3015
2886
2233
2198
1715
1661
1500
1456
1416
1360
1263
1224
1170
1109
1072
951
879
838
763
695
633
518
3800
3600
3400
3200
3000
2800
2600
2400
2200
2000
1800
1600
1400
1200
1000
800
600
400
O
NH2
O
O
CN
N
NH
NC
O

IR of 5i

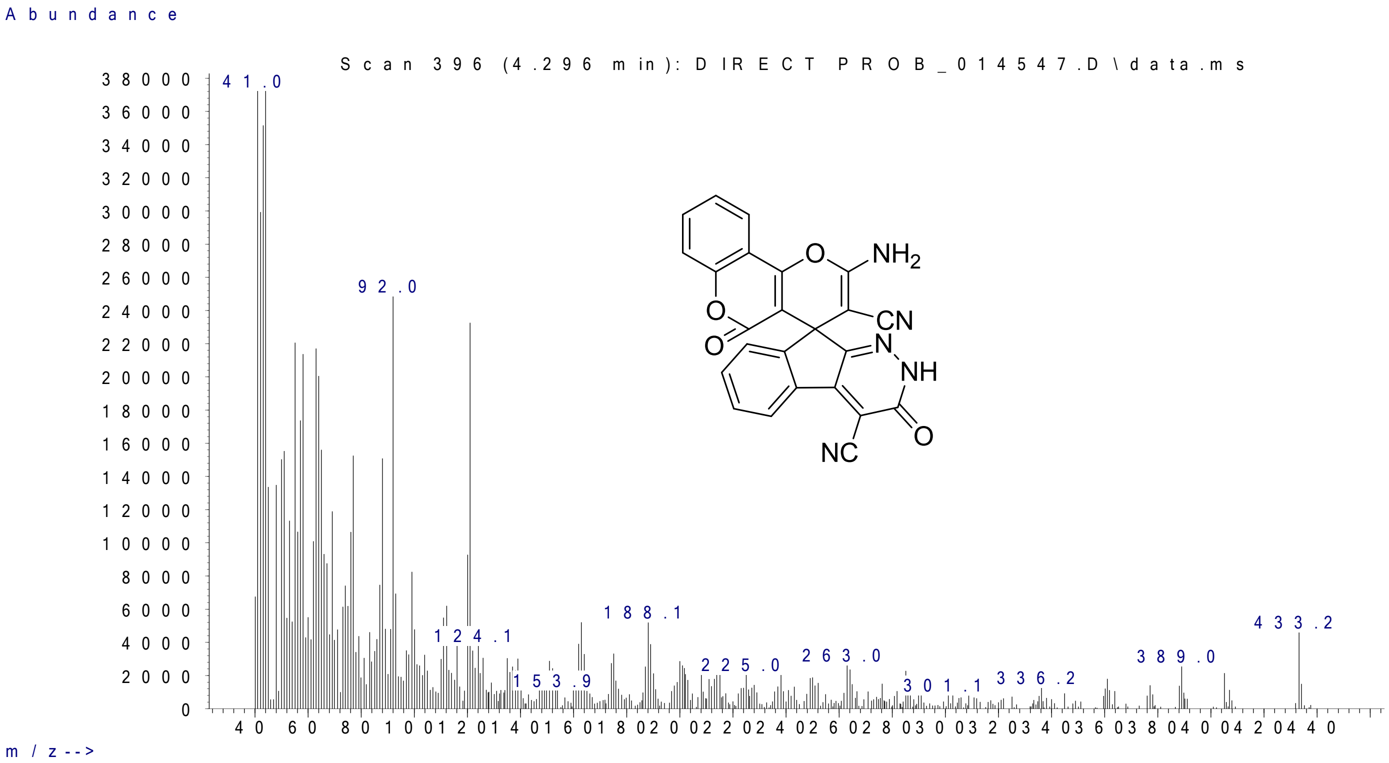
Abundance
Scan 396 (4.296 min): DIRECT PROB_014547.D\data.ms
41.0
92.0
124.1
153.9
188.1
225.0
263.0
301.1
336.2
389.0
433.2
m/z-->
NH2
CN
NH
NC
O

MS of 5i

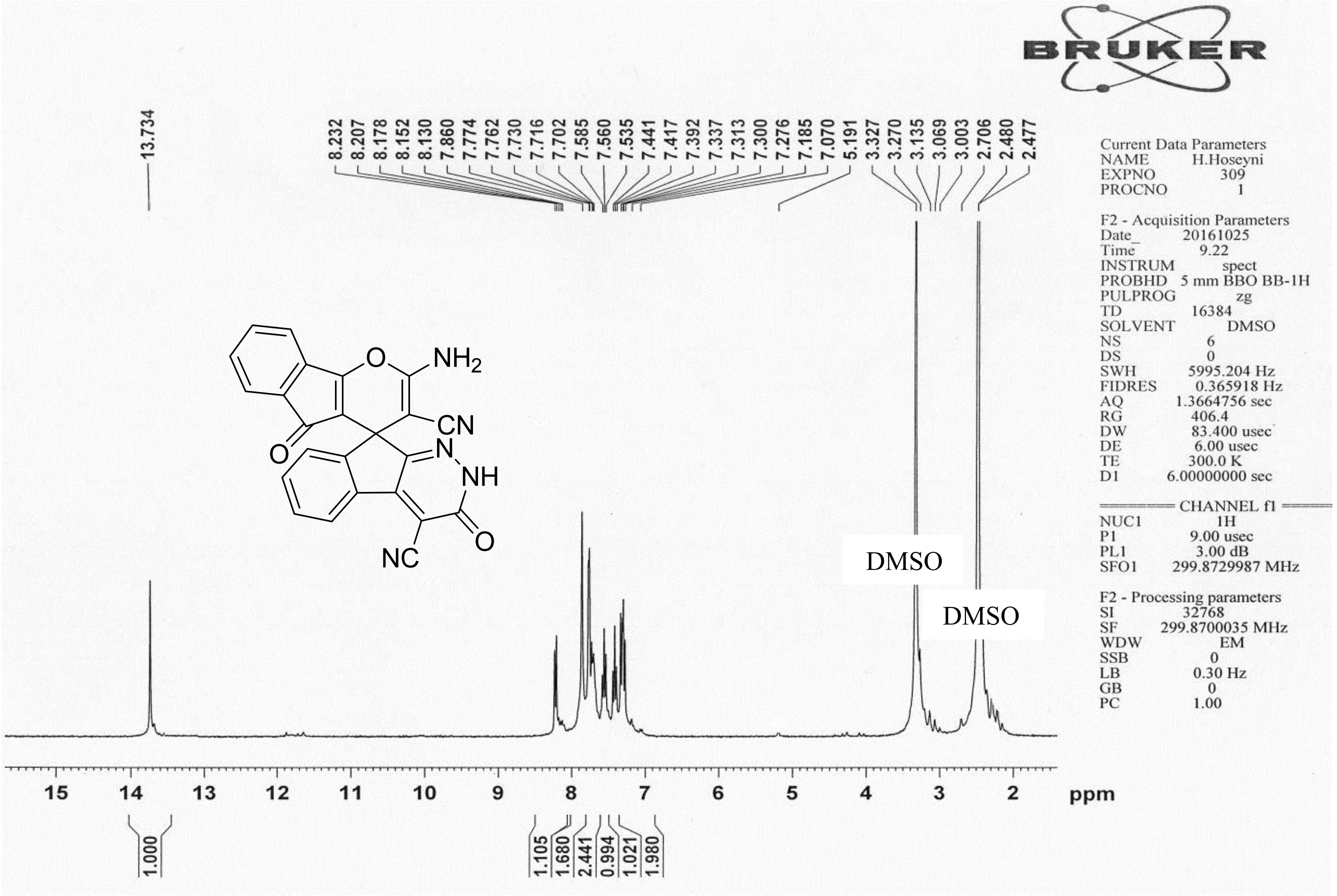
BRUKER
13.734
8.232
8.207
8.178
8.152
8.130
7.860
7.774
7.762
7.730
7.716
7.702
7.585
7.560
7.535
7.441
7.417
7.392
7.337
7.313
7.300
7.276
7.185
7.070
5.191
3.327
3.270
3.135
3.069
3.003
2.706
2.480
2.477
Current Data Parameters
NAME H.Hoseyni
EXPNO 309
PROCNO 1
F2 - Acquisition Parameters
Date_ 20161025
Time 9.22
INSTRUM spect
PROBHD 5 mm BBO BB-1H
PULPROG zg
TD 16384
SOLVENT DMSO
NS 6
DS 0
SWH 5995.204 Hz
FIDRES 0.365918 Hz
AQ 1.3664756 sec
RG 406.4
DW 83.400 usec
DE 6.00 usec
TE 300.0 K
D1 6.00000000 sec
CHANNEL f1
NUC1 1H
P1 9.00 usec
PL1 3.00 dB
SFO1 299.8729987 MHz
F2 - Processing parameters
SI 32768
SF 299.8700035 MHz
WDW EM
SSB 0
LB 0.30 Hz
GB 0
PC 1.00
NH2
O
CN
N
NH
O
NC
O
DMSO
DMSO
15 14 13 12 11 10 9 8 7 6 5 4 3 2 ppm
1.000
1.105
1.680
2.441
0.994
1.021
1.980

^{1}H NMR of 5j

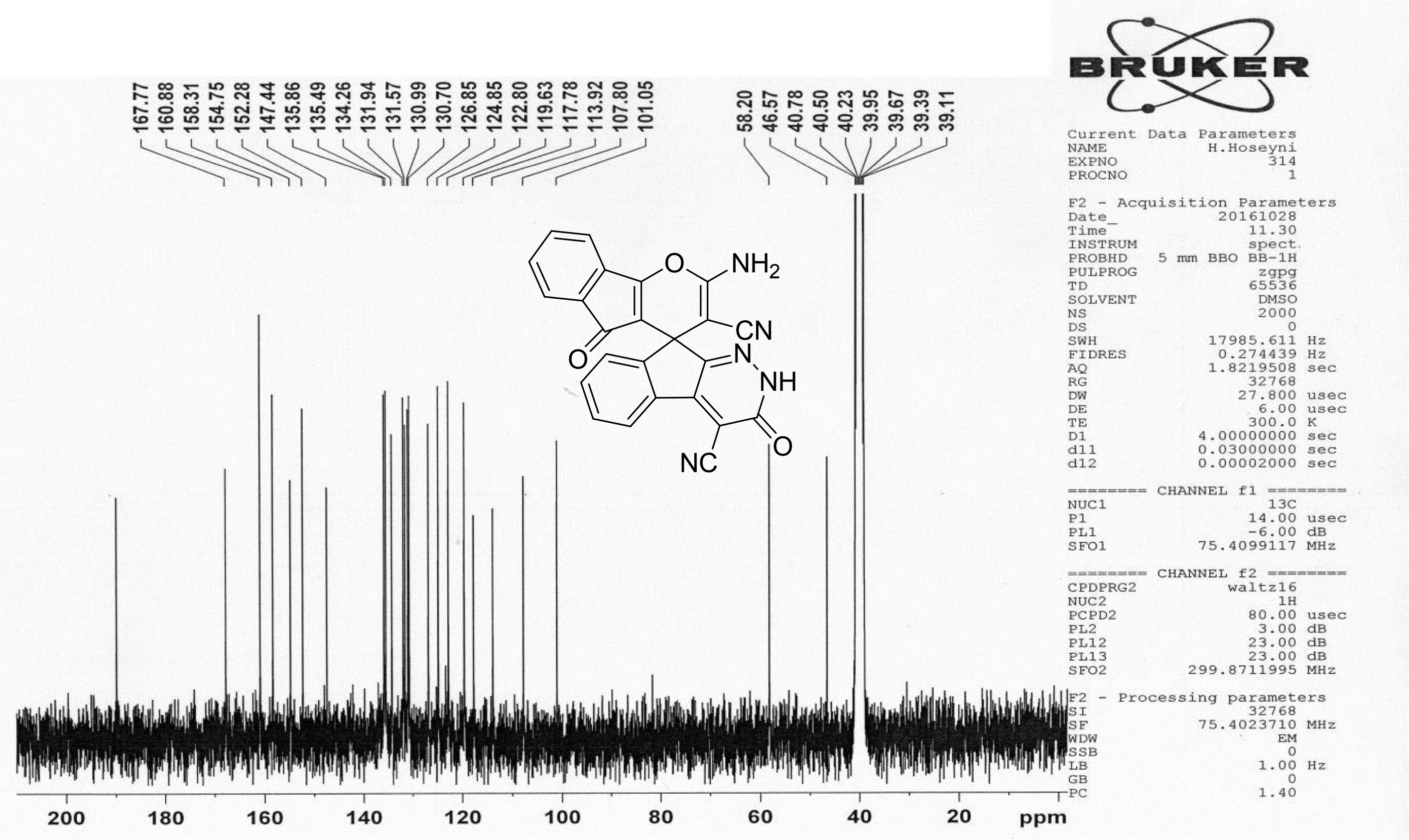

^{13}C NMR of 5j

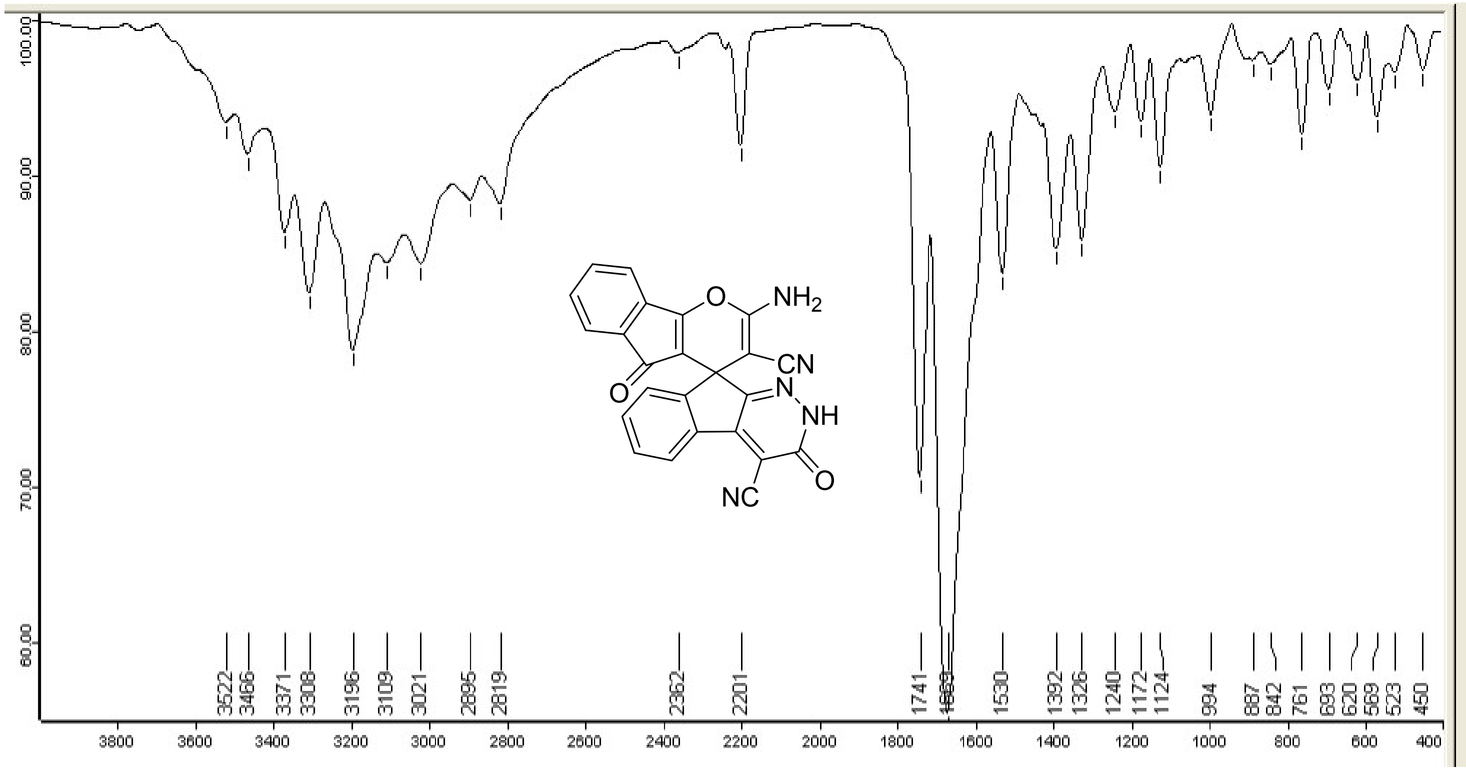
100.00
90.00
80.00
70.00
60.00
3522
3466
3371
3308
3196
3109
3021
2895
2819
2362
2201
1741
1530
1392
1326
1240
1172
1124
994
887
842
761
693
620
569
523
450
3800
3600
3400
3200
3000
2800
2600
2400
2200
2000
1800
1600
1400
1200
1000
800
600
400
O
NH2
CN
O
N
NH
O
NC

IR of 5j
